# Home-Delivered Meals and Nursing Home Placement Among People With Self-Reported Dementia: A Pilot Pragmatic Clinical Trial

**DOI:** 10.1001/jamanetworkopen.2023.47195

**Published:** 2023-12-20

**Authors:** Kali S. Thomas, Jen Bunker, Emily Gadbois, Michelle Hilgeman, Ellen McCreedy, Whitney Mills, Katherine A. Ornstein, Jennifer Reckrey, Roee Gutman

**Affiliations:** 1 Brown University School of Public Health, Providence, Rhode Island; 2 Providence VA Medical Center, Providence, Rhode Island; 3 Research and Development Service, Tuscaloosa VA Medical Center, Tuscaloosa, Alabama; 4 Department of Psychology, The University of Alabama, Tuscaloosa; 5 Alabama Research Institute on Aging, The University of Alabama, Tuscaloosa; 6 Department of Medicine, Division of Gerontology, Geriatrics, and Palliative Care, The University of Alabama at Birmingham; 7 Johns Hopkins School of Nursing, Baltimore, Maryland; 8 Icahn School of Medicine at Mount Sinai, New York, New York

## Abstract

**Importance:**

Home-delivered meals promote food security and independence among homebound older adults. However, it is unclear which of the 2 predominant modes of meal delivery, daily-delivered vs mailed (or drop-shipped) frozen meals, promotes community living for homebound older adults with dementia.

**Objective:**

To assess the risk of nursing home admission within 6 months between homebound individuals receiving daily-delivered vs drop-shipped frozen meals.

**Design, Setting, and Participants:**

This pilot, multisite, 2-arm, pragmatic clinical trial included older adults with self-reported dementia on waiting lists for meals at 3 Meals on Wheels (MOW) programs in Texas and Florida between April 7 and October 8, 2021, to assess time to nursing home placement.

**Interventions:**

Participants were randomized to receive either meals delivered by an MOW driver or frozen meals that were mailed to participants’ homes every 2 weeks. Participants received their assigned intervention for up to 6 months.

**Main Outcomes and Measures:**

The primary study outcome was days from randomization to a Minimum Data Set nursing home admission assessment within 6 months. Feasibility of conducting this type of study was examined by tracking enrollment, examining baseline characteristics, monitoring participants’ intervention fidelity, measuring the proportion of participants linked with Centers for Medicare & Medicaid Services (CMS) data, and analyzing the primary study outcome.

**Results:**

Among 325 eligible participants who were randomized, 243 enrolled in the study (mean [SD] age, 81 [8.0] years; 152 (62.6%) were female): 128 to the daily-delivered meals group and 115 to the drop-shipped frozen meals group; 119 participants (49.0%) lived alone. Among the total participants enrolled, 227 (93.4%) were linked deterministically to their CMS data; probabilistic methods were used to link the remaining 16 participants (6.6%). At 6 months from randomization, 160 participants (65.8%) were still receiving meals, and 25 (10.1%; 95% CI, 6.3%-14.0%) were admitted to a nursing home. After adjusting for sex, race and ethnicity, age, program, and living arrangement and the use of death as a censoring event, the adjusted log hazard ratio of nursing home placement between daily-delivered and drop-shipped frozen meals was −0.67 (95% CI, −1.52 to 0.19).

**Conclusions and Relevance:**

This pilot randomized clinical trial demonstrated the feasibility of enrolling participants with self-reported dementia on waiting lists at MOW programs, linking their data, and evaluating outcomes. While this pilot study was not powered to detect meaningful, statistically significant differences in nursing home placement, its feasibility and initial results warrant exploration in a follow-on, adequately powered trial.

**Trial Registration:**

ClinicalTrials.gov Identifier: NCT04850781

## Key Points

**Question:**

Do 2 predominant modes of home-delivered meals (daily-delivered meals and mailed, or drop-shipped, frozen meals) differentially delay nursing home placement for homebound older adults with self-reported dementia?

**Findings:**

This pilot pragmatic clinical trial included 243 homebound older adults with self- or proxy-reported dementia found a lower although nonsignificant likelihood of nursing home placement among those receiving daily-delivered meals compared with those receiving drop-shipped frozen meals. However, the feasibility of conducting a clinical trial with Meals on Wheels programs was shown.

**Meaning:**

These results suggest that further exploration in an adequately powered clinical trial is possible and warranted.

## Teaser

This randomized clinical trial analyzes whether 2 predominant modes of home-delivered meals differentially delay nursing home placement for older adults who are homebound with self-reported dementia.

## Introduction

An overwhelming majority (81%) of the 5.3 million older adults with dementia live in the community, and an estimated 25% to 30% live alone.^1^ Community-dwelling older adults with dementia have high rates of unmet care needs (eg, assistance with eating, grocery shopping, and preparing meals),^2,3^ which puts them at increased risk of food insecurity^4^ and nursing home placement.^5^

Home-delivered meals, by organizations such as Meals on Wheels (MOW), promote food security, socialization, and independence among older adults who are homebound. Partially funded by Title III of the Older Americans Act, about 5000 programs served more than 880 000 older adults in 2019, over half of whom lived alone, were older than 75 years, and indicated that the meals provided more than half of their total food for the day.^6^ Evidence suggests that home-delivered meals are associated with the health and the health care use of older adults who are homebound and food-insecure,^7,8,9,10,11,12^ and 40% of people who receive home-delivered meals have a dementia diagnosis.^8^ Informed by this evidence, health care entities are increasingly providing home-delivered meals to their patients. In 2022, almost 2 million Medicare beneficiaries were enrolled in a Medicare Advantage plan that offered a long-term meal benefit,^13^ and in 2020, 42 states covered home-delivered meals in their Medicaid program.^14^

Historically, home-delivered meals have been provided to clients in their homes 5 to 7 days each week (referred to as daily-delivered meals). Meals typically come ready to eat and are delivered by a volunteer or paid driver who may informally socialize with the client and report any concerns about their well-being to the meal program staff. In recent years, the model of mailing 1 to 2 weeks’ worth of frozen meals (referred to as drop-shipped meals) has emerged as a lower-cost alternative. While both models provide the same nutritional benefit, older adults receiving drop-shipped frozen meals do not experience the socialization, assistance, and identification of service needs, which are believed to drive many of the positive outcomes associated with receiving home-delivered meals. In addition, frozen meals require reheating; therefore, older adults receiving frozen meals may not have the convenience of ready-to-eat meals. The additional benefits afforded by the daily-delivered model are likely to be especially important for older adults who are homebound with dementia—a population who experiences social isolation,^15^ loneliness,^16^ malnutrition,^17^ and cognitive challenges that can impact meal preparation.^18^

Therefore, the primary objective of this pilot, pragmatic randomized clinical trial was to assess the association between daily-delivered vs drop-shipped frozen meals and nursing home admission among homebound adults with dementia and to test the feasibility of tracking enrollment, examining baseline characteristics, monitoring participants’ intervention fidelity, measuring the proportion of participants linked with Centers for Medicare & Medicaid Services (CMS) data, and analyzing the primary study outcome.

## Methods

### Trial Design and Oversight

The trial protocol was approved by the Brown University institutional review board and was overseen by a National Institute on Aging–appointed safety officer. The full trial protocol (Supplement 1) has been published elsewhere.^19^ In the original protocol, the requirement for informed consent was waived because it met all 5 conditions based on 45 CFR § 46.116. This study followed the Consolidated Standards of Reporting Trials (CONSORT) reporting guideline for randomized studies.^20^

The pilot study was designed as a multisite, pragmatic, randomized clinical trial to test the feasibility of enrolling people with dementia, randomizing them to 1 of 2 home-delivered meal interventions, monitoring their fidelity to the intervention, linking them to data from the CMS, and analyzing their outcomes. Enrollment began April 7 and ended October 8, 2021. Final, 6-month follow-up observations concluded April 8, 2022. The pilot study included a qualitative substudy in which a subset of participants and a subset of caregivers participated in in-depth telephone interviews. (The approach and results of the substudy are published elsewhere.^21^)

### Participants

Participants were aged 66 years or older on waiting lists at 3 MOW programs in Florida (1 program) and Texas (2 programs). We included participants who had self-reported or proxy-reported dementia during the programs’ intake assessment—a process completed for all individuals who request meals from these 3 MOW programs. Specifically, we relied on a question in the programs’ intake assessments: “Has a doctor or other health care professional told you that you suffer from memory loss, cognitive impairment, any type of dementia, or Alzheimer’s disease?” Prospective participants who resided outside the program’s daily service area were excluded from participation.

### Randomization

The statistician (R.G.) generated a 1:1 stratified, permuted, block randomization scheme, in which each block comprised 6 participants.^22^ The 3 strata were each of the MOW programs. Participants were randomly assigned by the project coordinator to 1 of the 2 interventions using the randomization scheme. The statistician was blinded to randomization assignments; participants were not.

### Interventions

Participants randomized to daily-delivered meals were to receive 5 lunchtime meals delivered to their homes, 2 to 5 times per week, by an MOW program volunteer or a paid driver who interacted with the participant and provided an informal wellness check (all employees/volunteers are asked by programs to report any concerns as part of their program’s standard practice). Programs were not given any specific instructions about how to deliver these meals; rather, given the pragmatic nature of this study, programs were asked to provide meals as they would in usual practice. Participants randomized to drop-shipped meals received 10 frozen meals, mailed via FedEx, to their home every 2 weeks from TRIO Community Meals. Meals in both arms met the same nutritional standards (ie, adhered to current Dietary Guidelines for Americans^23^ and provided one-third of the Dietary Reference Intakes^24^). Meals paid for by the study were provided for up to 6 months; after 6 months, participants could choose to continue receiving meals provided by the MOW programs.

### Measures and Primary Study Outcome

To examine feasibility, we tracked enrollment, examined baseline characteristics between the randomized arms, monitored participants’ fidelity to the intervention, measured the proportion of participants who we were able to link with CMS data, and analyzed the primary study outcome. Participants’ demographics (ie, age, race and ethnicity, sex, and living arrangement) were obtained by the programs during the usual intake assessment process and shared with the research team. Racial and ethnic groups were self-reported or proxy-reported and included participants who were Black, Latino or Hispanic, White, and other race and ethnicity, which included Asian, multiracial, and “other,” as reported by the programs. Results for those categorized as other were combined because the small numbers could result in participant identification. Fidelity (eg, received meals for 6 months, discontinued meals, and reasons for discontinuing meals) came from the programs’ administrative data. The primary study outcome was the number of days from randomization to nursing home admission, ascertained using a Minimum Data Set admission assessment (completed for all new nursing home residents regardless of payer or intent of stay), within 6 months of randomization.

### Adverse Events

Participant deaths, while expected and not related to the intervention, were tracked by the research team. All deaths were reported to the safety officer, the Brown University institutional review board, and the National Institute on Aging program officer for review and oversight.

### Linking Data

Outcome data were obtained by linking enrolled participants with CMS administrative data, which were obtained through a study-specific data use agreement that included the 2021 to 2022 Medicare Master Beneficiary Summary File (MBSF) and the 2021 to 2022 Minimum Data Set.^25^ To link participants, we sent 2 finder files to the CMS contractor. One file included a participant ID and social security number. The second file included a participant ID, surname, zip code, date of birth, and sex. The contractor was able to link 227 participants to the MBSF. To link the remaining 16 participants, of whom the CMS contractor was unable to manually link, we relied on a bayesian probabilistic linkage algorithm.^26,27^ This algorithm used enrollees’ zip+4 code, date of birth, and sex to identify possible records in the MBSF. To address any potential errors in linked records from the probabilistic algorithms,^28^ we used a multiple imputation procedure to propagate the errors in the linkage among the 16 unlinked individuals.^27,29^ We generated 200 datasets in which up to 243 individuals were linked to records in CMS data (227 deterministically and up to 16 probabilistically).

### Sample Size and Power

Given that this was a pilot study, the target sample size was not based on power but rather on available funds to provide meals for 6 months to enrolled participants. Assuming that the proportions of participants who would be admitted to nursing homes in 6 months were 11% in the daily-delivered arm and 13% in the drop-shipped frozen meals arm, among 243 participants (115 in the drop-shipped frozen arm and 128 in the daily-delivered arm), we had 80% power to detect a hazard ratio of 0.33.

### Statistical Analysis

We used an intention-to-treat analysis; all participants were observed until death, outcome event, or end of the 6-month follow-up. We examined baseline demographic characteristics using data provided by programs. We examined differences in our primary study outcome, time to nursing home admission, between the 2 interventions using a Cox proportional hazards regression model that adjusted for baseline covariates (ie, age, race and ethnicity, sex, living arrangement, and indicator for the program) to address possible imbalances across arms.^30^ Formally, let *h*(*t*) = *h_0_*(*t*) × exp (*γW_i_ + β′X_i_*) be the hazard function, in which *t* is the time to nursing home admission, *X_i_* is a set of baseline covariates for participant *i*, *W_i_* is an indicator for daily-delivered meals for participant *i*, *h_0_*(*t*) is the baseline hazard, β is a set of unknown parameters, and γ is the conditional log hazard ratio between daily-delivered and drop-shipped frozen meals. The conditional log hazard, *γ,* was estimated within each of the 200 linked datasets, and point and interval estimates were obtained using multiple imputation combining rules.^29,31^ The combination rules for the log-rank statistic were used to compare the survival curves across imputations.^32^ To ensure that mortality was similar across arms, we compared the differences in mortality using the difference in proportions test. As a sensitivity analysis, we also compared the 2 arms only among participants who were manually linked by the CMS contractor. All of the analyses were conducted with the R survival package, version 4.3.1. (R Project for Statistical Computing).^33,34^ Probabilistic linking^26^ of CMS records was performed with the BRL package (R Project for Statistical Computing). A 2-sided *P* < .05 was considered statistically significant.

## Results

**Figure 1.**
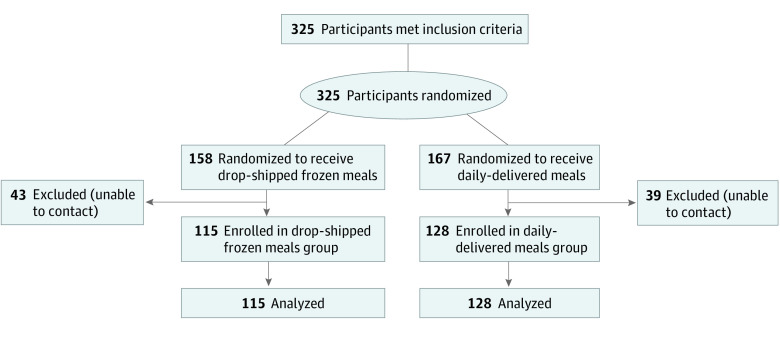
CONSORT Diagram CONSORT indicates Consolidated Standards of Reporting Trials.

Study-specific IDs of 325 prospective enrollees who met inclusion criteria were sent to the research team and were randomized between April and October 2021 (Figure 1). The target sample size was 236 participants, and 243 participants (mean [SD] age, 81 [8.0] years; 152 [62.6%] female; 91 [37.5%] male) were ultimately enrolled in the study (Table 1); 128 randomized to daily-delivered meals and 115 to drop-shipped frozen meals. The 82 individuals who were randomized but not enrolled were unable to be reached by the participating programs’ staff after the research team randomized them to a study arm. Among participants enrolled in both groups, 43 (17.7%) were Black; 62 (25.5%), were Latino or Hispanic; 128 (52.7%) were White; and 10 (4.1%), were categorized as “other.” A total of 119 participants (49.0%) lived alone. Among the 3 programs, there were 59 enrollees (24.3%) at site 1; 78 (32.1%), site 2; and 106 (43.6%), site 3. Race and ethnicity and program were the only statistically significant differences between individuals who were enrolled and those who were randomized but not enrolled (Table 1).

**Table 1.** Demographic Characteristics of Individuals Randomized by Enrollment Status

Characteristic	Eligible participants, No. (%) (N = 325)	
	Enrolled (n = 243)	Not enrolled (n = 82)
Age group, y		
66-74	63 (25.9)	30 (36.6)
75-84	96 (39.5)	33 (40.2)
≥85	84 (34.6)	19 (23.2)
Sex		
Female	152 (62.6)	45 (54.9)
Male	91 (37.5)	37 (45.1)
Race and ethnicity^a^		
Black	43 (17.7)	10 (12.2)
Latino or Hispanic	62 (25.5)	7 (8.5)
White	128 (52.7)	63 (76.8)
Other^b^	10 (4.1)	2 (2.4)
Lived alone	119 (49.0)	44 (53.7)
Program^a^		
Site 1	59 (24.3)	44 (53.7)
Site 2	78 (32.1)	26 (31.7)
Site 3	106 (43.6)	12 (14.6)

^a^ The difference between individuals who were enrolled and those who were randomized but not enrolled was significant at *P* < .05.

^b^ Other race and ethnicity includes Asian, multiracial, and “other,” as reported by the programs. Results for those categorized as other were combined because the small numbers could result in participant identification.

For the total participants enrolled, 227 (93.4%) were linked deterministically to their CMS data; 16 participants (6.6%) could not be deterministically linked to the CMS data (6 in the daily-delivered meals group and 10 in the drop-shipped frozen meals group). There were no statistically significant differences in baseline characteristics between linked and nonlinked participants.

Comparing participants by study arm, the majority of participants were female (78 [67.8%] for drop-shipped frozen meals and 74 [57.8%] for daily-delivered meals) and White (64 [55.7%] for drop-shipped frozen meals and 64 [50.0%] for daily-delivered meals) (Table 2). In the drop-shipped frozen meals and daily-delivered meals groups, 18% of participants in both study groups were Black (22 [19.1%] vs 21 [16.4%]), and 21% were Latino or Hispanic (25 [21.7%] for the drop-shipped frozen meals group and 37 [28.9%] for the daily-delivered meals group); 66 participants (57.4%) assigned to drop-shipped frozen meals lived alone compared with 53 (41.4%) assigned to receive daily-delivered meals. At 6 months from randomization, 160 participants (65.8%) were still receiving meals. According to the programs’ administrative data, 88 participants (68.8%) assigned to receive daily-delivered meals completed 6 months of receiving meals compared with 72 (62.6%) assigned to receive drop-shipped frozen meals.

**Table 2.** Baseline Characteristics of Enrolled Participants by Study Arm^a^

Characteristic	Participants, No. (%) (N = 243)	
	Drop-shipped frozen meals (n = 115)	Daily-delivered meals (n = 128)
Age group, y		
66-74	27 (23.5)	36 (28.1)
75-84	46 (40.0)	50 (39.1)
≥85	42 (36.5)	42 (32.8)
Sex		
Female	78 (67.8)	74 (57.8)
Male	37 (32.2)	54 (42.2)
Race and ethnicity		
Black	22 (19.1)	21 (16.4)
Latino or Hispanic	25 (21.7)	37 (28.9)
White	64 (55.7)	64 (50.0)
Other^b^	4 (3.5)	6 (4.7)
Lived alone	66 (57.4)	53 (41.4)

^a^ Data were derived from sites’ intake assessments completed for all new people added to the waiting list.

^b^ Other race and ethnicity includes Asian, multiracial, and “other,” as reported by the programs. Results for those categorized as other were combined because the small numbers could result in participant identification.

Two participants who were linked by the CMS contractor died before the randomization date and were removed from the remaining analyses. A pooled analysis of the 200 probabilistically linked datasets (for participant characteristics, see the eTable in Supplement 2) revealed that 6 months after randomization, 25 participants (10.1%; 95% CI, 6.3%-14.0%) had been admitted to a nursing home. Participants in the daily-delivered meals arm were less likely to be admitted to a nursing home compared with the drop-shipped frozen meals arm, although the result was not significant (difference, 6.0 percentage points; 95% CI, −14.0 to 1.5 percentage points). In addition, 17 participants (7.1%; 95% CI, 3.9%-10.4%) had died. The difference in mortality between the daily-delivered meals and the drop-shipped frozen meals arms was not statistically significant (0.1 percentage point; 95% CI, −6.0 to 6.0 percentage points).

**Figure 2.**
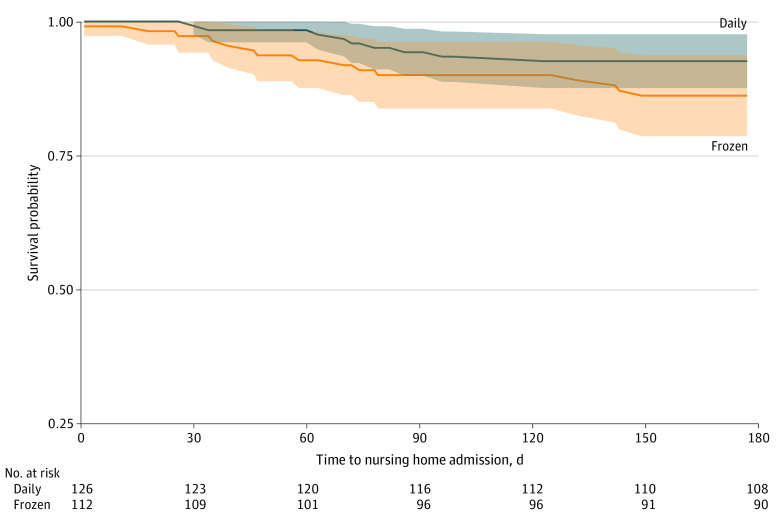
Time From Randomization to Nursing Home Placement by Study Arm Survival probability for mortality and nursing home placement were derived from Centers for Medicare & Medicaid Services data and observed 6 months from randomization (hazard ratio, −0.67; 95% CI, −1.52 to 0.19). The number at risk represents the average across linkage imputations. Not all 16 individuals were linked in every imputation, resulting in fewer than 241 participants overall. Shaded areas indicate 95% CIs. Daily indicates daily-delivered meals; frozen, drop-shipped frozen meals.

Figure 2 displays the Kaplan-Meier curves for each arm. After adjustments for age, sex, race and ethnicity, living arrangement, and program, the conditional log hazard ratio of nursing home placement between the 2 arms was −0.67 (95% CI, −1.52 to 0.19), suggesting that participants receiving daily-delivered meals had a lower, although nonsignificant, instantaneous likelihood of nursing home placement compared with participants who received drop-shipped frozen meals. Performing the analysis only among participants who were manually linked by the CMS contractor produced similar nonsignificant results, with an adjusted log hazard ratio of −0.73 (95% CI, −1.58 to 0.13) between participants receiving daily-delivered meals and those receiving drop-shipped frozen meals.

## Discussion

This pilot study demonstrated the feasibility of implementing a pragmatic randomized clinical trial among participants with self- or proxy-reported dementia on waiting lists at MOW programs. We also showed the ability to link participants with their CMS data, monitor fidelity, and measure the time to nursing home placement. We observed that participants who received daily-delivered meals had lower rates of nursing home placement within 6 months compared with participants who received drop-shipped frozen meals, although these differences were not statistically significant. While this pilot trial was not adequately powered to detect effects that were as large as the observed differences at a 5% nominal level, the results suggest that there may be lower nursing home placement among individuals with self- or proxy-reported dementia who received daily-delivered meals with socialization and a safety check compared with individuals who received drop-shipped frozen meals.

We enrolled 243 of 325 individuals with self- or proxy-reported dementia on waiting lists for MOW. The 243 participants differed by race and ethnicity and program from those not enrolled, suggesting that future work using this approach may need to consider the generalizability of enrollees compared with the eligible population. These findings provide useful estimates for the number of people who meet eligibility criteria we would need to identify on waiting lists at local MOW programs to enroll the required number of participants in a well-powered trial. In addition, the variation by program addressed the importance of understanding practices at each site, which relates to different methods of using waiting lists and contacting people to initiate service. We also found that we were able to link all but 16 participants with their CMS data using the information routinely collected by MOW programs. This information and our approach to linking the unlinked individuals may be helpful to other pragmatic trialists who are collecting identifiable information to link with administrative data.

The results from this pilot study, which took place during 2021, must be interpreted considering the COVID-19 pandemic and implications it had on our study. First, MOW programs implemented procedures in response to the pandemic that reduced the frequency and kind of interactions that drivers had with clients, 1 of the key differentiators between the 2 study arms. For example, 1 program implemented social distancing requirements in which a driver could not enter a home but rather had to hang a bag with the food on the door, knock, and step away. Another program reduced delivery of daily-delivered meals to twice per week during the pandemic because of a decline in volunteer drivers. The reduction in the number of daily deliveries meant some meals were delivered chilled and may have needed preparation (eg, reheating). While reheating the chilled meals may have been simpler than preparing the frozen meals, the benefit of the ready-to-eat meal characterized by the daily meal delivery model may have been reduced for some. Given these COVID-19–related changes to the ways in which meals were delivered for the daily-delivered meals arm, it is likely that the effect of daily-delivered meals was attenuated in this pilot study. In addition, our measure of participant fidelity (ie, receiving meals for the full 6-month study period) could have been impacted by the pandemic and may have been specific to the population with self-reported dementia. Based on the National Survey of Older Americans Act Participants, more than 80% of its respondents received meals for 6 or more months.^6^ We anticipated a similar rate of participants completing the 6 months with meals; however, only two-thirds of participants (68.8% in the daily-delivered meals arm and 62.6% in the drop-shipped frozen meals arm) were still receiving meals at the end of the 6-month study period. This information will be important to future work in regard to budgeting for interventions and in understanding how fidelity or dose of meals relates to the outcomes observed.

### Limitations

Consistent with the pragmatic design, this study relied on self-reported and proxy-reported information to identify individuals with dementia. Using information that programs typically collect, this pilot is more generalizable than a study that requires a confirmed diagnosis. Future research should validate this self-reported information or assess dementia severity more thoroughly to further nuance recommendations of meal-delivery effectiveness. Another possible limitation is that more participants in the arm assigned to drop-shipped frozen meals lived alone compared with those assigned to daily-delivered meals. We adjusted for living arrangements in the survival model to address this imbalance, but there may still be the possibility of bias, particularly given the challenges that participants expressed for transporting, storing, and reheating frozen meals that were likely exacerbated if living alone.^21,35^ Future trials should consider stratifying on this variable to ensure balance between arms given the relationship between living arrangement and nursing home placement.^35^ Additionally, we were unable to deterministically link 16 participants to CMS data, which may induce bias. However, proportions of unlinked individuals in each arm were relatively similar, and we relied on an established probabilistic linkage algorithm to link these individuals while propagating possible errors in the linkage.^8,29^ Finally, conducting a larger-scale trial that examines not only the effectiveness of the meal-delivery models but also the impact of variability in implementation (eg, delivery processes, paid vs volunteer drivers, meal frequency, and level of social interaction) would further inform guidance for programs and decision-makers.

## Conclusions

The findings of this pilot clinical trial demonstrated the feasibility of conducting an embedded pragmatic trial within MOW programs, enrolling older adults with dementia, monitoring their fidelity, linking data, and measuring participants’ outcomes. Although there was lower nursing home placement among participants receiving daily-delivered meals, this result was not significant; however, it does suggest that proceeding to a full-scale, conclusive, pragmatic randomized clinical trial is warranted to inform decision-making about the provision of home-delivered meals for homebound older adults living with dementia. This information is important as health systems, health care professionals, medical institutions, and health plans are increasingly providing meals to patients, a large and growing number of whom are living with dementia.

## Notes

Supplement 1.
Trial Protocol

Supplement 2. **eTable.** Baseline Characteristics of Analyzed Participants, by Study Arm

Supplement 3.
Data Sharing Statement
