# Supplementary material for: Home-Delivered Meals and Nursing Home Placement Among People With Self-Reported Dementia: A Pilot Pragmatic Clinical Trial
Source: JAMA Netw Open. 2023 Dec 20;6(12):e2347195. doi: 10.1001/jamanetworkopen.2023.47195 (PMC10733798; doi:10.1001/jamanetworkopen.2023.47195)
Supplement: Supplement 1. — Trial Protocol [file jamanetwopen-e2347195-s001.pdf]

Application for Expedited / Full Board IRB Review

PART 1: Name(s) and Contact Information.

Protocol Title: Home-delivered meals for persons with dementia: Which model delays nursing home placement?

Principal Investigator: Kali Thomas, PhD
For more information on who may serve as a PI, see Brown's guidance and PI Eligibility Policy.

Department: Health Services, Policy and Practice

Phone number: 401-863-9036

Email address: kali_thomas@brown.edu

Is this a graduate student project?* ☐Yes ☒ No

If student PI, please provide the following:

Advisor:

Department:

Phone number:

Email address:

Is this an undergraduate student project?* ☐Yes ☒No Ifyes,

name of undergraduate student:

PART 2: Education Affirmation.

Human Subjects CITI training is complete (PI, advisor (if student PI)): ☒ Yes ☐No

Good Clinical Practice (GCP) training is complete (clinical trials only): ☒ Yes ☐No ☐N/A

HIPAA training is complete (if using PHI): ☒ Yes ☐No ☐N/A

PART 3: Collaboration Information.

Are there multiple sites involved with this study? ☒Yes ☐No

If yes, list the site(s) involved: Meals on Wheels of America

- If "yes," review the [Application for IRB Authorization Agreement]

PART 4: Funding Information.

Funding Source(s):

- If externally funded, provide the following:
  Sponsor: National Institute on Aging

Project title: Home-delivered meals for persons with dementia: Which model delays nursing home placement?

Grant / Contract #: R61AG070170

- If funded by a specific Brown program (e.g., Mellon Mays Fellowship, Royce Fellowship, UTRA, OVPR Seed funds, etc.) please specify**:**
- If there is no funding for the study, write "Brown"

PART I. HUMAN SUBJECTS RESEARCH SCREENING

**Expedited / Full Board studies must meet the federal definition of "Human Subjects Research." Answer the following questions to determine if your proposed study meets the federal definitions of both "Research" and "Human subjects."**	
☒Yes ☐No	Is this study a systematic investigation?
☒Yes ☐No	Is the *primary design intent* of this study to contribute to generalizable knowledge?
☒Yes ☐No	Is the information being obtained *about* living individuals?
☒Yes ☐No	Will you collect information through some type of intervention or interaction? **OR**<br>Will you have access to individually identifiable information? **OR**<br>Will you have access to private information?
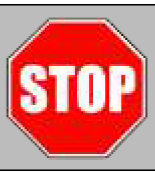	If you answered "no" to any of the above questions, your study does not meet the definition of "Human Subjects Research." You are not required to submit an Application for IRB review to the Brown HRPP.

PART II. RISK ASSESSMENT & EXPEDITED ELIGIBILITY SCREENER

**1. Minimal Risk means that the probability and magnitude of harm or discomfort anticipated in the research are not greater in and of themselves than those ordinarily encountered in daily life or during the performance of routine physical or psychological examination or tests.**<br><br>**Using this definition, do you believe this research presents:**	
☒Greater than minimal risk (**Full Board**)	Briefly justify this selection (and proceed to Part III):<br><br>Grace Ayers at the HRPP indicated our request for a waiver of informed consent for randomization and our receipt of administrative data means this project requires full board review

Brown University IRB Original Approval: 12/10/2020
Brown University IRB Amendment Approval 02/25/2021
Brown University IRB Amendment Approval 05/11/2021
Brown University IRB Amendment Approval 08/19/2021

Brown University IRB Amendment Approval 10/06/2021

☐ No greater than minimal risk (**Expedited**)	Briefly justify this selection (and proceed to Question 2):<br>Click or tap here to enter text.
**2. Below are Research Categories *eligible* for Expedited Review. Select one or more of the categories that are applicable to your proposed research, if any.**	
☐ Category 1	Clinical studies of drugs and medical devices only when condition (a) or (b) is met (please select one):<br>☐ (a) research on drugs for which an IND application is not required. (Note: Research on marketed drugs that significantly increases the risks or decreases the acceptability of the risks associated with the use of the product is not eligible for expedited review); OR<br><br>☐ (b) research on medical devices for which (i) an IDE exemption application is not required; or (ii) the medical device is cleared/approved for marketing and the medical device is being used in accordance with its cleared/approved labeling.
☐ Category 2	Collection of blood samples by finger stick, heel stick, ear stick, or venipuncture as follows:<br>☐ (a) from healthy, non-pregnant adults who weigh at least 110 pounds. For these participants, the amounts drawn must not exceed 550 ml in an 8-week period and collection may not occur more frequently than 2 times per week; OR<br><br>☐ (b) from other adults and children, considering the age, weight, and health of the participants, the collection procedure, the amount of blood to be collected, and the frequency with which it will be collected. For these participants, the amount drawn may not exceed the lesser of 50 ml or 3 ml per kg in an8-week period and collection may not occur more frequently than 2 times per week.
☐ Category 3	Prospective collection of biological specimens for research purposes by noninvasive means. Examples may include:<br>(a) hair and nail clippings in a non-disfiguring manner;<br>(b) deciduous teeth at time of exfoliation or if routine patient care indicates a need for extraction;<br>(c) permanent teeth if routine patient care indicated a need for extraction;<br>(d) excreta and external secretions (including sweat);<br>(e) uncannulated saliva collected either in an unstimulated fashion or stimulated by chewing gum base or wax or by applying a dilute citric solution to the tongue;<br>(f) placenta removal at delivery;<br>(g) amniotic fluid obtained at the time of rupture of the membrane prior to or during labor;<br>(h) supra- and subgingival dental plaque and calculus, provided the collection procedure is not more invasive than routing prophylactic scaling of the teeth and the process is accomplished in accordance with accepted prophylactic techniques;<br>(i) mucosal and skin cells collected by buccal scraping or swab, skin swab, or mouth washings;<br>(j) sputum collected after saline mist nebulization.
☐ Category 4	Collection of data through noninvasive procedures (not involving general anesthesia or sedation) routinely employed in clinical practice, excluding procedures involving x-rays or microwaves. Where medical devices are employed, they must be cleared/approved for marketing. (Studies intended to evaluate the safety and effectiveness of the medical device are not generally eligible for expedited review, including studies of cleared medical devices for new indications.)<br>Examples may include:<br>(a) physical sensors that are applied either to the surface of the body or at a distance and do not involve input of significant amounts of energy into the subject or an invasion of the subject's privacy;<br>(b) weighing or testing sensory acuity;<br>(c) magnetic resonance imaging;<br>(d) electrocardiography, electroencephalography, thermography, detection of naturally occurring radioactivity, electroretinography, ultrasound, diagnostic infrared imaging, doppler blood flow, and echocardiography;

Brown University IRB Original Approval: 12/10/2020
Brown University IRB Amendment Approval 02/25/2021
Brown University IRB Amendment Approval 05/11/2021
Brown University IRB Amendment Approval 08/19/2021
Brown University IRB Amendment Approval 10/06/2021

	(e) moderate exercise, muscular strength testing, body composition assessment, and flexibility testing where appropriate given the age, weight, and health of the individual.
☒Category 5	Research involving materials (data, documents, records, or specimens) that have been collected, or will be collected solely for non-research purposes (such as medical treatment or diagnosis). NOTE: Some research in this category may be Exempt. Review the categories for Exemption before selecting this option.
☐Category 6	Collection of data from voice, video, digital, or image recordings made for research purposes.
☒Category 7	Research on individual or group characteristics or behavior (including, but not limited to, research on perception, cognition, motivation, identity, language, communication, cultural beliefs or practices, and social behavior) or research employing survey, interview, oral history, focus group, program evaluation, human factors evaluation, or quality assurance methodologies. NOTE: Some research in this category may be Exempt. Review the categories for Exemption before selecting this option.

Brown University IRB Original Approval: 12/10/2020
Brown University IRB Amendment Approval 02/25/2021
Brown University IRB Amendment Approval 05/11/2021
Brown University IRB Amendment Approval 08/19/2021

Brown University IRB Amendment Approval 10/06/2021

PART III. RESEARCH DESIGN & METHODS

THE BLUE TEXT IN THE FOLLOWING SECTIONS IS A GUIDE TO ENSURE ALL RELEVANT INFORMATION IS INCLUDED IN YOUR APPLICATION. YOU MAY DELETE THE BLUE TEXT BEFORE SUBMISSION

1. **Introduction and Background.** *In reviewing the protocol, the IRB must consider the rationale for the study and the importance of the knowledge that may reasonably be expected to result.*

   Food insecurity among older adults is a critical health and healthcare issue with implications for families, the healthcare system, and society. In 2017, an estimated 5.5 million older adults (7.7%) in the United States were food insecure. Food insecurity, defined as a lack of consistent access to enough food for an active, healthy life, disproportionately affects older adults who are racial or ethnic minorities, those with lower incomes, those who are disabled, and those living in non-metropolitan areas. Food is a basic human need, and among many food- insecure older adults, the need for food competes with other basic necessities such as medication, housing, utilities, and transportation. While food-insecure older adults sometimes have enough money to purchase food, they may not have the resources to access or prepare food due to lack of transportation, functional limitations, or health problems.

   Individuals with Alzheimer's disease and related dementias (ADRD) are at increased risk of food insecurity. Many individuals with ADRD living in the community are characterized as being low income, with close to one- quarter living under the Federal Poverty Level and almost one-third enrolled in Medicaid. In early stages of ADRD, when the ability to perform complex tasks is declining, difficulties in shopping, preparing meals, and eating may regularly arise, especially among persons living alone, which may mark the entry into a vicious circle of food insecurity and health instability. Estimates of the percent of persons with dementia (PWD) living in the community who need assistance shopping for and preparing meals range from 50% to 90%. Caregivers play a central role in recognizing these difficulties and providing support. However, between 25% and 30% of PWD in the community live alone, and approximately one quarter of PWD who live alone have limited social support and report not visiting with family or friends in the last month. Therefore, PWD in the community, particularly those who live alone, are at increased risk of food insecurity.

   Among older adults, food insecurity is associated with poor health status and health outcomes, and accounts for an estimated $130 billion annually in healthcare expenses. Previous research suggests that addressing food insecurity is associated with reduced odds of hospitalization, emergency department use, and nursing home admission, including shorter lengths of nursing home stays. In addition, evidence, much of which our team has helped generate, continues to suggest that home-delivered meals, specifically, have an impact on the health and healthcare utilization of a highly vulnerable population of homebound older adults experiencing food insecurity. Recognizing the link between food insecurity, health, and healthcare utilization, healthcare entities (e.g., payers, systems, providers) are increasingly interested in addressing food insecurity with home-delivered meals for older patients.

   While home-delivered meals have traditionally been provided daily to clients' homes by a volunteer or paid driver, mailed frozen meals have emerged in recent years as a lower-cost

Brown University IRB Original Approval: 12/10/2020
Brown University IRB Amendment Approval 02/25/2021
Brown University IRB Amendment Approval 05/11/2021
Brown University IRB Amendment Approval 08/19/2021
Brown University IRB Amendment Approval 10/06/2021

alternative. In this model, participants are provided one to two weeks' worth of meals in one bulk delivery via postal courier. While these meals have the same nutritional standards as daily-delivered meals (⅓ of older adults' Dietary Reference Intake), there is limited evidence on how satisfaction with, and the benefit of, these meals may differ from the traditional daily- delivered meal. Our pilot research suggests that clients who receive daily-delivered meals (including socialization and a wellness check) have lower rates of loneliness and falls than clients who receive frozen, weekly delivered meals. These findings are particularly salient given the relationship between these two measures and older adults' health and healthcare utilization. **However, overall differences in health and healthcare utilization, including the ability for meals to enable PWD to remain in the community, attributable to the two meal delivery methods remain unknown.**

The healthcare sector has a growing interest and motivation to address food insecurity in order to achieve better patient health outcomes and reduce costs. Increasingly, providers and health systems are addressing food insecurity and its impact on health through the direct provision of meals. Medicare Advantage (MA) plans, which currently enroll over one-third of all Medicare beneficiaries, were given new flexibility to address chronically ill enrollees' social needs, including enrollees with ADRD, through the, "Creating High-Quality Results and Outcomes Necessary to Improve Chronic (CHRONIC) Care Act," of 2018. Interviews that our team conducted with MA plans across the country indicate that they are examining new opportunities to meet the needs of chronically ill enrollees through the provision of home-delivered meals in response to CHRONIC. In fact, our preliminary work using MA plan benefit data suggests that 1,979 MA plans (47.4%) are offering a meal benefit to their 9.4+ million enrollees in 2020. In addition, many local communities offer meal and nutrition services to eligible individuals through Medicaid waivers or other government-funded programs. The Centers for Medicare and Medicaid Services (CMS) through their Accountable Health Communities Model are testing how identifying and addressing patients' social needs, like food insecurity, impacts healthcare costs and reduces utilization. Despite the increased focus on addressing social needs like food insecurity through home-delivered meals, healthcare entities have little information guiding them about what mode of meal delivery (meals delivered by the MOW program multiple times a week with socialization vs. frozen, drop-shipped meals) promotes community independence among PWD and benefits their caregivers.

It is particularly important to examine the differences in experiences between the two modes of meal delivery and how they impact community residence among PWD, who comprise approximately 30% of Meals on Wheels (MOW) clients. There are several reasons why examining differences in the two modes of meal delivery among PWD is of great interest. For example, frozen, drop-shipped meals require the client to transport the heavy box of frozen meals to the kitchen, put them in the freezer, microwave them, open the meal containers, etc., all of which may be particularly challenging for PWD. However, the hot/chilled meals delivered multiple times a week by the MOW program are brought directly to the client. Interviews we have conducted with MOW drivers found that it is common for drivers to bring the meal to the kitchen/table and set up the meal for the client (e.g., open containers). Further, PWD may benefit greatly from physical check-ins by a meal delivery driver because PWD may not always accurately self- report or request help if thereare issues. Better understanding of the process of meal delivery and its impacts on the lives of PWD can improve services to meet clients' needs and provide evidence to guide decision- making around what type of meals to provide and to whom.

Brown University IRB Original Approval: 12/10/2020
Brown University IRB Amendment Approval 02/25/2021
Brown University IRB Amendment Approval 05/11/2021
Brown University IRB Amendment Approval 08/19/2021
Brown University IRB Amendment Approval 10/06/2021

The proposed research will address key knowledge gaps identified by a multitude of stakeholders. Two recently released reports by the National Academies of Science, Engineering, and Medicine called for rigorous research to understand effective and efficient ways for the healthcare sector to reduce food insecurity and other health-related social needs. Our proposed research addresses the need to identify, "how interventions affect health, for which patients, via what mechanisms, and in what contexts," put forward by the committee, and works toward their recommended goal to "Fund, Conduct, and Translate Research and Evaluation on the Effectiveness and Implementation of Social Care Practices in Health Care Settings." Also, this work meets the needs of other stakeholders, including MA plans and Medicaid managed care organizations, both of which indicated the need for evidence around program effectiveness to guide decision-making. **Our proposed research is significant because it will provide evidence to guide stakeholders' decisions and ultimately improve PWDs' health outcomes and quality of life.**

Specific Aims and Study Objectives. *The IRB must evaluate the objectives of the research in order to determine whether the risks to participants are reasonable in relation to the importance of the knowledge that may be gained.*

The Specific Aim of the R61 is to:

1. **Test and validate procedures to recruit individuals with ADRD and evaluate their outcomes in partnership with Meals on Wheels programs.** The goal of this aim is to test and validate the vital elements and procedures for conducting a pragmatic effectiveness trial: specifically, 1) enrolling persons with ADRD on MOW programs' waiting lists to receive one of the two types of meals; 2) recruiting a subsample of participants and caregivers to participate in telephone interviews; 3) extracting and transferring program data to Brown University; 4) linking participant data with Medicare and nursing home assessment data.

If your study ONLY involves the use of identifiable secondary data / biospecimens, including coded data from which you may be able to ascertain participant identity, skip to PART V. Otherwise, please continue to next page.

2. **Materials, Methods and Analysis.** *The study design, methods and procedures must be adequately described in order for the IRB to understand all activities in which human subjects will participate. The IRB must also be able to differentiate those procedures that are performed for research purposes from those that are performed for routine care or evaluation.*

This application is a pragmatic randomized controlled trial comparing outcomes among two approaches for delivering meals to food insecure older adults with Alzheimer's disease and related dementias (ADRD). Research procedures include assignment to one of the two predominant modes of meal delivery in standard practice, primary data collection, and secondary data analysis.

We propose a two-arm, parallel, randomized control trial. The initial phase of this project will be a pilot phase with one aim.

Brown University IRB Original Approval: 12/10/2020
Brown University IRB Amendment Approval 02/25/2021
Brown University IRB Amendment Approval 05/11/2021
Brown University IRB Amendment Approval 08/19/2021
Brown University IRB Amendment Approval 10/06/2021

Aim 1. Test and validate procedures to recruit participants with ADRD and evaluate their outcomes in partnership with MOW programs. The goal of this aim is to test and validate the <u>vital elements and procedures for conducting the pragmatic effectiveness trial</u>: specifically, 1) enrolling persons with ADRD on MOW programs' waiting lists to receive one of the two types of meals; 2) recruiting a subsample of participants and caregivers to participate in telephone interviews; 3) extracting and transferring program data to Brown University; 4) linking participant data with Medicare and nursing home assessment data

Enrolling Persons with ADRD on MOW Programs' Waiting Lists to Receive One of the Two Types of Meals. We will work with programs during this pilot research award to test the procedures for enrolling persons with ADRD who are on programs' waiting lists to receive one of the two types of meals that are currently provided by home-delivered meals programs (i.e., meals delivered multiple times a week by the MOW programs versus frozen, drop-shipped meals) As a note: we have been informed by the MOW programs that the frequency of the daily delivered meals has been shifted to multiple times a week due to the COVID-19 pandemic. These meal recipients are still receiving the same number of meals, but delivery may occur multiple times a week instead of daily. Daily meal delivery may resume when the MOW programs are able to safely do so. Both types of meals are regularly provided to individuals with dementia. To do this, the MOW program will identify individuals who are already on their waiting list and during the initial intake indicated that a doctor or other healthcare professional had indicated that they suffer from memory loss, cognitive impairment, any type of dementia, or Alzheimer's disease (this could be self-reported or by the person making the referral). This information is available in the programs' intake records and therefore will not require any additional data collection or screening procedures. Brown University will randomize those individuals into one of the two groups (meals delivered multiple times a week by the MOW programs or frozen meals) and notify the MOW program. The MOW programs will reach out to individuals and offer them the delivery type. If an individual agrees to come off of the waiting list, they will begin receiving meals. If not, they will remain on the waiting list (i.e., there is no penalty for declining). The MOW program will inform theindividual that their decision to come off the waiting list will not influence their place on the waitinglist -- that is, if (for whatever reason) decide not to continue to receive their randomly-assigned mealtype, the individual will be returned to the waiting list and will not lose their spot. We are requestinga waiver of informed consent for this portion of the study, as we believe this study meets the five criteria for a waiver as described in 45 CFR §46.116 f 3 (i-v) and detailed below. For those who have agreed to receiving one of the two types of meals, MOW will send client intake information that they regularly collect (e.g., name, address, phone number, date of birth, last four of SSN, race, gender, living arrangement) to Brown University through secure methods. Brown University researchers will randomize the participants and will communicate assignments back to the MOW programs. Upon receiving the assignment, the MOW program will begin service for clients and continue to serve the assigned meals for six months. Following the six-month study period, individuals may continue to receive their assigned meals or roll onto the MOW programs' usual service (i.e., meals delivered multiple times a week by the MOW program). Importantly, the three programs who are participating have agreed to continue to serve meals to interested individuals after the study period ends.

Recruiting a Subsample of Participants to Take Part in Telephone Interviews. To obtain rich information about the context, implementation, and mechanisms of impact associated with receiving meals, a subset of PWD who accepted the intervention will be interviewed by Brown researchers. To do this, the MOW program will send an invitation letter and informed consent sheet to participants from the first two programs (VNA Texas and Neighborly) approximately one month after the first meal delivery. This letter will include an invitation to participate in an in-depth, semi-structured pilot phone interview with Brown University researchers. Interested individuals will "opt-in" to participate by contacting the research team with the information included in the initial invitation research team will also send participants from the third site (MOW-SA) an opt-out letter. This opt-

Brown University IRB Original Approval 12/15/2020
Brown University IRB Amendment Approval 02/25/2021
Brown University IRB Amendment Approval 05/11/2021
Brown University IRB Amendment Approval 08/19/2021
Brown University IRB Amendment Approval 10/06/2021

letter. Additionally, if any of the participants from the first two programs (VNA Texas and Neighborly) do not respond to the initial letter, the Brown University research team will send a second letter, reminding participants about the opportunity to participate in a phone interview. To test out a different recruitment protocol in hopes of increasing participation, the Brown University

research team will also send participants from the third site (MOW-SA) an opt-out letter. This opt-

out letter will

describe the study and will let them know that a member from the research team will contact them unless they “opt-out” of the study.
Within this introductory letter, we will provide our contact information and will also include a refusal to participate postcard that participants will be asked to complete and return if they do not wish to participate in the study. For participants for whom we do not receive a refusal postcard, telephone call, or email indicating that they are not interested in participating, we will make no more than 3 attempts to contact them between the hours of 10am and 7pm, in their representative time zones.
The Brown University research team will call the interested participants, complete an informed consent process over the telephone, and conduct the interview. Pilot interview questions will be guided by the Medical Research Council’s Process Evaluation Framework. We will include questions focused on implementation (e.g., participants’ interaction, if any, with the driver/mail carrier who delivers their meals), mechanisms of impact (e.g., participants’ experiences receiving, preparing, and eating meals); and outcomes (e.g., participants’ satisfaction with the meal services). We will also administer the Telephone Interview for Cognitive Status (TICS-M) to assess participants’ cognitive abilities. We will test our procedures for recruiting and conducting the interviews with 2-10 participants at each program. While we initially proposed to conduct in-person interviews, because of COVID-19, we will pursue telephone interviews and should conditions change, we will seek IRB approval to conduct in-home interviews. Nonetheless, telephone interviewsare efficient, cost-saving, and allow for the greatest amount of privacy for participants.

Recruiting a Sample of Caregivers to Take Part in Telephone Interviews. We will conduct semi-structured telephone interviews with a subset of caregivers of PWD who accepted the intervention to better understand the process of receiving meals and potential mechanisms behind outcomes observed. We will test our procedures and pilot the interview guide with 2-10 caregivers at each program with separate methods at the three programs (i.e., VNA Texas, Neighborly Senior Services, and MOW-San Antonio). In the first site, VNA Texas, MOW programs will mail the invitation letter to the emergency contact listed in the clients’ records approximately three months after the participant begins receiving meals. Interested caregivers will “opt-in” to the study by contacting Brown University researchers who will describe the study, obtain verbal consent to participate over the telephone, and conduct the telephone interview. In the second and third sites, Neighborly Senior Services and MOW-San Antonio, researchers will ask participants who are interviewed if they would share the contact information of an informal caregiver. Brown University researchers will mail a welcome letter approximately three months after the participant begins receiving meals, which will describe the study and that a member from the research team will contact them to schedule the interview unless they “opt-out” of the study. Within this introductory letter, we will include a refusal to participate postcard that caregivers will be asked to complete and return if they do not wish to participate in the study. For caregivers from whom we do not receive a refusal postcard, telephone call, or email indicating that they are not interested in participating, we make no more than 3 attempts to contact them between the hours of 10am and 7pm, in their representative time zones. The Brown University research team will be responsible for calling caregivers, conducting an informed consent process over the telephone, and completing the interview. These interviews, also guided by the Medical Research Council’s Process Evaluation Framework, will focus on caregivers’ experiences and caregivers' perceptions of participants' experiences receiving meals, their interactions, if any, with the person who delivers their meals (i.e., driver or mail carrier), how the meal service may have impacted their lives (e.g., responsibilities, stress, burden), and their satisfaction with the meal services. We have chosen to conduct telephone interviews because they are an efficient, cost-saving, and effective way to ensure a diverse set of

participants and allow for the greatest amount of privacy and convenience of caregivers.

Extracting and Transferring Program Data to Brown University. During this pilot study, in addition to developing our data sharing platform and testing data transfer with our pilot sites, we will test data transfers at least twice per participating program. We will ask MOW programs to shareenrollment and programmatic data that is regularly collected from current clients who have a self- reported diagnosis of dementia/cognitive impairment. This is information that is routinely collected and available in MOW programs' records. While we have confidence in our ability to obtain and link MOW program data to CMS data given our work in a previously funded study, the goal of this activity is to test the data sharing platform and refine instructions provided to programs. Brown staffhave considerable experience receiving data directly from a variety of sources (e.g., nursing home corporations) in various formats for pragmatic trials. Our IT director will work with MOW staff around data transfer options. Options will include transfer by physical media or through electronic submission. By either method, data will be encrypted to at least AES-256 standards prior to transmission. Passwords will be transmitted out of band, either verbally or via email, or an SMS textmessage.

Linking Participant Data with Medicare and Nursing Home Assessment Data. Participant data (i.e., first and last name, phone number, date of birth, race/ethnicity, gender, address, last four of SSN) will be shared with Brown investigators by MOW programs via secure methods and downloaded to a secure Brown server. Participant information will be shared securely with HealthAPT to obtain identifiers (i.e., bene_id) to link across the Medicare enrollment record, nursing home MDS, and claims. Our team has access to 100% national data through our Program Project and the PI and co-investigators on this proposal are intimately familiar with the data and adept in linking files in this way. We will assess the match rate with these unique identifiers to determine the best combination of data needed for linkage in the full trial. Further, we will use the current enrollment data to estimate the probabilities of linkages for our non-matches using our previously derived algorithm that we used to link MOW client data with Medicare enrollment records *without* unique identifiers. Finally, we will conduct initial exploratory analyses to determine if nursing home placement differs between the two groups of participants in the pilot.

3. **Participant Population.** *In order to approve research, the IRB must determine that the selection of participants is equitable and reasonably related to the purpose and aims of the research. The IRB must also consider whether adequate safeguards are in place to minimize any risks that are unique to vulnerable populations. To make this determination, the IRB must review all methods and materials used to contact and recruit potential participants, including letters, flyers, emails, etc.*

During this pilot phase, we will work with three programs who will deliver meals to 235 participants. This willallow us to refine the process for enrolling and randomizing PWD to receive meals. To do this, we will pilot the procedures with three MOW programs: Neighborly Senior Services in Clearwater, Florida; VNA Texas inDallas, Texas; and Meals on Wheels – San Antonio in San Antonio, Texas.

The inclusion criteria for receiving meals include being on the waiting list, 66 years of age or older (to allow for Medicare enrollment and a one-year baseline lookback) and self-reporting a diagnosis of dementia or cognitive impairment. Age and self-reported diagnosis come from the MOW programs' intake records and therefore <u>no additional screening or data collection is required</u> for receiving meals (i.e., enrollment in the study).

Brown University IRB Original Approval: 12/10/2020
Brown University IRB Amendment Approval 02/25/2021
Brown University IRB Amendment Approval 05/11/2021
Brown University IRB Amendment Approval 08/19/2021
Brown University IRB Amendment Approval 10/06/2021

There is no requirement that participants be English-speaking in order to receive meals. MOW programs during their usual intake procedures confirm that individuals have working refrigerators, microwaves, etc. necessary to store and heat the food. Both participating programs, like many MOWA-member programs, have procedures in place for supplying this equipment if it is determined that a client is in need. MOW programs may have a limited ability to assess individuals' cognitive abilities to store, heat, or prepare food. Furthermore, there are many MOW programs in which frozen meals are the only option - these programs do not withhold meals for individuals with diminished cognitive abilities.

The goal is to test, on a very small scale, procedures that we will use later on a not-yet-funded R33. The R33 is designed to be pragmatic and will be scaled up to the anticipated goal sample of 2300. We selected 235 participants for this pilot because we want to ensure we will reach the goal we had set out in the short time period of the pilot project.

For the interviews, we will recruit a subsample of 2-10 persons with dementia receiving meals and 2-10 caregivers at each program to pilot the procedures and interview guides. For persons with dementia, inclusion criteria consists of receiving meals, being English-speaking, and having capacity to consent at the time of the consent discussion and interview. Members of the research team have previous experience conducting research with individuals with impaired decision-making capacity. Although participants can end the interview at any time, the research team members conducting the interview will continue the interview even if the reliability of the information provided is suspected to be incomplete or not pertinent to the question asked. As a pilot of this procedure for the larger trial, the quality of the data reported by participants with dementia is of interest to the study team.

The research team members conducting the interviews (i.e., Dr. Gadbois, Dr. Shield, Dr. McAuliff) will identify and make note when the content provided by the participant does not contain information pertinent to the questions being asked. As long as the person with dementia participating in the interview is not distressed and wants to continue, then the interview will be completed as planned. In our experience, a participant may understand a question later in the interview and be able to provide relevant information even in cases where they had a less reliable response to an earlier item / interview question. However, if the participant is not able to meaningfully respond to a series of questions such that a lack of data integrity is clear the interviewer can end the interview early. If that occurs, the research team member will end the interview by thanking the participants for their contribution (e.g., “Thank you for sharing that with me. That is all of the questions that I have for you today. I really appreciate you taking this phone call. Is there anything else that you want me to know?”). It is not necessary to tell participants the interview is being discontinued, in all cases empathy and dignity will be prioritized.

For caregivers, interview inclusion criteria consist of being an informal caregiver of a PWD who is receiving home-delivered meals and English-speaking.

4. **Recruitment Methods**

Randomization to Meal Group: MOW program staff will identify individuals on their existing waiting lists who are age 66+ years and have a self-reported memory impairment or diagnosis of dementia. This information is available in the programs’ intake records and therefore will not require any additional data collection or screening procedures.

Brown University will randomize individuals into one of the two groups (meals delivered multiple times a week by the MOW program or frozen meals) and notify the MOW program. The MOW programs will reach out to individuals and offer them the delivery type, allowing them to come off the waiting list. However, participation is voluntary and if individuals do not want to come off of the waiting list, they willretain their place on the waiting list. The MOW program will inform the individual that their decision to come off the waitinglist will not influence their place on the waiting list - meaning, if they decide not to continue to receive their assigned meal type, they will be returned to the waiting list and will not lose their spot.

The average time that clients spend on a waiting list at these three programs is between 3-5 months; individuals who decline to participate or who decide to leave the study and return to the waiting list will keep their original place on the waiting list (i.e., their accrued waiting time). The client information will be shared with the research team at Brown University who will be responsible for randomizing the client to meals delivered multiple times a week by the MOW program or bi-weekly, drop-shipped meals, and communicatingthe assignment back to the MOW program. We will consult with participating programs, our expert ethicist consultant, Emily Largent, PhD, RN, JD, and the Brown IRB on the process (e.g., general barriers to enrollment, reasons for refusal). Based on this feedback, we will make refinements to the implementation strategy and training materials for the larger trial.

Brown has an established collaborative relationship with MOWA, which represents over 5000 home-delivered meals programs. In partnership with MOWA, we have already recruited 2 MOWA member programs to participate in this pilot (please see letter of support from MOWA). MOWA's member programs have agreed to collectively enroll clients with ADRD, to devote staff to implement the intervention, and to work with the Brown research team to develop and execute secure data transfer protocols. MOWA has ensured their support of recruiting additional programs to participate, if needed.

Telephone Interviews with Participants: We will recruit 2-10 participants receiving meals at each program to pilot the telephone interview protocol. The MOW program will send an invitation letter and informed consent sheet to the first two MOW programs (VNA Texas and Neighborly) approximately one month after the first meal delivery. This letter will include an invitation to participate in an in-depth, semi-structured pilot phone interview with Brown University researchers. The invitation letter will describe the study and include the contact information for Brown University researchers who the participant can call to express interest in participating. Interested individuals will "opt-in" to participate by contacting the research team with the information included in the initial invitation letter. If any of the participants from the first two programs (VNA Texas and Neighborly) do not respond to the initial letter, the Brown University research team will send a second letter, reminding participants about the opportunity to participate in a phone interview. To test out a different recruitment protocol in hopes of increasing participation, the Brown University research team will also send participants from the third site (MOW-SA) an opt-out letter. This opt-out letter will describe the study and will let them know that a member from the research team will contact them unless they "opt-out" of the study.

Within this introductory opt-out letter, we will provide our contact information and will also include a refusal to participate postcard that participants will be asked to complete and return if they do not wish to participate in the study. For participants for whom we do not receive a refusal postcard, telephone call, or email indicating that they are not interested in participating, we will make no more than 3 attempts to contact them between the hours of 10am and 7pm, in their representative time zones.

A trained Brown University research team member will call the interested participants, screen participants, complete an informed consent process, and complete the interview all over the telephone. The interviews will be scheduled at a time of each participant's choosing to ensure privacy and convenience. Interviews will be designed to collect information about participants' experiences receiving, preparing, and eating meals; their interaction, if any, with the person who delivers their meals (i.e., driver or mail carrier); and their satisfaction with the meal services. Interviews will be tailored around the needs of older adults with AD/ADRD. If the interviewee loses his or her train of thought, the interviewer will repeat the last topic or sentence. If participants are too impaired to correctly or effectively answer specific questions, the interviewer will use examples rather than open-ended questions.

Brown University IRB Original Approval: 12/10/2020
Brown University IRB Amendment Approval 02/25/2021
Brown University IRB Amendment Approval 05/11/2021
Brown University IRB Amendment Approval 08/19/2021
Brown University IRB Amendment Approval 10/06/2021

In addition to the interview questions, we will conduct a brief cognitive assessment (i.e., Telephone Interview of Cognitive Status (TICS-M)) to estimate the participant's level of cognitive impairment. The TICS-M will be conducted after the interview questions, and the score will be used to understand how experiences may differ for clients with differing levels of cognitive impairment. There is no cut-off score on the TICS-M for conducting the interview and it will not affect eligibility. Scores on cognitive screeners, like the TICS-M, do not consistently predict capacity to consent to research and clinical interview is considered the gold standard for capacity assessment. That is why we are using a study-specific capacity to consent assessment and not the score on the TICS-M.

Telephone Interviews with Caregivers: We will test our procedures and pilot the interview guide with 2-10 caregivers at each program using two different methods at the three programs (one program will use one method, the other two programs will use a different method). In the first site, MOW programs will mail the invitation letter to the emergency contact listed in the clients' records approximately three months after the participant begins receiving emails. Interested caregivers will "opt-in" to the study by contacting Brown University researchers. A trained member of the Brown University research team will describe the study, obtain verbal consent to participate over the telephone, and conduct the telephone interview. In the second and third sites, researchers will ask participants who are interviewed if they would share the contact information of an informal caregiver. Brown University researchers will mail a welcome letter approximately three months after the participant begins receiving meals, which will describe the study and that a member from the research team will contact them to schedule the interview unless they "opt-out" of the study. Within this introductory letter, we will include a refusal to participate postcard that caregivers will be asked to complete and return if they do not wish to participate in the study. For caregivers from whom we do not receive a refusal postcard, telephone call, or email indicating that they are not interested in participating, we make no more than 3 attempts to contact them between the hours of 10am and 7 pm, in their representative time zones.

A trained Brown University research team member will be responsible for calling caregivers, conducting an informed consent process over the telephone, and completing the interview. Recruitment will continue with replacement until we achieve our target total sample size of 2-10 caregivers at each program. Interviews aim to understand participants' and caregivers' experiences receiving meals, their interactions, if any, with the person who delivers meals (i.e., driver or mail carrier), how the meal service may have impacted their lives (e.g., responsibilities, stress, burden), and their satisfaction with the meal services.

5. **Compensation / Reimbursement**

   Participant incentives are included for persons with dementia and caregivers who are participating in the telephone interviews in the study. Persons with dementia will be mailed a $50 pharmacy gift card for a 30-minute telephone interview, an amount that is typically offered for interview studies in populations with, or at risk for AD/ADRD. Caregivers will be mailed a $50 pharmacy gift card for a 30-45-minute telephone interview.

6. **Potential Research Risks / Discomforts to Participants.** *In order to approve the research, the IRB must consider the risks posed to participants by the research and any efforts to mitigate those risks. The IRB needs to determine that the risks have been both minimized and are reasonable in relation to the anticipated benefits to participants, as well as to the importance of the knowledge that may be gained. The IRB will also consider whether the informed consent process provides potential participants with an accurate and fair description of the risks or discomforts.*

The pilot is considered a minimal risk study. Potential risks to individuals receiving meals include mild distress due to not receiving the preferred type of meal. While both options (i.e., meals delivered multiple times a week by the MOW program vs. frozen, mailed meals) are preferable to no meals at all, some participants may have a strong preference for one over the other and experience disappointmentif randomized to the non-preferred option. However, given that participants will have expressedan interest in receiving meals and are on the waiting list, receiving any meals will be preferableto receiving none.

There is also a risk of breach of confidentiality to individuals receiving meals and those participating in interviews. The risk of breach of confidentiality will be minimized to the greatest extent possible by the stipulations set forth in the Data Use Agreement with CMS, as well as by the measures described below.

Protecting Participant Data

One risk of the study is a breach of confidentiality with the use of identifiable data. All research staff involved in the study will receive training in the protection of human subjects. The procedures described here address our efforts to minimize the risk of breach of confidentiality. All data management and analyses will be conducted by the Brown Center for Gerontology & Healthcare Research, leveraging its administration and computing infrastructure. Researchers and staff have many years of experience working with identifiable data files on a large scale and have numerous security measures in place to ensure the integrity and confidentiality of the data. CMS data will be covered under the strict terms of a data use agreement (DUA). At the conclusion of this study, or by the date of retention identified in the DUA, a CMS "Certificate of Disposition" certifying the proper destruction of all data obtained from CMS will be sent to CMS. All non- CMS data will be handled in a similarly restrictive manner. In addition, all output containing individual identifiable information is treated as confidential data. This information is never transferred electronically via email.

Identifiable information will not be attached to any research documents, and all data will be identifiable only using the unique identifier specific to this study. All members of the research team have experience collecting data from participants and using secondary data that contains private health information. None of the study team members have had a breach of confidentiality associated with their research.

For participants with dementia and caregivers taking part in the interviews, they may experience some discomfort in responding to the interview questions as they may bring up unpleasant feelings or memories (for example, what things were like before they began receiving meals). However, these risks do not exceed those of topics considered in everyday life and are minimal. In order to protect against these risks, the researchers will remind participants that they are free to stop the interview and recording at any time and it will not impact the receipt of meals. The interviewers will also offer participants to take a break during the interview if there is any indication that the interview becomes too difficult or uncomfortable.

The justification for including persons with dementia and their caregivers in this study is the scientific value of understanding how home-delivered meals help people with dementia, who make up approximately 30% of all MOW clients. Given this population's unique needs, it is not possible to conduct informative research for this research with other populations (i.e., we have selected this population for scientific reasons; it is not a convenience population). In sum, this

Brown University IRB Original Approval: 12/10/2020
Brown University IRB Amendment Approval 02/25/2021
Brown University IRB Amendment Approval 10/06/2021
Brown University IRB Amendment Approval 05/11/2021
Brown University IRB Amendment Approval 08/19/2021

population is being enrolled because the findings of the research will directly inform the delivery of services to people with dementia in the future – and is needed to improve service quality for this population in support of their goal of living independently in the community.

7. **Potential Benefits of the Research. NOTE: Compensation for participation is not a benefit and should not be included in this section.** *In order to approve this research, the IRB must determine that the potential benefits to research participants are reasonable in relation to the potential risks. Very often, research at Brown does not include potential direct benefits to participants, but may only benefit society as a whole by helping researchers.*

   Older adults with dementia on MOW waiting lists who agree to participate will receive meals, as opposed to remaining on the waiting list to receive meals. Further, this study would provide information that would assist healthcare systems, providers, payers, programs, persons with dementia, and their caregivers. The minimal risks to subjects are reasonable for the importance of the information that will help understand the comparative effectiveness of the two predominant forms of meal delivery for persons with dementia.

   For individuals participating in interviews, they will not directly benefit from being interviewed. However, their participation will help us better understand the experiences of older adults with dementia who receive home-delivered meals.

Brown University IRB Original Approval: 12/10/2020
Brown University IRB Amendment Approval 02/25/2021
Brown University IRB Amendment Approval 05/11/2021
Brown University IRB Amendment Approval 08/19/2021
Brown University IRB Amendment Approval 10/06/2021

PART IV. INFORMED CONSENT

Informed consent is a *process*, not just a form. The IRB must ensure the informed consent process clearly discloses and facilitates the understanding of all information needed to make an informed decision to participate while promoting the voluntariness of participation.

Please use the Brown consent / assent templates and related guidance on the HRPP Forms & Templates page to develop your consent forms.

1. Describe the informed consent process

Telephone Interviews with Participants: One day prior to the scheduled telephone interview, the project coordinator will confirm the appointment with the participant via phone call, email, or text message according to the participant's preference. At the beginning of the telephone interview, co-investigators Dr. Emily Gadbois Dr. Renee Shield, and/or Dr. Katie McAuliff will review the study procedures and will go over the consent form with study participants, reviewing the form with them and periodically asking them questions about the consent process to be sure that they understand what they are consenting to. (Please see the interview guide for additional information.) Dr. Gadbois, Dr. Shield, and Dr. McAuliff have years of experience conducting research interviews, including interviews with vulnerable populations such as those with cognitive impairment. Nonetheless, Dr. Gadbois, Dr. Shield, and Dr. McAuliff will participate in training by Dr. Michelle Hilgeman, a study Co-I and geriatric psychologist, who has extensive research experience consenting and conducting research interviews with individuals with memory impairment. Dr. Gadbois, Dr. Shield and Dr. McAuliff will answer any questions the participant might have and remind them that they may opt out of the interview at any time. If a participant agrees to be interviewed, they will give verbal consent to participate that will be recorded by the Brown researcher. The consent forms will address audio recording consent. Participants who decline audio recording may still participate in the interview. In their case, a research team member will take notes during the interview. While the informed consent document will be sent with the welcome letter, a second blank copy of the informed consent form will be mailed to the participant within one week if they request. The consent form will have contact information for both the study PI and the Brown University IRB if they have any questions or concerns. Because interviews will be conducted by telephone rather than in-person, we will seek a waiver of documentation of informed consent.

Telephone Interviews with Caregivers: One day prior to the scheduled telephone interview, the project coordinator will confirm the appointment with the caregiver via phone call, email, or text message according to the caregiver's preference. At the beginning of the telephone interview, the trained research team member will review the study procedures and will go over the consent form with caregivers, reviewing the form with them and periodically asking them questions about the consent process to be sure that they understand what they are consenting to. (Please see the interview guide for additional information.) This research team member will also answer any questions the caregiver might have and remind them that they may opt out of the interview at any time. If a caregiver agrees to be interviewed, they will give verbal consent to participate that will be recorded by the Brown researcher. The consent forms will address audio recording consent. Caregivers who decline audio recording may still participate in the interview. In their case, the research team member will take notes during the interview. While the informed consent document will be sent with the welcome letter, a second informed consent form will be mailed to the caregiver within one week of the interview if they request. The consent form will have contact information for both the study PI and the Brown University IRB if they have any questions or concerns. Because interviews will be conducted by telephone rather than in-person, we will seek a waiver of documentation of informed consent for the caregiver interviews, as well.

Brown University IRB Original Approval: 12/10/2020
Brown University IRB Amendment Approval 02/25/2021
Brown University IRB Amendment Approval 05/11/2021
Brown University IRB Amendment Approval 08/19/2021
Brown University IRB Amendment Approval 10/06/2021

2. Facilitate Understanding

Telephone Interviews with Participants: A trained co-investigator on the research team (Dr. Emily Gadbois Dr. Renee Shield, and/or Dr. Katie McAuliff) will assess the participant's ability to understand and to express a reasoned choice concerning the purpose of the study and the information relevant to their participation and consequences of participation for the subject's own situation. Dr. Gadbois, Dr. Shield, and/or Dr. McAuliff will determine if all of the following standards are met: Did the participant "make a clear choice"? Did the participant show "understanding"? Did the participant show "reasoning/rational reasons"? Did the participant show an appreciation of the personal risks/benefits of participation in the study? This determination will be accomplished through a four-item informed consent understanding checklist included as an attachment. For individuals who do not pass the 4-item consent capacity checklist, they will be informed that they are ineligible to participate in the interviews. If this event occurs, Dr. Gadbois, Dr. Shield, or Dr. McAuliff will allow as much time as needed to allow the expression of any concerns. While Dr. Gadbois, Dr. Shield, and Dr. McAuliff have years of experience conducting research interviews, including vulnerable populations such as those experiencing cognitive impairment, they will receive training on ensuring understanding by Dr. Michelle Hilgeman, a geriatric psychologist with extensive research experience consenting and interviewing individuals with dementia and their caregivers. In addition, Dr. Hilgeman and the PI will regularly meet with Dr. Gadbois, Dr. Shield, and Dr. McAuliff to review their responses on the consent understanding checklist, answer any questions, and discuss any challenges.

Telephone Interviews with Caregivers: A trained research team member will conduct the informed consent process with the Caregiver. There is no reason to believe that the Caregiver will experience any challenges comprehending the informed consent form as they are the person listed as the emergency contact for the MOW participant. Therefore, the research team member will assess the Caregiver's understanding of the consent process through encouraging them to ask questions during the review of the consent form. Dr. Hilgeman and the PI will regularly meet with the research team member conducting the interviews and consent process to answer any questions and discuss any challenges.

3. Documentation

Please see below for a request for a waiver of documentation of informed consent for interviews.

4. Additional Considerations

We request a waiver of documentation of informed consent for the telephone interviews with caregivers and participants. We believe that we meet the criteria for a waiver as described in 45 CFR 46.117(c)(ii):

The research presents no more than minimal risk of harm to subjects and involves no procedures for which written consent is normally required outside of the research context.

The interviews will be conducted over the phone and ask minimal risk questions where we anticipate little to no distress as a result of the interviews. Consent would not normally be required for a telephone conversation of this nature, and it would not be practicable to obtain signatures because the interviews are being conducted over the telephone. We request a waiver of informed consent for initial enrollment to be randomized to receive one of the Two types of meals (meals delivered by the MOW programs multiple times a week vs frozen, mailed meals). We believe enrollment in the two types of meal deliveries and analysis using routinely collected data meets the five criteria for a waiver as described in 45 CFR §46.116 f 3 (i-v)and below:

(i) The research involves no more than minimal risk to the subjects;

Brown University IRB Original Approval: 12/10/2020
Brown University IRB Amendment Approval 02/25/2021
Original Approval: 12/10/2020
Brown University IRB Amendment Approval 05/11/2021
Brown University IRB Amendment Approval 08/19/2021
Brown University IRB Amendment Approval 10/06/2021

Our study proposes to provide meals via the two predominant modes of meal delivery currently used by home-delivered meals programs and contracted for by healthcare entities to food insecure, homebound, community-dwelling older adults with ADRD. Because we are recruiting from an existing MOW waitlist, these individuals have already expressed a desire to receive meals and shared their information with MOW, but they are not currently receiving meals because of limitations in program capacity (i.e., inadequate funding to meet demand for meals). The primary outcome analysis for the trial is through secondary data analysis of routinely collected administrative data; therefore, no additional data collection is required or burden will be placed on the enrolled sample. Enrollment and receipt of meals puts these individuals at no greater risk than everyday life.

(ii) The research could not practicably be carried out without the requested waiver or alteration;

Because we are interested in understanding the impact of receiving meals on the time spent in the community among individuals living with dementia, we need to enroll individuals with varying levels of dementia severity. While some individuals with dementia may retain capacity to consent to research (a diagnosis, especially a self-reported diagnosis one, is not determinative), restricting the study to only those able to provide consent would limit the generalizability of our findings, as we would likely only be able to enroll those with less-severe ADRD. If we were to conduct a capacity assessment and sought to include people who lacked capacity to consent to research, we would need to identify a legally authorized representative (LAR). Most individuals with ADRD living in the community do not have a LAR appointed. Some studies have found rates as low as 0% of individualswith mild dementia and only 11-23% of those with moderate to severe dementia have legal documents in place such as guardianship, trusteeship, proxy, or power of attorney (Ruggieri & Piccoli, 2003). Furthermore, LAR rates vary by race and socioeconomic status and are lowest among racial minorities and those with less education(AARP, 2000). Our prior work has found that MOW clients are more likely to be racial and ethnic minorities and low-income than the general population of older adults. In addition, MOW programs do not collect information about clients' LARs. Therefore, there is no mechanism for identifying LARs for potential participants in this study. Finally, MOW programs do not collect information to identify possible surrogates for consent. In addition,surrogate consent laws for research participation vary by state, and it would not be possible to identify a consent process that is consistent across the programs that will participate in the follow-on pragmatic trial and located
in 5 states. Taken together, requiring consent would result in bias through differential selection into the study and comprise the validity and overall scientific value of the study findings.

Waiving the informed consent process allows us to include individuals who may not have capacity to give consent, the very group who is most at risk of nursing home placement and whom we hypothesize would experience the greatest effect of this low-risk intervention that they have already signed up to receive but are currently unable to access because of program limitations. Notably, we are not asking for a waiver of consent because we assume that no participants can consent; rather, we are asking for a waiver because including only those individuals for whom consent can be obtained (either directly from the participant or from a LAR) would prohibit conclusions to be drawn or bias the sample such that our conclusions would be skewed. In addition, there are two other considerations. First, a goal of this pilot is to test the feasibility of the procedures for enrolling individuals with ADRD in a study of the effects of receiving meals from MOW on time in the community. Our team will use the experience from this pilot study to inform enrollment procedures in a follow-on pragmatic trial. While we are only including 235 individuals in this study, the larger follow-on pragmatic study will include 2300 individuals with ADRD. In that case, the sample size is so large that it would be impracticable to conduct capacity assessments with each individual. Thus, it will be informative to test waivers here.
Second, our power calculations to derive our needed sample size for our larger trial were based on our prior observational study that included the population of MOW clients with varying degrees of dementia severity. We are unable to conduct a power analysis and derive a needed sample size for our follow-on trial only among those who have capacity to consent/less severe impairment; this information is simply not available. Therefore, by excluding individuals who may not have capacity to consent, we may be underpowered to detect an effect for our primary outcome, time to nursing home placement, in the larger trial.

Brown University IRB Original Approval: 12/10/2020
Brown University IRB Amendment Approval 02/25/2021
Brown University IRB Amendment Approval 05/11/2021
Brown University IRB Amendment Approval 08/19/2021
Brown University IRB Amendment Approval 10/06/2021

(iii) *If the research involves using identifiable private information or identifiable biospecimens, the research could not practicably be carried out without using such information or biospecimens in an identifiable format;*

We are proposing to use routinely collected administrative data for the analysis of the primary and secondary study outcomes. We require the data in identifiable format in order to link data received from the MOW program to data received from the Centers for Medicare and Medicaid Services (CMS) at the client level and could not conduct the study without the link. A waiver of consent is regularly sought out for analyses that are conducted using linked CMS data. Measures to protect the confidentiality of the information are described below.

(iv) *The waiver or alteration will not adversely affect the rights and welfare of the subjects;*
This study population with dementia is unique. While some individuals with dementia could successfully complete a consent process over the telephone, we would likely lose the ability to enroll those most likely to be impacted by meal delivery type (i.e., individuals with more than mild cognitive impairment or mild AD) confounding our ability to address the primary aims of this study. The level of understanding necessary to comprehend the study design during a telephone conversation with someone they have not met (i.e., consenting to receive meals, which they have already requested) may introduce added burden and distress. In addition, for those who have previously requested meals but do not have capacity to consent to receive meals through this study, resulting in a return to the MOW waiting list, could be distressing. Some people may worry that individuals on the MOW waiting list would feel they had no reasonable alternative to agree to participate in research in order to receive meals or worry that they will not keep their place on the waiting list if they decline to participate. By waiving the consent process (as we have done in previously funded studies), providing meals to individuals, and linking MOW program data with claims data addresses these concerns and is consistent with the pragmatic trial design.

(v) *Whenever appropriate, the subjects or legally authorized representatives will be provided with additional pertinent information after participation.*
Finally, we will ensure that participants are provided information about the study upon completion of the study. We will do this through including a description of the study and results in the MOW programs' newsletters, which is shared with all clients and caregivers. Included will be the contact information of who to call for additional information. In addition, we will mail a study summary to all participants and caregivers who took part in the interviews.

Brown University IRB Original Approval: 12/10/2020
Brown University IRB Amendment Approval 02/25/2021
Brown University IRB Amendment Approval 05/11/2021
Brown University IRB Amendment Approval 08/19/2021
Brown University IRB Amendment Approval 10/06/2021

PART V. USE OF SECONDARY DATA / BIOSPECIMENS

For research that involves the use of identifiable secondary data / biospecimens, including coded data from which you may be able to ascertain participant identity.
If your research does not involve identifiable secondary data / biospecimens, proceed to **PART VI. DATA SECURITY ASSESSMENT**

1. From what source(s) will you acquire or access the data / biospecimens?

Secondary data sources will include participant data from MOW programs as well as CMS data.

We will utilize research identifiable (RIF) claims, enrollment, assessment, data from the Centers for Medicare and Medicaid Services (CMS). We will acquire these data through the strict terms of a data use agreement (DUA) that we will enter into with CMS.

MOW participant data (e.g., first and last name, telephone number, date of birth, race/ethnicity, gender, address, SSN, client ID, emergency contact information, living arrangement, participant dementia/cognitive impairment, diagnoses, functional status) as well as programmatic data (e.g., meal delivery start date, meal delivery end date, breaks in service for meal delivery (including reasons for break in meal delivery service, issues/complaints/satisfaction from participant, caregiver, or delivery person, and total number of meals delivered to participant) will be shared with Brown investigators via secure methods and downloaded to a secure Brown University server.

As part of this March 2020 amendment we are adding two new data sources (both secondary data from MOWA):

- Data from a 2013 pilot conducted by MOWA
This pilot was a programmatic evaluation conducted by MOWA and was not considered a research study

- Programmatic data from 13 MOW programs.

Both of these datasets have already been disclosed to us without a DUA as part of a pervious project, and MOW is aware we will be reusing these datasets for this project.

Client information is covered by a Non-Disclosure Agreement executed between Brown and MOWA. We will explore obtaining individual Data Use Agreements with participating programs if necessitated by the programs, and will file IRB amendments as needed to reflect the need for DUAs if MOW requires them.

2. Describe the type(s) of data and date range(s) of the data you will use and the characteristics of the study research population (e.g., age range, sex, and any other pertinent demographic information.)

The inclusion criteria for the current pilot include being on the waiting list, 66 years of age or older (to allow for Medicare enrollment and a one-year baseline lookback, so anyone over the age of 65 will be included) and self-reporting a diagnosis of dementia or cognitive impairment. Age and self-reported diagnosis come from the MOW programs' intake records.

Additionally, to determine the efficacy of two different modes of meal delivery (frozen vs. meals delivered by MOW programs multiple times a week), we will leverage a previous pilot trial that was completed in 2013 with 626 participants randomized to three arms: 1)daily delivered meals, 2) frozen, weekly delivered meals, and 3) remain on the waiting list. From the

2013 pilot, we have SSNs for 302 participants (123 participants in the daily-delivered meal arm; 79 participants in the frozen, weekly delivered meals; and 100 participants who remained on the waiting list) that we are requesting linkage for CMS data to examine their characteristics, healthcare utilization, and time to nursing home placement. Eligibility criteria for the 2013 pilot data include being enrolled in the pilot. We will also leverage data from 13 MOW programs (which includes 14,000 MOW participants) that we previously linked to CMS claims using probabilistic methods under a previous DUA. Criteria for inclusion forthis cohort include MOW recipients ages 66 and older receiving meals from one of the 13 MOW programs.
For each cohort, we will track their healthcare utilization, including time to nursing home placement. Additionally, for each cohort, we are also requesting from CMS a year of data prior to them entering the cohort in order to compare their characteristics leading up to the receipt of MOW to see how similar they are to one another. For the 2013 pilot data, this will entail using data starting from February 6, 2012, and for the 13 MOW programs, starting on January 1, 2009 (with lookback to 2007 in order to characterize MOW clients' healthcare utilization prior to enrollment in MOW as well as their diagnoses and functional status changes leading up to enrollment).

For the current pilot, this will entail using data starting from April 2020.

Inclusion of Women. Women will not be oversampled but will be included as they appear naturally in the populations described above.

Inclusion of Minorities. All races and socio-economic statuses will be represented in the data as they appear naturally in the target data populations.

Inclusion of Children. Not applicable - Our study examines the population of MOW clients age 66 and over.

3. We request a waiver of informed consent for the release of the MOW and CMS data under the Common Rule. We acknowledge that:

(1) *The research involves no more than minimal risk to the subjects;* the only risk is the breach of confidentiality of the use of the data. Our safeguards are detailed in the application.

(2) *The waiver or alteration will not adversely affect the rights and welfare of the subjects;* No contact with individuals is necessary for the completion of this project since all data will have been previously collected for non-research purposes. Results will be reported in aggregate and no published reports will include information on specific individuals.

(3) *The research could not practicably be carried out without the waiver or alteration;* We are proposing to use routinely collected administrative data for the analysis of the primary and secondary study outcomes. We require the data in identifiable format in order to link data received from the MOW program to data received from the Centers for Medicare and Medicaid Services (CMS) at the client level and could not conduct the study without the link. A waiver of consent is regularly sought out for analyses that are conducted using linked CMS data. Measures to protect the confidentiality of the information are described below

(4) *Whenever appropriate, the subjects will be provided with additional pertinent information after participation.* We will not have direct access to any individuals not enrolled in the randomization portion of the study. Therefore, we will include a study summary and results in

MOW programs' newsletters. There we will also include information about who to contact to learn more about the study. For those we do have direct access to (i.e., participating in the interviews), we will directly mail a summary of study results at the completion of the study.

4. HIPAA and Protected Health Information (PHI):

At the individual level, this project will use secondary data sources from the Centers for Medicare and Medicaid Services (CMS), and Meals on Wheels (MOW). Direct identifiers such as social security number, first and last name, telephone number, and address will be present in the MOW data (not in the CMS data), but other identifiers will be present in both data. The data contain personal health information (PHI), and the CMS data will be disclosed to the research team by the CMS under the strict terms of a Data Use Agreement (DUA). Shells of the DUA form and executive summary (which includes a drafted data management section) are included with this request.)

As part of the CMS DUA, we request that a HIPAA waiver of authorization be granted by the Brown IRB in order to allow CMS to release the PHI to us. MOW may also require a DUA for their data, we are not yet sure about that and will update the IRB and the Brown DUA office before we pursue a DUA.

We acknowledge that:

The PHI use or disclosure involves no more than minimal risk to the privacy of individuals.
Part VI (3) and Part VI (6), as well as the Protections Against Risk section below outlines how we will accomplish protecting identifiers from improper use and disclosure, as well as our plan to destroy identifiers at the earliest opportunity consistent with research. In addition, we confirm that the PHI will not be reused or disclosed to any other person or entity except (a) as required by law, (b) for authorized oversight of the research study, or (c) for other research for which the use or disclosure of the PHI is permitted by the Privacy Rule.

The research could not practicably be conducted without the requested waiver or alteration.

The CMS data used for this project have been previously collected for non-research purposes. Depending on the cohort as described above, we will use data from 2007-2021. No contact with individuals is necessary or desired for this study. It would be impossible for us to obtain individual permission from these individuals.

The research could not practically be conducted without access to and use of the PHI.

The research could not be conducted without access to these individually identifiable data since CMS does not release assessment data (i.e., MDS) in less restricted form. We need the PHI in the MOW data to link to their CMS data, perform the randomization, and for follow-up. Additionally, we need the PHI in order to determine the efficacy of the two different modes of delivery on time to nursing home placement (primary outcome) as well as days in community and hospital transfers (secondary outcomes) among food insecure older adults with ADRD.

<u>Protections Against Risk</u>

Dr. Kali Thomas will ensure that systems are in place to minimize the risk of breach of

Brown University IRB Original Approval: 12/10/2020
Brown University IRB Amendment Approval 02/25/2021
Original Approval: 12/10/2020
Brown University IRB Amendment Approval 05/11/2021
Brown University IRB Amendment Approval 08/19/2021
Brown University IRB Amendment Approval 10/06/2021

confidentiality for all data in this trial. Brown maintains numerous confidential databases, including MDS and Medicare files, and has a high level of security built into its computing systems. Dr. Thomas will work closely with MOWA leadership to develop protocols to maintain a high level of data confidentiality for extracting and merging data from their programs' records. She will also work with our DUA manager (Dr. Julie Lima) and our IT Director (Jeffrey Hiris).

All data transfers will be managed by the IT Director. Those between MOW programs and Brown University will be done by secured methods, likely via a secure stfp server. Data will be encrypted with an Advanced Encryption Standard (AES) of at least 256-bit encryption algorithm. This is a block cipher adopted as an encryption standard by the U.S. government. The National Security Agency (NSA) has deemed the use of 256- bit AES encryption algorithm as secure enough for U.S. government top secret information. Passwords will be transmitted out of band, either verbally, email, or via an SMS text message. Identifiers for MOW clients will be shipped to HealthApt to pull their Medicare and assessment data from the Centers for Medicare and Medicaid Services. To maintain and assure the security of identifiable information shipped to HealthApt, all finder files containing secure information will, again, be encrypted to at least AES-256 standards. The files will be password protected with a password no less than 10 digits in length and must contain a combination of letters and numbers. The data shipment will be sent by a courier service with tracking capabilities, such as FedEx, UPS, DHL, or Registered Mail options. The password and the DUA# to which the finder file belongs will be emailed to HealthAPT at CMSdata@gdit.com.

As mentioned above, The Center for Gerontology and Health Care Research maintains numerous confidential databases and has a high level of security built into its computing system. The computing infrastructure consists of a VMScluster, which houses all substantial data, a group of Windows servers that provide computer services and infrastructure support for client systems, and client Windows PCs through which all Gerontology users access our systems. Network security is provided by a combination of University firewalls, local network access controls, and continuous auditing and monitoring for security breaches. All access from systems external to the local area network (LAN) is limited to encrypted channels, e.g., Secure Sockets Layer (SSL) for Virtual Memory System (VMS) terminal sessions, or Virtual Private Network (VPN) connections for LAN file-sharing access. (Unencrypted access is provided for the Center's external website and general email support functions.) The VMScluster acts as a file server for 186 Windows clients, and file access controls are consistently applied whether access is from the VMS or Windows environment. Security within the system is applied via Access Controlled Entry (ACE) attached to all files on all systems. Security is applied uniformly to all files within a subtree of any file system, with the general rule that groups of users sharing a common task may read each other's files but, in most cases, not write to each other's files. VMS provides a highly-secure programming environment with ACEs applied to all objects and extremely controlled access to the larger system for individual users, as well as a versioning file system, secure batch queues and distributed processing, and efficient backup and recovery procedures. Windows clients are limited to a subset of these services (e.g., there is no way for file version information to be shown to Windows clients), but otherwise access is secured as for any other method of accessing data.

Personally-identified data, as well as partially de-identified data, are housed in files that are restricted to systems management or to programmers who have been identified as custodians. No data are ever moved to more "public" spaces without identification information being stripped or non-reversibly encoded. Encoding is generally done via fairly large Roman cyphers applied iteratively to the original character string. No reverse encoding is ever generated nor maintained. Any matching between personally-identified data sources is done within a secured area prior to any

Brown University IRB Original Approval: 12/10/2020
Brown University IRB Amendment Approval 02/25/2021
Brown University IRB Amendment Approval 05/11/2021
Brown University IRB Amendment Approval 08/19/2021
Brown University IRB Amendment Approval 10/06/2021

data being exported. Windows servers that house partially de-identified data have matching ACEs applied so that access restrictions are applied consistently with VMS-based data. It should be noted that since we use demographic covariates for many of our analyses, even the encoded data are best considered partially de-identified. ACEs restrict access to all data, such that access to any data elements on the servers is limited to those staff authorized to make such access. Authorizations are, in turn, granted by the core system's support staff upon request from a PI or other appropriate data owner. All users authorized to access systems have access to some storage, which is considered "general file sharing," but, by convention and policy, all individual or otherwise restricted data are prohibited from being stored on such space. Desktop systems are authorized to specific users, and it is assumed that they will store data that they have been authorized to work with on such local systems. The LAN is switched, yielding a reasonable amount of security between clients and servers within the LAN. Desktop systems are required to run current anti-virus software and are prohibited from running local file-sharing software. External analyses are run periodically to verify the security of the systems within the LAN.

Similarly, the Windows servers that support the LAN are configured as a local, isolated, secure, collapsed Active Directory (AD) forest local to our LAN, Domain Name System (DNS), and Dynamic Host Configuration Protocol (DHCP); other critical services are secured within the context of the local forest and are not accessible externally with the exception, of course, of VPN or Remote Desktop Protocol (RDP) access from authorized client systems. Extensive monitoring is done from the VMS cluster to ensure the health and stability of the Windows forest structure and the individual servers within it.
In summary, the VMS computer system is highly secure and accessible only to authorized users. Within the group of authorized users, access to project data is restricted to individuals who are authorized to work on that specific research project. Access to identifiers is further restricted to only the systems manager and any person designated to do raw data merges across files. Furthermore, Gerontology employees have signed an oath of confidentiality, and its violation is sufficient grounds for immediate termination. Results of this research will be presented for large groups so that individuals cannot be identified. We will suppress the reporting of any data where the number of individuals is less than 11.

5. Do any of the source(s) require a Data Use Agreement (DUA) or other Agreement that requires institutional signature to obtain, access or use the data / biospecimens? ☒Yes ☐No

If "yes," please include a copy of the Agreement(s) with this submission and also follow the Data Use Agreement review and signature processes.

PART VI. DATA SECURITY ASSESSMENT

**1. Will you be collecting biospecimens?**	
☐Yes ☒No	If “yes,” please review the Institutional Biosafety Committee (IBC) webpage. A supplemental IBC review may be required.
**2. Do the study data / biospecimens include identifiers? Video and audio recordings are considered identifiable.**	
☐No	If “no,” I affirm that I have read and will abide by the Level 1 Risk Minimum Security Standards: ☐Yes ☐No<br><br>Proceed to Question #3.
☒Yes	If “yes,” answer the following questions.<br><br>A. Describe the identifiers associated with the data / biospecimens.<br><br>For the MOW data: first and last name, telephone number, date of birth, race/ethnicity, gender, address, SSN, Medicare ID number, MOW client ID, marital status, emergency contact information, living arrangement, participant dementia/cognitive impairment, diagnoses, functional status) as well as programmatic data (e.g., meal delivery start date, meal delivery end date, breaks in service for meal delivery (including reasons for break in meal delivery service, issues/complaints/satisfaction from participant, caregiver, or delivery person, and total number of meals delivered to participant)<br><br>For the CMS data: dates directly related to an individual such as birth, death, dates of service, enrollment, assessment, hospital admission, discharge (as found in claims, assessment, and enrollment Research Identifiable Files (RIF) from the Centers for Medicare and Medicaid Services), age information for those above 89, gender, race/ethnicity, medical conditions, that in combination with these other items may be considered uniquely identifying.<br><br>B. Justify why identifiers are required to conduct the research.<br><br>CMS does not release many of these files in less restricted form, and those that are available in less restricted Limited Data Set (LDS) form are not sufficient for our purposes because they do now allow us to follow an individual. We must be able to follow individuals over time and through the health care system, requiring consistent identifiers.<br><br>MOW data with identifiers is needed for the randomization, and to link to the CMS data.<br><br>C. Described the proposed research use of the identifiable data / biospecimens.<br><br>We require the data in identifiable format in order to link data received from the MOW program to data received from the Centers for Medicare and Medicaid Services (CMS) at the client level and could not conduct the study without the link

Brown University IRB Original Approval: 12/10/2020
Brown University IRB Amendment Approval 02/25/2021
Original Approval: 12/10/2020
Brown University IRB Amendment Approval 05/11/2021
Brown University IRB Amendment Approval 08/19/2021
Brown University IRB Amendment Approval 10/06/2021

	D. Self-classify the Risk Level of these data / biospecimens (select the *highest level of risk* for all data / biospecimens being collected).<br>☐ Level 2 Risk<br>☒ Level 3 Risk

3. How will study data / biospecimens be collected?

☐ Brown desktop
☐ Laptop
☐ Departmental server
☐ CIS managed server
☐ Brown Qualtrics
☐ REDCap; Please describe what instance of REDCap is being used (Brown does not have an instance of REDCap): Click or tap here to enter text.
☐ Amazon Mechanical Turk (MTurk)
☐ Text messaging �You must complete the Text messaging section after completing Qs 3 – 5.
☐ Mobile App (on tablet, iPad, Phone) �You must complete the Mobile App section after completing Qs 3-5.
☐ Zoom
☐ Digital records (audio / videoconferencing tools, digital photographs); please describe the tool: Click or tap here to enter text.
☐ Paper records (including physical photographs). Please describe, including how you will securely store the paper records: Click or tap here to enter text.
☐ Web-based site / survey / other tool not listed above �You must complete the Web-based Other section after completing Qs 3 – 5.
☒ Other; please describe:

CMS and MOW data will already have been collected for non-research purposes. Much of the CMS data will already be in-house, anything not in house will be sent to us on discs. The MOW data transfer will be done by secured methods, likely via a secure stfp server. See "protections against risk" above for more information.

Interviews will be conducted over the telephone and recorded. Recorded responses will be collected, stored, and examined at Brown University and will be kept in a secured area in password-protected server files. Any transcribed interviews will be kept in locked cabinets in the Principal Investigator's office. All interviews will be given a unique identifier (e.g., 001, 002) and will not contain nor be linked to any identifiable information.

4. Who will have access to the study data / biospecimens?

☐A. Brown PI only. How will unauthorized access by others be prevented?
Click or tap here to enter text.

☒B. Brown PI and other Brown research team members. How will unauthorized access by others be prevented?

Personally-identified as well as partially de-identified data is housed in files which are restricted to the systems manager or to programmers who have been identified as Custodians or project staff for a specific data collection process which requires use of personal identifiers. No data is ever moved to more "public" spaces without personal identifying information being stripped and replaced by unique and non-informative person IDs. Insofar as CMS provides such IDs (e.g., a study-specific beneficiary ID) we have used them. Any matching between personally-identified data sources is done within a secured area prior to any data being exported.

It should be noted that since the Center uses demographic covariates and provider IDs for many of our analyses, even the encoded data is best considered partially de-identified. ACEs restrict access to all data housed by the Center, such that access to any data elements on the servers is limited to those staff authorized to make such access. Authorizations are in turn granted by the core system's support staff, upon request from a PI or other appropriate data owner. All users authorized to access Center systems have access to some storage which is considered "general file sharing," but by convention and policy all individual or otherwise restricted data is prohibited from being stored on such space.

Desktop systems are authorized to specific users and it is assumed that they will store data that they are authorized to work with on such local systems. The Center's LAN is switched, yielding a reasonable amount of security between clients and servers within the LAN. Desktop systems are required to run current anti-virus software and are prohibited from running local file-sharing software. External network scans are run periodically to verify the security of the systems within the LAN.

Similarly, the Windows servers which support the LAN are configured as a local, isolated, secure, collapsed AD forest local to our LAN. DNS, DHCP and other critical services are secured within the context of the local forest and are not accessible externally (with the exception, of course, of VPN access from authorized client systems.) Extensive monitoring is done from a small VMScluster to ensure the health and stability of the Windows forest structure, and the individual servers within it.

☐C. Data will be shared with research collaborators external to Brown. This data sharing intent **must** be described as part of your consent process / form. Please describe how you will securely share / transfer the data outside of Brown:
Click or tap here to enter text.

Note that an Outgoing Data Use Agreement ***is required when sharing identifiable data external to Brown****. Please follow the procedures outlined* *here**. You do not need to submit a copy of a DUA to the HRPP. This will be linked by the ORI administratively.*

5. Where will the study data / biospecimens be stored?

☒ Departmental server
☐ CIS managed server
☐ Stronghold
☐ Campus file storage
☐ REDCap

☒Other. Please describe: Transcripts and tapes will be kept in locked cabinets in the Principal Investigator's office

6. If traveling with your data, describe how your data will be secured.

N/A

7. For how long will you retain identifiable data / biospecimens? How will you destroy identifiers when no longer required?

Some data for this project do contain names, social security numbers and other direct identifiers. Additionally, PHI including dates of birth, death, health services utilization, and geographic information smaller than the state will remain on the analytic files, making the data potentially identifiable. These data will be housed in a project space that is specific to the project and accessible only to anyone with permission to access the data according to our DUA and relevant agreements with MOW (more on the specifics of the environment the data are stored in can be found in part 3, above.)

At the end of the study, the project space will be deleted, all analytic files containing identifiable information will be destroyed, and a Certificate of Disposition will be signed and sent to CMS certifying the proper destruction of all applicable data.

Interview tapes, transcripts, and any related study materials will be destroyed after three years from publication of the findings and/or three years from the submission of the final report to NIA, whichever event is later.

8. Text Messaging (only complete if instructed above.)

A. Are you using the current text messaging service available on the device?

☐Yes ☐No	If “no,” you must also complete the Mobile App section.

B. Whose device will be used? ☐Participant’s personal phone ☐Brown-issued phone

C. Content of messaging: (If brief, insert here; otherwise, please provide as an attachment)

D. Is the communication one-way or two-way? ☐One-way ☐Two-way

9. Mobile App (only complete if instructed above.)

A. Name of the mobile app: Click or tap here to enter text.

B. Has this site / tool been reviewed by CIS IT Security?

☐Yes ☐No	If "no," answer the following:<br>a) Who created the site / tool (vendor name or off the-shelf app creatorname)?<br>Click or tap here to enter text.<br><br>b) Where is it hosted?<br>Click or tap here to enter text.<br><br>c) Is the site / tool scanned for security vulnerabilities? ☐Yes ☐No<br><br>d) What version of software is being used, if applicable: ☐N/A or<br>Click or tap here to enter text.<br><br>e) How are the data encrypted?<br>Click or tap here to enter text.
C. Whose device will be used? ☐Participant's personal phone ☐Brown-issued phone If Participant's person phone:	
a. How is the app downloaded to the device?<br>Click or tap here to enter text.<br><br>b. Is a password or PIN required for the app? ☐Yes ☐No	
D. Will data be stored on the device for any period of time?	
☐Yes ☐No	a. If "yes," please describe (i.e., queue on phone and then transmitted to server):<br>Click or tap here to enter text.<br><br>b. Is the app data encrypted on the device? ☐Yes ☐No
E. Device features mobile app can access ☐N/A<br>☐Device ID and call information<br>☐Identity<br>☐Contacts<br>☐Camera<br>☐SMS or chat<br>☐Storage<br>☐Device and application history<br>☐Phone<br>☐Photo / media / files<br>☐Microphone<br>☐Location<br>☐Other; please describe: Click or tap here to enter text.	
F. Will a third-party have access to research data through this app? ☐Yes ☐No	
G. Is data transmitted by the device?	
☐Yes ☐No	If "yes," how is it encrypted in transit?<br>Click or tap here to enter text.
H. Are phone numbers or mobile identification numbers stored with the data? ☐Yes ☐No	
**10. Web-based Other (only complete if instructed above.)**	

A. Name of the site / tool: Click or tap here to enter text.	
B. Has this site / tool been reviewed by CIS IT Security?	
☐Yes ☐No	If “no,” answer the following:<br>a. Who created the site / tool (vendor name or off the-shelf app creator name)?<br>Click or tap here to enter text.<br>b. Where is it hosted?<br>Click or tap here to enter text.<br>c. Is the site / tool scanned for security vulnerabilities? ☐Yes ☐No
	d. What version of software is being used, if applicable: ☐N/A or Click or tap here to enter text.<br>e. How are the data encrypted?<br>Click or tap here to enter text.
C. Is informed consent being obtained via this site / tool?	
☐Yes ☐No	If “yes,” how is re-identification prevented?<br>Click or tap here to enter text.
D. Does the technology allow for the explicit exclusion of the collection of IP address of the participant’s connection?	
☐Yes ☐No	If “yes,” will you use this option to exclude the collection of IP address?<br>☐Yes ☐No

Brown Qualtrics: CIS has pre-vetted Brown Qualtrics for collection/storage of up to Risk Level III data. Qualtrics is the preferred survey tool for all Brown research data collection.

REDCap: Brown does not currently have its own instance of REDCap. Access to REDCap through a Lifespan collaborator must be explicitly identified.

Data collection: The expectation is that data collection *devices* will only store data during active data collection. Data must then be transitioned to more secure long-term storage solutions.

Departmental/CIS managed servers: If data are collected/entered directly onto a Departmental or CIS managed server, **you must ensure** that the server meets the security standards described in the Minimum Security Standards for Servers based on the Risk Level of the data identified in 1D.

Brown University IRB Original Approval: 12/10/2020

PART VII. APPENDICES

Please complete & attach the following Appendices to this Application, as applicable.

Incl.	N/A	
☐	☒	Appendix A. Children as Subjects<br>*To be attached when minors are included as participants [please be aware of the age of majority for your specific research site(s)]*
☐	☒	Appendix B. Prisoners as Subjects<br>*To be attached when prisoners are included as participants.*
☐	☒	Appendix C. Use of Drugs<br>*To be attached when the research includes the use of FDA-regulated or unregulated drugs.*
☐	☒	Appendix D. Use of Devices<br>*To be attached when the research includes the use of FDA-regulated or unregulated devices.*
☐	☒	Appendix E. Prescription Drug / Medication Management<br>To be attached when study procedures include administering prescription medications to study participants
☐	☒	Appendix F. Mental Health Safety Plan<br>To be attached when participants may experience significant emotional distress, or be at risk of themselves or others.
☒	☐	Appendix G. Use of Protected Health Information (PHI) for Research<br>To be attached when study procedures include a plan to access, use or disclose Protected Health Information (PHI) of participants.
☐	☒	Appendix H. International Research<br>To be attached when study involves human subjects research outside the United States.
☐	☒	Appendix I. Advisor Appendix<br>To be attached when a graduate or medical student is the Principal Investigator.

PART VIII. ATTACHMENTS

Please attach the following materials to this Application for Expedited / Full Board IRB Review, as applicable.

**Incl.**	**N/A**	
☒	☐	Additional Investigator COI
☐	☒	Application for IRB Authorization Agreement (IAA)
☒	☐	Data collection materials (questionnaires, surveys, interview scripts, etc.)
☒	☐	Data Safety Monitoring Plan
☒	☐	Data Use Agreement from data provider(s)
☐	☒	DSMB Charter Template
☐	☒	HIPAA Authorization
☒	☐	Informed consent documents / scripts
☒	☐	Permissions, approval documents, and/or support letters
☒	☐	Recruitment materials (emails, flyers, letters, scripts, posters, brochures, etc.)
☐	☒	Request for Approval to Serve as Principal Investigator on a Human Subjects Research Application
☒	☐	Other: Consent Understanding Checklist, Study Cohort Table

PART IX. CONFLICT OF INTEREST

The Brown University Conflict of Interest Policy for Officers of Instruction and Research *("COI Policy")* defines the term "Investigator" as "the project director or principal investigator and any other person, regardless of title or position (e.g., full or part-time faculty member, staff member, student, trainee, collaborator, or consultant), who is **responsible** for the **design, conduct, or reporting** of sponsored research."

Using this definition of "Investigator," please ensure that all Investigators on this protocol answer questions (1) and (2) below. Attach additional sheets for any Investigators who are not the PI; additional sheets are available on the HRPP website.

☐	I am affiliated with Rhode Island School of Design and will abide by policies and procedures set forth by my institution.

1. Have you completed a conflict of interest disclosure (i.e. *COI Reporting Form*) within the past 12 months and is it accurate and up-to-date as of the time of this submission, as required by Brown's COI Policy? (If you have not completed this disclosure, access the InfoEd system here.)

☒Yes ☐No	If "no," please do so before submitting this Application

Original Approval: 12/10/2020

2. Do you have a significant financial interest (SFI) that is related to this research protocol?<br>"Related" could mean the research involves products, technology, intellectual property, or services made, owned, or provided by the entity/ies in which you have an SFI. It could also mean that the SFI could be affected by the proposed research or its results.	
☐Yes ☒No	If "yes," please identify the SFI and explain the relatedness:<br>Click or tap here to enter text.
3. Do you have a faculty advisor or other Brown investigators working on this study?	
☒Yes ☐No	Additional COI sheets for Investigators are attached to this Application.

PART X. PRINCIPAL INVESTIGATOR AGREEMENTS & RESPONSIBILITIES

A. Conduct of the Research

1. I accept responsibility for the ethical conduct of this research and protection of participants as set forth in the Belmont Report, Common Rule, and Brown University policies.
2. I accept responsibility for ensuring this research is conducted in accordance with:
   a) Sound research design and methods;
   b) The parameters of the research plan and activities described in this Application;
   c) The applicable terms of the grant, contract, or other signed funding agreements;
   d) Applicable laws and regulations, including those protecting the rights, safety and welfare of human subjects.
3. I certify that I am, or my faculty advisor is, sufficiently qualified by education, training and experience to assume responsibility for the proper conduct of this research. I accept responsibility for ensuring that all member of the research team have or will complete human
4. subjects CITI training before any work with participants or identifiable data / biospecimens begins.
5. I accept responsibility to personally conduct and/or directly supervise this research. I certify that I have sufficient time and resources to properly conduct and/or supervise this research.
6.

B. Ensuring and Maintaining Compliance

1. I will comply with relevant regulatory and institutional reporting requirements, including Brown University's *Reportable Events Policy*.
2. I understand that it is my responsibility to ensure that any research personnel, including myself, responsible for the design, conduct or reporting of the research declares any conflicts of interest related to this research. I will ensure that any changes that impact my or other research personnel's answers to the questions in PART IX. Conflict of Interest, are reported promptly to Brown's HRPP.
3. I will ensure that prospective agreement and/or informed consent is obtained and a copy is provided to participants, when appropriate.
4. If there are changes to the research described in this Application for Expedited / Full Board IRB Review that may impact the study's classification as Full Board or Expedited research, I will promptly notify the Brown HRPP of such changes.
5. I will notify the Brown HRPP when I have completed all activities involving human subjects or

Original Approval: 12/10/2020

identifiable participant data or identifiable biospecimens.

6. I will maintain approval, as applicable, with collaborative parties, including approvals from other countries or jurisdictions.
7. I will cooperate with any post-approval monitoring or auditing of study activities and/or study records as requested and/or required by the Brown ORI, the Brown IRB, funding entities, sponsors, and/or any federal or state regulatory agencies.

C. Study records, Reports and Documentation

1. I will comply by Brown's *Research Data and Research Materials Management, Sharing and Retention Policy*.
2. I will maintain all research protocol materials and consent materials for the duration of this study.
3. I will maintain research records for at least three years following the end of this research, or for a longer length of time if specified in applicable regulations or sponsor requirements. I will take measures to prevent accidental or premature destruction of these records.
4. I will abide by all terms of any Data Use Agreement (or equivalent agreement) related to this study, including those agreed to electronically (through an online attestation).
5. I will ensure that the data security measures for acquisition, collection, transfer and use of study data described in PART VI. of this Application are adhered to by all members of the research team.

By my signature below, I certify that I have read and agree to uphold all of the Agreements and Responsibilities in PART X.

Principal Investigator signature: **Date: 12/02/2020**

Kali S. Thomas

==

For IRB Use Only

Signature of the IRB: [illegible]

Date of IRB approval: 12/10/2020

Pilot Research Activities and

**Research Activity**	**Subject Group**	**Informed Consent Requirement**	**HIPAA Authorization Requirement**
Linking MOW client data to CMS administrative data	**All** MOW clients at 3 MOW program sites at time of data request.	Request waiver of Informed Consent	Request waiver of HIPAA authorization requirement
Clients from MOW program waitlist enroll to receive either drop-shipped meals or meals delivered by the MOW program multiple times a week. Those who enroll are randomly assigned meal type delivery.<br>Those who do not enroll remain on the MOW waitlist. Client data will beshared with the research team.	**We will enroll 235 clients at 3 MOW program sites**<br>(Including a range of 45-60 clients in Florida, 75-80 in Dallas, Texas, and 100-115 at MOW-SanAntonio, for a grand total of 235 participants) who meet the following eligibility:<br>● Age 66 years or older<br>● Currently on MOW waitlist<br>● Self-report dementia diagnosis or cognitive impairment	Request waiver of Informed Consent<br>-	Request waiver of HIPAA authorization requirement
Client telephone interviews (recorded) 30 minutes	**Sub-group of enrolled clients** participating in the Pilot.<br>Total number interviews: 6-30 clients from 3 MOW program sites (2-10 clients per site)	1. Requesting a waiver of <u>documentation</u> of Informed Consent<br>2. Participant Welcome Letter, Participant Consent Form and Consent Checklist (including consent to audio recording) will be used for consent discussion	n/a (no protected health information is collected)

Informed Consent and HIPAA Authorization, by Subject Group

Caregiver/Emergency Contact interviews (recorded) 30-45 minutes	**Sub-group of caregivers or emergency contacts of enrolled clients** participating in the Pilot.<br><br>Total number interviews: 6-30 caregivers/emergency contacts from 3 MOW program sites (2-10 interviews per site)	1. Requesting a waiver of documentation of Informed Consent<br>2. Caregiver Welcome Letter and Caregiver Consent Form (including consent to audio recording) will be used for consent discussion	n/a (no protected health information is collected)

Brown University IRB Original Approval 12/10/2020
Brown University IRB Amendment Approval 02/25/2021
Original Approval 12/10/2020
Brown University IRB Amendment Approval 05/11/2021
Brown University IRB Amendment Approval 08/19/2021
Brown University IRB Amendment Approval 10/06/2021

ID: | Date: | Research Staff Initials:

Home-delivered meals for persons with dementia: Which model delays nursing home placement?

CONSENT CHECKLIST

Instructions: Complete this checklist as you review the informed consent with the potential participant. Explain to the participant that:

"We are going to go over "Informed Consent" summary sheet that you received with your welcome letter. This summary sheet will explain everything you are being asked to do as part of the study. Feel free to stop me at any time to ask questions. We are going to go slow and review parts of the document as we go to make sure you understand all the pieces of the project. Are you ready? OK…"

1) Does the participant understand the nature of the research and of his/her participation?

☐ **Check #1:** After reviewing the **PURPOSE** on the consent form, say to the participant:

In your own words tell me what this study is about.

If the participant has difficulty, provide simple prompts such as:

- *This study is people's experiences receiving home-delivered meals, can you tell memore about it?*

Notes:

☐ **Check #2:** After reviewing the section, **PROCEDURES**, say to the participant:

In your own words, tell me some of the things you will be asked to do as part of this study.

If the participant has difficulty, provide simple prompts such as:

- *We will contact you by phone, what will we ask you to do?*

Notes:

ID: ____________ Date: ____________ Research Staff Initials: ____________

CONSENT CHECKLIST cont.

2) Does the participant appreciate the consequences of the participation, including personal consequences?

☐ **Check #1:** After reviewing the section, **RISKS**, say:

> *In your own words, tell me some of the risks that might come from being in the study?*

If the participant is unable to spontaneously provide risks that might come from participation in the study, make note of any prompts that are used or sections that are repeated.

Notes:

____

____

____

☐ **Check #2:** After reviewing the section on **BENEFITS** of being in the study say:

> *In your own words, tell me some of the benefits that might come from being in the study?*

If the participant is unable to spontaneously provide benefits that might come from participation in the study, make note of any prompts that are used or sections that are repeated.

Notes:

____

____

____

ID: | Date: | Research Staff Initials:

CONSENT CHECKLIST cont.

3) After reviewing the section, **VOLUNTARY**, does the participant understand participation is voluntary and are they consistently interested in doing the study? *For example:*

☐ **Check #1:** Say: *"Your participation is voluntary, what does that mean to you?"*

☐ **Check #2:** And *"If you change your mind about being in the study, what can you do?"*

Notes:

____

____

____

4) Does the participant show the ability to make a reasoned and consistent choice?

☐ **Check #1:** After reviewing the entire consent form, say: *Do you have any final questions about the study? Does this sound like something you would be willing to take part in?*

Notes:

____

____

____

☐ **Check #2:** Make note throughout the informed consent process and the initial assessment (Baseline) regarding the consistency of their choice to take part in the study.

Notes:

____

____

____

Based on your Capacity to Consent Training: If the participant is unable to perform any of these tasks to your satisfaction, proceed to discontinuing the interview.

Original Approval: 12/10/2020

Attachment: Caregiver Welcome Letter - “Opt In” - Site 1

Date

Dear Care Partner of an Older Adult Receiving Home-Delivered Meals,

You are invited to take part in a telephone interview as part of a Brown University research study. We are asking you to take part because you are listed as an emergency contact for a person who is receiving home-delivered meals.

The goal of this study is to learn about what it is like for care partners of people who receive meals delivered to their homes.

The phone call will take about 30 to 45 minutes. We will ask about what it is like to be a care partner for a person receiving meals at their home. We want to know more about your experience and how the service has impacted your life. We will also ask about any interactions with the person who delivers the meals and how satisfied you are with the services.

This study is paid for by the National Institute on Aging. In this envelope, there is also an Informed Consent Summary Sheet. This sheet gives details on the study. The sheet also has information about the possible risks and benefits of choosing to take part.

To thank you for helping us, we will mail you a $50 pharmacy (for example, CVS or Walgreens) gift card. You will get this giftcard after the telephone interview is done.

If you would like to take part in the study, please call us at xxx-xxx-xxxx. We will tell you about the study and may ask you some questions to see if you qualify.

If you would like more information or to verify this study, please call xxx, Project Coordinator, at xxx-xxx-xxxx. You may also call the Principal Investigator, Dr. Kali Thomas at xxx-xxx-xxxx

Sincerely,

Kali Thomas, PhD
Principal Investigator
Brown University

Brown University IRB Original Approval: 12/10/2020

Attachment: Caregiver Welcome Letter - “Opt Out” - Site 2

Date

Dear Care Partner of an Older Adult Receiving Home-Delivered Meals,

You are invited to take part in a telephone interview as part of a Brown University research study. We are asking you to take part because a client receiving home-delivered meals told us that you were their care partner.

The goal of this study is to learn about what it is like for care partners of people who receive meals delivered to their homes.

The phone call will take about 30 to 45 minutes. We will ask about what it is like to be a care partner for a person receiving meals at their home. We want to know more about your experience and how the service has impacted your life. We will also ask about any interactions with the person who delivers the meals and how satisfied you are with the services.

This study is paid for by the National Institute on Aging. In this envelope, there is also an Informed Consent Summary Sheet. This sheet gives details on the study. The sheet also has information about the possible risks and benefits of choosing to take part.

To thank you for helping us, we will mail you a $50 pharmacy (for example, CVS or Walgreens) gift card. You will get this giftcard after the telephone interview is done.

<u>We are asking you to do one of the following:</u>

1. If you would like to be considered for participation in the study or you would like to know more about this study, please contact us at xxx-xxx-xxxx. We will tell you about the study and may ask you some questions to see if you qualify.

2. If you do not wish to be considered for participation in the study, please return the pre-addressed, postage-paid postcard or call the phone number indicated below.

If we don’t hear from you, we will contact you by telephone in 2-4 weeks from the postmarked date of this letter.

If you would like more information or to verify this study, please call xxx, Project Coordinator, at xxx-xxx-xxxx. You may also call the Principal Investigator, Dr. Kali Thomas at xxx-xxx-xxxx

Sincerely,

Kali Thomas, PhD
Principal Investigator
Brown University

Brown University IRB Original Approval: 12/10/2020

Attachment: Participant Welcome Letter - Reminder

Date

Dear [Name of Program] Client,

We previously reached out to you to see if you were interested in taking part in a telephone interview as part of a Brown University research study.
We are asking you to take part because you are receiving home-delivered meals.

The goal of this study is to learn what it is like for you to receive meals delivered to your home. This study consists of a telephone interview that will take about 30 minutes. We willask you about your experience receiving meals.

This study is paid for by the National Institute on Aging. In this envelope, there is also an Informed Consent Summary Sheet. This sheet gives details on the study. The sheet also has information about the possible risks and benefits of choosing to take part.

To thank you for helping us, we will mail you a $50 pharmacy (for example, CVS or Walgreens) gift card. You will get this giftcard after the telephone interview is done.

If you would like to take part in the study, please contact us at xxx-xxx-xxxx. We will tell you about the study and may ask you some questions to see if you qualify.

If you would like more information or to verify this study, please call xxx, Project Coordinator, at xxx-xxx-xxxx. You may also call the Principal Investigator, Dr. Kali Thomas at xxx-xxx-xxxx.

Sincerely,

Kali Thomas, PhD
Principal Investigator
Brown University

Attachment: Participant Welcome Letter - “Opt Out” - Site 3

Date

Dear [Name of Program] Client,

We are reaching out to you to see if you are interested in taking part in a telephone interview as part of a Brown University research study.
We are asking you to take part because you are receiving home-delivered meals.

The goal of this study is to learn what it is like for you to receive meals delivered to your home. This study consists of a telephone interview that will take about 30 minutes. We willask you about your experience receiving meals.

This study is paid for by the National Institute on Aging. In this envelope, there is also an Informed Consent Summary Sheet. This sheet gives details on the study. The sheet also has information about the possible risks and benefits of choosing to take part.

To thank you for helping us, we will mail you a $50 pharmacy (for example, CVS or Walgreens) gift card. You will get this giftcard after the telephone interview is done.

<u>We are asking you to do one of the following:</u>

1. If you would like to be considered for participation in the study or you would like to know more about this study, please contact us at xxx-xxx-xxxx. We will tell you about the study and may ask you some questions to see if you qualify.

2. If you do not wish to be considered for participation in the study, please return the pre-addressed, postage-paid postcard or call the phone number indicated below.

If we don’t hear from you, we will contact you by telephone in 2-4 weeks from the postmarked date of this letter.

If you would like more information or to verify this study, please call xxx, Project Coordinator, at xxx-xxx-xxxx. You may also call the Principal Investigator, Dr. Kali Thomas at xxx-xxx-xxxx.

Sincerely,

Kali Thomas, PhD
Principal Investigator
Brown University

Attachment: Interview Guide for Caregivers

1) REVIEW CONSENT SHEET WITH PARTICIPANT

As the consent summary sheet stated, the goal of this interview is to understand your experience as a caregiver of an older adult who receives meals delivered to their home. This interview should take no more than 30-45 minutes and you will be given a $50 pharmacy (for example, CVS or Walgreens) gift card for your time. This interview is confidential andnothing you say will be connected with your name, your loved one receiving meals, or the meals that they receive. You have the right to stop the interview at any time.

Do you have any questions about the study procedures as described?

YES.. ANSWER QUESTIONS AND CONTINUE BELOW
NO.. CONTINUE BELOW

Do you agree to participate in this interview?

YES…....................DOCUMENT CONSENT and then PROCEED WITH INTERVIEW
NO........................ CAPTURE REASON(S) for REFUSAL (see below) and READ REFUSAL SCRIPT

REFUSAL SCRIPT - *We want to thank you for considering the interview. We will remove your name from our list of potential participants. As helpful feedback to the investigators conducting this research study, would you be willing to share the reasons you declined participation?*

[WRITE DOWN REASONS FOR REFUSAL VERBATIM]

2) PERMISSION TO RECORD INTERVIEW

Great, I would like to record our conversation so that I can listen closely to what you are telling me. As we discussed earlier, everything that you say will be confidential in that it will not be connected with your name or meal program. The recording will be saved in a secure location and will be destroyed after the completion of this study. **[START RECORDER UNLESS PARTICIPANT OBJECTS]**

3) CONDUCT INTERVIEW QUESTIONS

1. Tell me what it's like for [enter name of participant] to get mealdeliveries.
   Probes:
   If someone helps, who is it and how?
   What do they think about the person who brings the meals?
   Does [name of participant] look forward to receiving the meals?
   What does [name of participant] like/not like about receiving the meals?
   What else?

2. Tell me about when and how the meals are prepared and eaten.
   Probes:
   How much of the meal is eaten. What more can you say about that?
   How the participant prepares meals.
   When [name of participant] does not receive meals, how do they get their food? For example, on weekends? Or other times?
   What else?

3. Think about before meals were coming to [name of participant's] home and then what it was like after they started to get meals:
   Probes:
   How do the meals compare to what [name of participant] used to eat?

How do you think [name of participant's] health has changed since receiving meals?
Tell me more about this.

4. We've heard about how meals affected [name of participant], and now I'd like to know whether and how receiving meals has affected YOUR life. Tell me how you feel your situation may have changed since the [name of participant] is receiving meals.
   Probe about worry, health, stress, mood, isolation.
   Probe about any positive effects (follow-up prompts could be: conversation, feedback about participant's general well-being, feeling connected, a bright spot in the day, anything else?)

5. What suggestions do you have for how the meal program could be improved or better tailored to those with dementia?
   Probe about communication with participant, communication with caregiver, assistance.

6. Would you recommend that a friend or relative with dementia sign up for these meals, if offered?
   Probes:
   What makes you want to recommend these meal deliveries?
   What things make you feel like not recommending these meals?

Attachment: Interview Guide for Participants

(This interview will be conducted over the telephone approximately one month after initiation of meals. Prior to the start of the interview, participants will be administered the Telephone Interview of Cognitive Status (TICS-M) to assess the participant's level of cognitive impairment. Following that, the interview questions below will be asked. The interview guide may be altered and refined during piloting. We understand that IRB amendments will need to be filed should any changes or the process change.)

1) Review Consent Sheet with Participant (use the attached Capacity to Consent Checklist when reviewing the form**)**

"We are going to go over "Informed Consent" summary sheet that you received with your welcome letter. This summary sheet will explain everything you are being asked to do as part of the study. Feel free to stop me at any time to ask questions. We are going to go slow and review parts of the document as we go to make sure you understand all the pieces of the project. Are you ready? OK…"

- Does the participant understand the nature of the research and of his/her participation?
  - Check #1. After reviewing the PURPOSE on the consent form, say to the participant: "*In your own words tell me what this study is about.*"
    - If the participant has difficulty, provide simple prompts such as:
  - Check #2: After reviewing the section, PROCEDURES, say to the participant: "*In your own words, tell me some of the things you will be asked to do as part of this study."*
- Does the participant appreciate the consequences of the participation, includingpersonal consequences?
  - Check #1: After reviewing the section, RISKS, say: *In your own words, tell me some of the risks that might come from being in the study?*
    - If the participant is unable to spontaneously provide risks that might come from participation in the study, make note of any prompts that are used or sections that are repeated.
  - Check #2: After reviewing the section on BENEFITS of being in the study say: *In your own words, tell me some of the benefits that might come from being in the study?*
- After reviewing the section, VOLUNTARY, does the participant understand participation is voluntary and are they consistently interested in doing the study? *For example:*
  - Check #1: Say: *Do you understand that your participation is voluntary, it is your decision?*
  - Check #2: And *"If you change your mind about being in the study, what can you do?*
- Does the participant show the ability to make a reasoned and consistent choice?
  - Check #1: After reviewing the entire consent form, say: *Do you have any final questions about the study? Does this sound like something you would be willing to take part in?*
  - Check #2: Make note throughout the informed consent process and the initial assessment (Baseline) regarding the consistency of their choice to take part in the study.

Do you agree to participate in this interview?

YES........................DOCUMENT CONSENT and then PROCEED WITH INTERVIEW
NO........................ CAPTURE REASON(S) for REFUSAL (see below) and READ REFUSAL SCRIPT

REFUSAL SCRIPT - *We want to thank you for considering the interview. We will remove your name from our list of potential participants. As helpful feedback to the investigators conducting this research study, would you be willing to share the reasons you declined participation?*

[*WRITE DOWN REASONS FOR REFUSAL VERBATIM*]

2) PERMISSION TO RECORD INTERVIEW

Great, I would like to record our conversation so that I can listen closely to what you are telling me. As we discussed earlier, everything that you say will be confidential in that it will not be connected with your name or meal program. The recording will be saved in a secure location and will be destroyed after the completion of this study. **[START RECORDER UNLESS PARTICIPANT OBJECTS]**

3) COMPLETE TELEPHONE INTERVIEW FOR COGNITIVE STATUS (TICS-M)

Telephone Interview for Cognitive Status (TICS-M)

Orientation

1.	(i) What day of the week is it?	Day	�
	(ii) What is today's date?	Date	�
		Month	�
		Year	�
	(iii) What season are we in?	Season	�
2.	What is your age?	Age:	�
3.	What is your telephone number? (Code+ number)		�

Registration/Free Recall

4.	(i)I'm going to read you a list of 10 words. Please listen carefully and try to remember them. When I am done, tell me as many as you can in any order. Ready?	Cabin	�
		Pipe	�
		Elephant	�
		Chest	�
	Now, tell me all the words you can remember	Silk	�
		Theatre	�
		Watch	�
		Whip	�
		Pillow	�
		Giant	�

Attention/Calculation

5.	(i) Please take 7 away from 100	93	�
	(ii) Now continue to take 7 away from what you have left over until I ask you to stop.	86	�
		79	�
		72	�
		65	�
6.	Please count backwards from 20 to 1	No mistakes	�

Comprehension, Semantic and Recent Memory

7. What do people usually use to cut paper?	Scissors	�
8. What is the prickly green plant found in the desert?	Cactus	�
9. Who is the president now?	Correct surname	�
10. Who is the vice president now?	Correct surname	�
11. What is the opposite of east?	West	�

Language/Repetition

12. Please say this 'Methodist Episcopal'	Exactly right	�

Delayed Recall

13. Please repeat the list of 10 words I read earlier	Cabin	�
	Pipe	�
	Elephant	�
	Chest	�
	Silk	�
	Theatre	�
	Watch	�
	Whip	�
	Pillow	�
	Giant	�
		□ maximum of 39

4. QUESTIONS ABOUT MEAL DELIVERIES

We would like to ask some questions about your meals and want you to know that there are no right or wrong answers.

1. First of all, tell me if meals are delivered to your home or not.
   a. If no, ask how they get their food at home. (This response will cue the interviewer on howto approach the interview)

2. What is it like to get meal deliveries?
   a. Tell me a little about it: What usually happens? How does the meal get inside your home?
      i. (Probe about preparation, assistance, if any)
   b. What do you think about the person who brings the meals?
      i. (Probe about whether they look forward to seeing that person, what they do to receive it)
      ii. What is the person like? (if all negative or positive, ask about the opposite. For example, you've mentioned a lot of nice things about the person; is there anything you don't like so much about them?)
   c. How much of the meal do you eat?
      i. (Probe for more information)
   d. What do you like about the food/meals you receive?
      i. (Probe about smell, appearance, taste, variety, anything else?)
   e. Tell me about what you don't like so much about these meals. Remember, there are no right or wrong answers and your answers help us know how people feel about the meals.
   f. How could the meals be improved?

3. When you don't receive meals, how do you get your food?
      i. (Probe about weekends, other times of the day)

4. Think about before meals were coming to your home and then what it was like after you started to get meals:
   a. How are these meals different or the same from what you used to eat?
   b. How do you think your health has changed?
   c. Tell me how you think your mood may be different since receiving meals.

d. In general, how do you feel getting meals delivered to your home?

5. Would you recommend that a friend or relative sign up for these meals, if offered?
    a. What makes you want to recommend these meal deliveries?
    b. What things make you feel like not recommending these meals?
    c. What else do you think we need to know about what receiving meals is like for you?

Original Approval: 12/10/2020

BROWN UNIVERSITY
PARTICIPANT CONSENT FOR RESEARCH PARTICIPATION

Home-delivered meals for persons with dementia:
Which model delays nursing home placement?

Version 2, August 18, 2021

KEY INFORMATION:

You are invited to take part in a Brown University study. Your participation is voluntary.

- PURPOSE: The study is about people's experiences receiving home-delivered meals.
- PROCEDURES: You will be asked to participate in a telephone interview about your experiences receiving meals delivered to your home.
- TIME INVOLVED: The study will take about 30 minutes of your time.
- COMPENSATION: You will receive a $50 pharmacy (for example, CVS or Walgreens) gift card for your time.
- RISKS: We will ask you questions about your experience receivinghome-delivered meals. Some of these questions may make you feel uncomfortable, or bring up unpleasant feelings or memories.
- BENEFITS: While you may not directly benefit from being in this study, your experiences will help us better understand older adults' experiences receiving home-delivered meals.

1. **Researcher(s):**

Kali Thomas, Principal Investigator; xxx-xxx-xxxx; xx_xxx@brown.edu

2. **What is this study about?**

The purpose of the study is to understand the experiences of people receiving home-delivered meals. We also want to understand what helps people who receive home-delivered meals stay living in the community.

You are being asked to be in this study because you receive meals delivered to your home.

3. **What will I be asked to do?**

You are being asked to participate in a telephone interview about your experiences receiving meals delivered to your home. You will be asked questions like: "What is it like to get meal deliveries?" "When you don't receive meals, how do you get your food?" "In general, how do you feel getting meals delivered to your home?"

The interview also includes a brief memory exercise.

Brown University IRB Original Approval: 12/10/2020
Brown University IRB Original Approval: 12/10/2020
Brown University IRB Amendment Approval 02/25/2021
Brown University IRB Amendment Approval 05/11/2021
Brown University IRB Amendment Approval 08/19/2021
Brown University IRB Amendment Approval 10/06/2021

We will be recording our conversation during the interview. We will use a notebook for notes and a digital recorder.

Your participation in this study should take about 30 minutes.

4. Will I be paid?

A $50 pharmacy (for example, CVS or Walgreens) gift card will be mailed to your home within one week of completing the interview.

5. What are the risks?

We will ask you questions about your experience receiving home-delivered meals. Some of these questions may make you feel uncomfortable, or bring up unpleasant feelings or memories. If there are any questions that you are uncomfortable answering, you do not need to answer them. You are free to stop the interview and recording at any time.

6. What are the benefits?

While you may not directly benefit from being in this study, your participation will help us better understand older adults' experiences receiving home-delivered meals.

7. How will my information be protected?

Any information we collect that can be used to identify you will be carefully protected. Our research staff are trained in protecting confidential information, and we have security measures in place to ensure the integrity and confidentiality of all data.

All data we collect (such as recordings or notes we take) will be stored in a locked filing cabinet in the researcher's locked office. All electronic files we create using the data will be secured with passcodes for protection.

We will be recording our conversation during the interview. We will use a notebook for notes and a digital recorder. We will keep this information confidential. Only researchers working on this study will have access to the data. We will destroy the recording after the study is over.

Brown University staff sometimes review studies like this one to make sure they are being done safely and correctly. If a review of this study takes place, your records may be examined. The reviewers will protect your confidentiality.

Certification of Confidentiality: To help us protect your privacy, we have obtained a Certificate of Confidentiality from the National Institutes of Health. The researchers can use this Certificate to legally refuse to disclose information that may identify you in any federal, state, or local civil, criminal, administrative, legislative, or other proceedings, for example, if there is a court subpoena. The researchers will use the Certificate to resist any demands for information that would identify you, except as explained below.

Brown University IRB Original Approval: 12/10/2020
Brown University IRB Amendment Approval 02/25/2021
Original Approval: 12/10/2020
Brown University IRB Amendment Approval 05/11/2021
Brown University IRB Amendment Approval 08/19/2021
Brown University IRB Amendment Approval 10/06/2021

The Certificate cannot be used to resist a demand for information from personnel of the United States federal or state government agency sponsoring the project and that will be used for auditing or program evaluation of agency funded projects or for information that must be disclosed in order to meet the requirements of the federal Food and Drug Administration (FDA). You should understand that a Certificate of Confidentiality does not prevent you or a member of your family from voluntarily releasing information about yourself or your involvement in this research. If an insurer, medical care provider, or other person obtains your written consent to receive research information, then the researchers will not use the Certificate to withhold that information.

The Certificate of Confidentiality will not be used to prevent disclosure to state or local authorities of such as child abuse and neglect, or harm to self or others.

8. What if I want to stop?

You do not have to be in this study if you do not want to be. Even if you decide to be interviewed, you can change your mind and stop at any time.

If you refuse to participate or leave the study, your current or future relationship with Brown University and your meals program will not be affected.

9. Who can I talk to if I have questions about this study?

If you have any questions about your participation in this study, you can call XXX, Project Coordinator, at xxx-xxx-xxxx or by email at xxxx@brown.edu or Dr. Kali Thomas, Principal Investigator, xxx-xxx-xxxx or xx_xxx@brown.edu

10. Who can I talk to if I have questions about my rights as a participant?

If you have questions about your rights as a research participant, you can contact Brown University's Human Research Protection Program at 401-863-3050 or email them at IRB@Brown.edu.

Brown University IRB Original Approval: 12/10/2020
Brown University IRB Amendment Approval 02/25/2021
Brown University IRB Amendment Approval 05/11/2021
Brown University IRB Amendment Approval 08/19/2021
Brown University IRB Amendment Approval 10/06/2021

BROWN UNIVERSITY
CAREGIVER CONSENT FOR RESEARCH PARTICIPATION

Home-delivered meals for persons with dementia:
Which model delays nursing home placement?

Version 2, August 18, 2021

KEY INFORMATION:

You are invited to take part in a Brown University study. Your participation is voluntary.

- PURPOSE: The study is about people's experiences receiving home-delivered meals
- PROCEDURES: You will be asked to participate in a telephone interview
- TIME INVOLVED: The study will take 30-45 minutes of your time.
- COMPENSATION: You will receive a $50 pharmacy (for example, CVS or Walgreens) gift card compensation for your time.
- RISKS: In this study, we will ask you questions about your experience as a care partner of someone who receives home-delivered meals. Some of these questions may make you feel uncomfortable, or bring up unpleasant feelings or memories.
- BENEFITS: While you may not directly benefit from being in this study, your experiences will help us better understand older adults' experiences receiving home-delivered meals.

1. Researcher(s):
Kali Thomas, Principal Investigator; xxx-xxx-xxxx; xx_xxx@brown.edu

2. What is this study about?
The purpose of the study is to understand the experiences of people receiving home-delivered meals. We also want to understand the experiences of care partners of people who receive meals delivered to their homes.

You are being asked to be in this study because you are listed as an emergency contact of a person who receives home-delivered meals.

3. What will I be asked to do?

You are being asked to participate in a telephone interview about your experience as a care partner of a person who is receiving meals delivered to their home. We will also ask questions about your interactions (if any) with the person who delivers meals, how meal service impacts your life, and your satisfaction, or dissatisfaction, with meal services.

We will be recording our conversation during the interview. We will use a notebook for notes and a digital recorder.

Brown University IRB Original Approval: 12/10/2020
Brown University IRB Amendment Approval 02/25/2021
Brown University IRB Amendment Approval 05/11/2021
Brown University IRB Amendment Approval 08/19/2021
Brown University IRB Amendment Approval 10/06/2021
Brown University IRB Amendment Approval 02/25/2021
Brown University IRB Amendment Approval 05/11/2021

Your participation in this study may last up to 45 minutes.

4. Will I be paid?

A $50 pharmacy (for example, CVS or Walgreens) gift card will be mailed to your home within one week of completing the interview.

5. What are the risks?

We will ask you questions about your experience as a care partner for a person who receives home-delivered meals. Some of these questions may make you feel uncomfortable, or bring up unpleasant feelings or memories. If there are any questions that you are uncomfortable answering, you do not need to answer them. You can stop the interview and recording at any time.

6. What are the benefits?

While you may not directly benefit from being in this research study, your experiences will help us better understand older adults' experiences receiving home-delivered meals.

7. How will my information be protected?

Any information we collect that can be used to identify you will be carefully protected. Our research staff are trained in protecting confidential information, and we have security measures in place to ensure the integrity and confidentiality of all data.

All data we collect (such as recordings or notes we take) will be stored in a locked filing cabinet in the researcher's locked office. All electronic files we create using the data will be secured with passcodes for protection.

We will be recording our conversation during the interview. We will use a notebook for notes and a digital recorder. We will keep this information confidential. Only researchers working on this study will have access to the data. Any results published using this data will not include your name or any other identifiable information. We will destroy the recording of our interview after the study is over.

Brown University staff sometimes review studies like this one to make sure they are being done safely and correctly. If a review of this study takes place, your records may be examined. The reviewers will protect your confidentiality.

Certification of Confidentiality: To help us protect your privacy, we have obtained a Certificate of Confidentiality from the National Institutes of Health. The researchers can use this Certificate to legally refuse to disclose information that may identify you in any federal, state, or local civil, criminal, administrative, legislative, or other proceedings, for example, if there is a court subpoena. The researchers will use the Certificate to resist any demands for information that would identify you, except as explained below.

Brown University IRB Original Approval: 12/10/2020
Brown University IRB Amendment Approval 02/25/2021
Brown University IRB Original Approval: 12/10/2020
Brown University IRB Amendment Approval 05/11/2021
Brown University IRB Amendment Approval 08/19/2021
Brown University IRB Amendment Approval 10/06/2021

The Certificate cannot be used to resist a demand for information from personnel of the United States federal or state government agency sponsoring the project and that will be used for auditing or program evaluation of agency funded projects or for information that must be disclosed in order to meet the requirements of the federal Food and Drug Administration (FDA). You should understand that a Certificate of Confidentiality does not prevent you or a member of your family from voluntarily releasing information about yourself or your involvement in this research. If an insurer, medical care provider, or other person obtains your written consent to receive research information, then the researchers will not use the Certificate to withhold that information.

The Certificate of Confidentiality will not be used to prevent disclosure to state or local authorities of such as child abuse and neglect, or harm to self or others.

8. What if I want to stop?

You do not have to be in this study if you do not want to be. Even if you decide to be interviewed, you can change your mind and stop at any time.

If you refuse to participate or leave the study, your current or future relationship with Brown University and the meals program will not be affected.

9. Who can I talk to if I have questions about this study?

If you have any questions about your participation in this study, you can call XXX, Project Coordinator, at xxx-xxx-xxxx or by email at xxxx@brown.edu or Dr. Kali Thomas, Principal Investigator, xxx-xxx-xxxx or xx_xxx@brown.edu

10. Who can I talk to if I have questions about my rights as a participant?

If you have questions about your rights as a research participant, you can contact Brown University's Human Research Protection Program at 401-863-3050 or email them at IRB@Brown.edu.

Brown University IRB Original Approval: 12/10/2020

Neighborly

Trusted Senior Services Provider
since 1966

Kali S. Thomas, PhD, MA
Associate Professor of Health Services, Policy and Practice
Center for Gerontology and Health Care Research
Brown University School of Public Health
Box G-S121(6)
Providence, RI 02912

Dear Dr. Thomas,

Meals on Wheels Pinellas County (Neighborly Care Network) is pleased to write this letter of strong organizational support to partner with you and Brown University for your proposed research entitled "Home-delivered meals for persons with dementia: Which model delays nursing home placement?"

We understand the overall goal of this research is to evaluate two types of meal delivery and assess which type of meal is best for our homebound clients with dementia by allowing them to live longer in their communities. The project will be conducted with Meals on Wheels America and include multiple member programs across the United States; one of these program sites will be Meals on Wheels Pinellas County.

Our program serves 2900 clients per year and currently has a waiting list of 900 individuals, 26% of whom have been told by a healthcare professional that they suffer from memory loss, cognitive impairment, any type of dementia, or Alzheimer's disease. We are eager to serve this project as the research will have a direct impact on our operations, the healthcare system and ultimately, the lives of our clients. We are increasingly contracting with healthcare providers and payers who are interested in offering nutrition services to their homebound, food insecure patients, many of whom have dementia. Having the evidence from this project will inform those discussions. We are familiar with the previous research conducted by Brown University under your direction and are excited to participate in this study. We are confident this project will be greatly beneficial to our services and our clients.

We are writing to confirm our institutional support for your research. My team and I have already provided feedback on the initial study design and look forward to providing additional input that reflects the most important issues to our clients, families, drivers, and our program. We appreciate the resources provided by the grant to support our efforts, and we are committed to providing the necessary support to accomplish the aims of this proposal, including access to our long and growing waiting list to recruit participants. We look forward to working with you over the five years, during the pilot and full trial phases. We will participate in

13945 Evergreen Ave,. Clearwater, FL 33762 **Phone**: (727) 573-9444

Brown University IRB Original Approval 12/10/2020
Brown University IRB Amendment Approval 02/25/2021
Brown University IRB Amendment Approval 05/11/2021
Brown University IRB Amendment Approval 08/19/2021
Brown University IRB Amendment Approval 10/06/2021

the initial training designed for our program, participate in monthly meetings, host the research team on their site visits and identify one client and one client caregiver to serve on the Stakeholder Advisory Panel. In addition, we will identify a staff member to provide administrative support, enroll participants, track relevant data, and coordinate data access to your research team for project implementation. Our staff are looking forward to working with you and your team at Brown University and Meals on Wheels America during the pilot, active phase of project implementation, as well as the preparation of presentations and informational materials resulting from this project.

We are confident that this research will positively impact our clients with dementia and inform the conversations we and other home-delivered meals programs are having with healthcare. Thank you for the opportunity to be part of this important work aimed at advancing the evidence around home-delivered meals in an effort to improve the lives of our nation's older adult population.

Sincerely,

Anita Frankhauser, MS, RDN, LDN
Director of Nutrition and Licensed Dietitian
Neighborly Care Network
13945 Evergreen Ave
Clearwater, FL 33762

wn University IRB Original Approval 12/10/2020
Brown University IRB Amendment Approval 02/25/2021
Original Approval: 12/10/2020
Brown University IRB Amendment Approval 05/11/2021
Brown University IRB Amendment Approval 08/19/2021
Brown University IRB Amendment Approval 10/06/2021

Association

Meals on Wheels
Hospice & Palliative Care

October 22, 2020

OFFICERS

Mary Frances Bellma n, Chairman
Eugenia King , Chairman Elect
Max Lamont, Treasurer
Uzzie Routman , Secretary

DIRECTORS

Haftda Baad
Jay Barlow
Bob Best
Jan Hart Black
Jordan Brainerd
Cathy Bryan, RN
Rebecca P Casey
Michelle Chase
Julie D. Clark
Cat her ine A. Corrigan
Levi H. Davis
Daniel DeMarco, MD
Effie Dennison
Kevin Eden
Betsy Eiseman
Kathleen Gibson
Benjamin Isgur
Zakiyah A. Johnson
Kirsten Kerrigan
Bob Krakow
Alison Draper Lolley
Louise Marsh
Pressley H. Peters
Carolyn Rathjen
Kersten Rettig
Camisha L. Simmons
Denice Swift
Alok Vaish
Andres Villareal
Jim Walton, DO
CiCi Weinmann
Paula Will s

HONORARY BOARD TRUSTEES

Deborah Cannon
Sara Fraser Crismon
Robert Ted Enloe, Ill
Philip C. Henderson
Lyda Hill
Jerry P Knippa
Lynn McBee
Jan Mcclendon
Teresa Haggerty Parravano
Daniel Polter, MD
Lucy S. Polter
Ru st E. Reid
Shirley Tobolowsky (Mrs. Edwin)
Joe Nathan Wright

PRESIDENT AND CEO

Katherine Krause

Kali S. Thomas, PhD, MA
Associate Professor of Health Services, Policy and
Practice Brown University School of Public Health Box
G-Sl21(6)
Providence, RI 02912

Dear Dr. Thomas,

The Visiting Nurses Association of Texas is pleased to write this letter of strong organizational support to partner with you and Brown University for your funded research study entitled "Home-delivered meals for persons with dementia: Which model delays nursing home placement?"

We understand the overall goal of this research is to evaluate two types of meal delivery and assess which type of meal is best for our homebound senior clients with dementia by allowing them to live longer in their communities. The project will be conducted with Meals on Wheels America and we will participate as a pilot site for this study.

Our program served 6,363 unduplicated clients this past year and currently has a waiting list of 289 seniors. We are eager to serve on this project as the research will have a direct impact on our operations, the healthcare system and ultimately, the lives of our clients. We are increasingly contracting with healthcare providers and payers who are interested in offering nutrition services to their homebound, food insecure patients, many of whom have dementia. Having the evidence from this project will inform those discussions. We have worked closely with you and your research team on previous projects and are excited to work with you on this new endeavor. We are confident this project will be greatly beneficial to our services and our clients.

We are writing to confirm our institutional support for your research and willingness to serve on your stakeholder advisory panel. My team and I have already provided feedback on the initial study design and look forward to providing additional input that reflect the most important issues to our clients, families, drivers, and our program. We appreciate the resources provided by the grant to support our efforts, and we are committed to providing the necessary support to accomplish the aims of this project, including access to our long and growing waiting list to recruit participants.

Brown University IRB Amendment Approval 01/25/2021
Brown University IRB Original Approval: 12/10/2020
Brown University IRB Amendment Approval 05/11/2021
Brown University IRB Amendment Approval 08/19/2021
Brown University IRB Amendment Approval 10/06/2021

We will participate in developing training materials and Manual of Operating Procedures, participate in team meetings, and identify one client and one client family member to serve on the stakeholder advisory panel.-In addition , we will identify a staff member to provide administrative support, enroll clients in one of two types of meal programs, assist with identification of possible participants for interviews, and coordinate data access to your research team for project implementation. Our staff are looking forward to working with you and your team at Brown University and Meals on Wheels America during this pilot project.

We are confident that this research and that which will follow will positively impact our clients with dementia and inform the conversations we and other home-delivered meals programs are having with healthcare. Thank you for the opportunity to be part of this important work aimed at advancing the evidence around home-delivered meals in an effort to improve the lives of our nation' s older adult population.

With gratitude,

Katherine Krause
President and Chief Executive Officer

Brown University IRB Original Approval 12/10/2020
Brown University IRB Amendment Approval 02/25/2021
Brown University IRB Amendment Approval 05/11/2021
Brown University IRB Amendment Approval 08/19/2021
Brown University IRB Amendment Approval 02/23/2021
Brown University IRB Amendment Approval 10/06/2021
Brown University IRB Original Approval 12/10/2020
Brown University IRB Amendment Approval 05/11/2021

MEALS on WHEELS AMERICA

October 23, 2020

Kali S. Thomas, PhD, MA
Associate Professor, Health Services, Policy and Practice
Brown University School of Public Health
Box G-S121(6)
Providence, RI 02912

Dear Dr. Thomas,

On behalf of Meals on Wheels America, it is with great pleasure that I am committing to our continued partnership with Brown University in support of the proposed pilot research, "Home-delivered meals for persons with dementia: Which model delays nursing home placement?" Meals on Wheels America has been committed to building the evidence base that demonstrates the impact and value of home-delivered meals for a long time. Brown University has been a critical partner in that endeavor.

The need for home-delivered meals is great and growing but the resources to meet this need are woefully inadequate and millions of seniors are going without these basic services leading to decline in health and well-being and often, institutionalization. As Meals on Wheels America has identified healthcare partnerships as its core strategic priority in order to drive resources to the provision of Meals on Wheels services to a far greater number of seniors who can benefit from our services, including those with dementia, the abilityto demonstrate impact in terms of both healthcare outcomes and need for high-cost healthcare services is essential.

The proposed pilot study will provide a much-needed opportunity to build out this evidence base in areas that are of greatest interest to prospective healthcare partners, including what mode of meal delivery works best for patients with dementia.

Meals on Wheels America is the leading national voice for the nation's estimated 5,000 community-based Meals on Wheels providers that are committed to serving seniors struggling with hunger and isolation. As a national organization, we advocate, provide education and training, serve as a convener, conduct research, and much more to support the ability of the network to extend its reach and impact. In its continuing partnership with Brown University, Meals on Wheels America wiIl provide the following organizational support:

- Meals on Wheels America has secured support from two programs located in Florida and Texas (with waiting lists that include individuals with dementia) to participate in this pilot project. These programs have collectively agreed to enroll 40 clients and to serve on the Stakeholder Advisory Panel. We are committed to recruiting additional programs, if needed, depending on what is learned during the pilot phase of the project.
- Meals on Wheels America will contract with and manage our partnership with TRIO

Brown University IRB Original Approval: 12/10/2020
Brown University IRB Amendment Approval 02/25/2021
Original Approval: 12/10/2020
Brown University IRB Amendment Approval 05/11/2021
Brown University IRB Amendment Approval 08/19/2021
Brown University IRB Amendment Approval 10/06/2021

Community Meals to bi-weekly deliver the ten frozen meals to 20 clients randomly assigned to that intervention group.

- We will work with Meals on Wheels programs to recruit clients with dementia and one family members to serve on the Stakeholder Advisory Panel
- Meals on Wheels America will provide project management to our participating member programs and will work with them directly throughout the pilot study project to ensure its success.
- We will co-host training of programs and monthly conference calls with programs and research team.
- We will leverage our communications team and channels to disseminate information about this important study and its findings.

Throughout the development of this pilot project, the MOW program directors and I have been involved in the study design and provided important feedback about feasibility and outcomes. I look forward to continuing to work with you and your team in this capacity and engage with you throughout the award by sharing Meals on Wheels experiences and perspectives from working with our clientele.

I am writing to confirm our institutional support of the proposed pilot project and to leverage the resources of Meals on Wheels America to fulfill our obligations as described above. In my role as Chief Strategy and Impact Officer, I will serve as the primary point of contact for this mission-critical work to ensure that our responsibilities are executed in accordance with our agreement with Brown University. In addition to my role as an integral member of the research team, I will also serve on the Stakeholder Advisory Panel alongside Uche Akobundu, PhD. We have established a strong partnership and have worked closely together in the development of this proposed research project.

I am confident that this research will be essential in our efforts to partner with healthcare for the delivery of Meals on Wheels services. As a result, we will be better positioned to ensure that no senior in this country is hungry or isolated.

Sincerely,

Lucy Theilheimer
Chief Strategy and Impact Officer
Direct: 571.339.1601 Cell: 703.850.3865
1550 Crystal Drive, Suite 1004
Arlington, VA 22202

Brown University IRB Original Approval: 12/10/2020
Brown University IRB Amendment Approval 02/25/2021
Original Approval: 12/10/2020
Brown University IRB Amendment Approval 05/11/2021
Brown University IRB Amendment Approval 08/19/2021
Brown University IRB Amendment Approval 10/06/2021

INSTRUCTIONS FOR COMPLETING THE DATA USE AGREEMENT (DUA) FORM CMS-R-0235

(AGREEMENT FOR USE OF CENTERS FOR MEDICARE & MEDICAID SERVICES (CMS) DATA CONTAINING INDIVIDUAL IDENTIFIERS)

This agreement must be executed prior to the disclosure of data from CMS' Systems of Records to ensure that the disclosure will comply with the requirements of the Privacy Act, the Privacy Rule and CMS data release policies. It must be completed prior to the release of, or access to, specified data files containing protected health information and individual identifiers.

Directions for the completion of the agreement follow:

Before completing the DUA, please note the language contained in this agreement cannot be altered in any form.

- First paragraph, enter the Requestor's Organization Name.
- Section #1, enter the Requestor's Organization Name.
- Section #4 enter the Study and/or Project Name and CMS contract number if applicable for which the file(s) will be used.
- Section #5 should delineate the files and years the Requestor is requesting. Specific file names should be completed. If these are unknown, you may contact a CMS representative to obtain the correct names The System of Record (SOR) should be completed by the CMS contact or Project Officer. The SOR is the source system the data came from.
- Section #6, complete by entering the Study/Project's anticipated date of completion.
- Section #12 will be completed by the User.
- Section #16 is to be completed by Requestor.
- Section #17, enter the Custodian Name, Company/Organization, Address, Phone Number (including area code), and E-Mail Address (if applicable). The Custodian of files is defined as that person who will have actual possession of and responsibility for the data files. **This section should be completed even if the Custodian and Requestor are the same.** This section will be completed by Custodian.
- Section #18 will be completed by a CMS representative.
- Section #19 should be completed if your study is funded by one or more other Federal Agencies. The Federal Agency name (other than CMS) should be entered in the blank. The Federal Project Officer should complete and sign the remaining portions of this section. If this does not apply, leave blank.
- Sections #20a AND 20b will be completed by a CMS representative.
- Addendum, CMS-R-0235A, should be completed when additional custodians outside the requesting organization will be accessing CMS identifiable data.

Once the DUA is received and reviewed for privacy and policy issues, a completed and signed copy will be sent to the Requestor and CMS Project Officer, if applicable, for their files.

wn University IRB Original Approval: 12/10/2020
wn University IRB Amendment Approval [illegible]/2021
Original Approval: 12/10/2020
Brown University IRB Amendment Approval 10/06/2021
Brown University IRB Amendment Approval 05/11/2021
Brown University IRB Amendment Approval 08/19/2021

Form Approved
OMB No. 0938-0734

DATA USE AGREEMENT

DUA # ____________

(AGREEMENT FOR USE OF CENTERS FOR MEDICARE & MEDICAID SERVICES (CMS) DATA CONTAINING INDIVIDUAL IDENTIFIERS)

CMS agrees to provide the User with data that reside in a CMS Privacy Act System of Records as identified in this Agreement. In exchange, the User agrees to pay any applicable fees; the User agrees to use the data only for purposes that support the User's study, research or project referenced in this Agreement, which has been determined by CMS to provide assistance to CMS in monitoring, managing and improving the Medicare and Medicaid programs or the services provided to beneficiaries; and the User agrees to ensure the integrity, security, and confidentiality of the data by complying with the terms of this Agreement and applicable law, including the Privacy Act and the Health Insurance Portability and Accountability Act. In order to secure data that reside in a CMS Privacy Act System of Records; in order to ensure the integrity, security, and confidentiality of information maintained by the CMS; and to permit appropriate disclosure and use of such data as permitted by law, CMS and Brown University *(Requestor)* enter into this agreement to comply with the following specific paragraphs.

1. This Agreement is by and between the Centers for Medicare & Medicaid Services (CMS), a component of the U.S. Department of Health and Human Services (HHS), and Brown University *(Requestor)*, hereinafter termed "User."

2. This Agreement addresses the conditions under which CMS will disclose and the User will obtain, use, reuse and disclose the CMS data file(s) specified in section 5 and/or any derivative file(s) that contain direct individual identifiers or elements that can be used in concert with other information to identify individuals. This Agreement supersedes any and all agreements between the parties with respect to the use of data from the files specified in section 5 and preempts and overrides any instructions, directions, agreements, or other understanding in or pertaining to any grant award or other prior communication from the Department of Health and Human Services or any of its components with respect to the data specified herein. Further, the terms of this Agreement can be changed only by a written modification to this Agreement or by the parties adopting a new agreement. The parties agree further that instructions or interpretations issued to the User concerning this Agreement or the data specified herein, shall not be valid unless issued in writing by the CMS point-of-contact or the CMS signatory to this Agreement shown in section 20.

3. The parties mutually agree that CMS retains all ownership rights to the data file(s) referred to in this Agreement, and that the User does not obtain any right, title, or interest in any of the data furnished by CMS.

4. The User represents, and in furnishing the data file(s) specified in section 5 CMS relies upon such representation, that such data file(s) will be used solely for the following purpose(s).

Name of Study/Project
Home-delivered meals for persons with dementia: Which model delays nursing home placement?

CMS Contract No. *(If applicable)*

The User represents further that the facts and statements made in any study or research protocol or project plan submitted to CMS for each purpose are complete and accurate. Further, the User represents that said study protocol(s) or project plans, that have been approved by CMS or other appropriate entity as CMS may determine, represent the total use(s) to which the data file(s) specified in section 5 will be put.

The User agrees not to disclose, use or reuse the data covered by this agreement except as specified in an Attachment to this Agreement or except as CMS shall authorize in writing or as otherwise required by law, sell, rent, lease, loan, or otherwise grant access to the data covered by this Agreement. The User affirms that the requested data is the minimum necessary to achieve the purposes stated in this section. The User agrees that;

wn University IRB Original Approval: 12/10/2020
wn University IRB Amendment Approval [illegible]
Brown University IRB Amendment Approval 05/11/2021
Brown University IRB Amendment Approval 08/19/2021
Brown University IRB Amendment Approval 10/06/2021

wn University IRB Original Approval: 12/10/2020

within the User organization and the organizations of its agents, access to the data covered by this Agreement

wn University IRB Original Approval: 12/10/2020
wn University IRB Amendment Approval 02/25/2021
Brown University IRB Amendment Approval 10/06/2021
Brown University IRB Amendment Approval 05/11/2021
Brown University IRB Amendment Approval 08/19/2021

shall be limited to the minimum amount of data and minimum number of individuals necessary to achieve the purpose stated in this section (i.e., individual's access to the data will be on a need-to-know basis).

5. The following CMS data file(s) is/are covered under this Agreement.

File	Years(s)	System of Record

6. The parties mutually agree that the aforesaid files(s) (and/or any derivative file(s)), including those files that directly identify individuals or that directly identify bidding firms and/or such firms' proprietary, confidential or specific bidding information, and those files that can be used in concert with other information to identify individuals, may be retained by the User until 1 year, hereinafter known as the "Retention Date." The User agrees to notify CMS within 30 days of the completion of the purpose specified in section 4 if the purpose is completed before the aforementioned retention date. Upon such notice or retention date, whichever occurs sooner, the User agrees to destroy such data. The User agrees to destroy and send written certification of the destruction of the files to CMS within 30 days. The User agrees not to retain CMS files or any parts thereof, after the aforementioned file(s) are destroyed unless the appropriate Systems Manager or the person designated in section 20 of this Agreement grants written authorization. The User acknowledges that the date is not contingent upon action by CMS.

   The Agreement may be terminated by either party at any time for any reason upon 30 days written notice. Upon notice of termination by User, CMS will cease releasing data from the file(s) to the User under this Agreement and will notify the User to destroy such data file(s). Sections 3, 4, 6, 8, 9, 10, 11, 13, 14 and 15 shall survive termination of this Agreement.

7. The User agrees to establish appropriate administrative, technical, and physical safeguards to protect the confidentiality of the data and to prevent unauthorized use or access to it.The safeguards shall provide a level and scope of security that is not less than the level and scope of security requirements established by the Office of Management and Budget (OMB) in OMB Circular No. A-130, Appendix III--Security of Federal Automated Information Systems **(http://www.whitehouse.gov/omb/circulars/a130/a130.html)** as well as Federal Information Processing Standard 200 entitled "Minimum Security Requirements for Federal Information and Information Systems" **(http://csrc.nist.gov/publications/fips/fips200/FIPS-200-final-march.pdf);** and, Special Publication 800-53 "Recommended Security Controls for Federal Information Systems" **(http://csrc.nist.gov/publications/nistpubs/800-53-Rev2/sp800-53-rev2-final.pdf)**. The User acknowledges that the use of unsecured telecommunications, including the Internet, to transmit individually identifiable, bidder identifiable or deducible information derived from the file(s) specified in section 5 is prohibited. Further, the User agrees that the data must not be physically moved, transmitted or disclosed in any way from or by the site indicated in section 17 without written approval from CMS unless such movement, transmission or disclosure is required by a law.

8. The User agrees to grant access to the data to the authorized representatives of CMS or DHHS Office of the Inspector General at the site indicated in section 17 for the purpose of inspecting to confirm compliance with the terms of this agreement.

wn University IRB Original Approval: 12/10/2020
wn University IRB Amendment Approval: 02/25/2021
Original Approval: 12/10/2020
Brown University IRB Amendment Approval 10/06/2021
Brown University IRB Amendment Approval 05/11/2021
Brown University IRB Amendment Approval 08/19/2021

9. The User agrees not to disclose direct findings, listings, or information derived from the file(s) specified in section 5, with or without direct identifiers, if such findings, listings, or information can, by themselves or in combination with other data, be used to deduce an individual's identity. Examples of such data elements include, but are not limited to geographic location, age if > 89, sex, diagnosis and procedure, admission/discharge date(s), or date of death.

   The User agrees that any use of CMS data in the creation of any document (manuscript, table, chart, study, report, etc.) concerning the purpose specified in section 4 (regardless of whether the report or other writing expressly refers to such purpose, to CMS, or to the files specified in section 5 or any data derived from such files) must adhere to CMS' current cell size suppression policy. **This policy stipulates that no cell (e.g. admittances, discharges, patients, services) 10 or less may be displayed.** Also, no use of percentages or other mathematical formulas may be used if they result in the display of a cell 10 or less. By signing this Agreement you hereby agree to abide by these rules and, therefore, will not be required to submit any written documents for CMS review. If you are unsure if you meet the above criteria, you may submit your written products for CMS review. CMS agrees to make a determination about approval and to notify the user within 4 to 6 weeks after receipt of findings. CMS may withhold approval for publication only if it determines that the format in which data are presented may result in identification of individual beneficiaries.

10. The User agrees that, absent express written authorization from the appropriate System Manager or the person designated in section 20 of this Agreement to do so, the User shall not attempt to link records included in the file(s) specified in section 5 to any other individually identifiable source of information. This includes attempts to link the data to other CMS data file(s). A protocol that includes the linkage of specific files that has been approved in accordance with section 4 constitutes express authorization from CMS to link files as described in the protocol.

11. The User understands and agrees that they may not reuse original or derivative data file(s) without prior written approval from the appropriate System Manager or the person designated in section 20 of this Agreement.

12. The parties mutually agree that the following specified Attachments are part of this Agreement:

    Signature addenda, DUA Attachment B

13. The User agrees that in the event CMS determines or has a reasonable belief that the User has made or may have made a use, reuse or disclosure of the aforesaid file(s) that is not authorized by this Agreement or another written authorization from the appropriate System Manager or the person designated in section 20 of this Agreement, CMS, at its sole discretion, may require the User to: (a) promptly investigate and report to CMS the User's determinations regarding any alleged or actual unauthorized use, reuse or disclosure, (b) promptly resolve any problems identified by the investigation; (c) if requested by CMS, submit a formal response to an allegation of unauthorized use, reuse or disclosure; (d) if requested by CMS, submit a corrective action plan with steps designed to prevent any future unauthorized uses, reuses or disclosures; and (e) if requested by CMS, return data files to CMS or destroy the data files it received from CMS under this agreement. The User understands that as a result of CMS's determination or reasonable belief that unauthorized uses, reuses or disclosures have taken place, CMS may refuse to release further CMS data to the User for a period of time to be determined by CMS.

    The User agrees to report any breach of personally identifiable information (PII) from the CMS data file(s), loss of these data or disclosure to any unauthorized persons to the CMS Action Desk by telephone at (410) 786 -2580 or by e-mail notification at **cms_it_service_desk@cms.hhs.gov** within one hour and to cooperate fully in the federal security incident process. While CMS retains all ownership rights to the data file(s), as outlined above, the User shall bear the cost and liability for any breaches of PII from the data file(s) while they are entrusted to the User. Furthermore, if CMS determines that the risk of harm requires notification of affected individual persons of the security breach and/or other remedies, the User agrees to carry out these remedies without cost to CMS.

14. The User hereby acknowledges that criminal penalties under §1106(a) of the Social Security Act (42 U.S.C. § 1306(a)), including a fine not exceeding $10,000 or imprisonment not exceeding 5 years, or both, may apply to disclosures of information that are covered by § 1106 and that are not authorized by regulation or by Federal law. The User further acknowledges that criminal penalties under the Privacy Act (5 U.S.C. § 552a(i) (3)) may apply if it is determined that the Requestor or Custodian, or any individual employed or affiliated therewith, knowingly and willfully obtained the file(s) under false pretenses. Any person found to have violated sec. (i)(3) of the Privacy Act shall be guilty of a misdemeanor and fined not more than $5,000. Finally, the User acknowledges that criminal penalties may be imposed under 18 U.S.C. § 641 if it is determined that the User, or any individual employed or affiliated therewith, has taken or converted to his own use data file(s), or received the file(s) knowing that they were stolen or converted. Under such circumstances, they shall be fined under Title 18 or imprisoned not more than 10 years, or both; but if the value of such property does not exceed the sum of $1,000, they shall be fined under Title 18 or imprisoned not more than 1 year, or both.

15. By signing this Agreement, the User agrees to abide by all provisions set out in this Agreement and acknowledges having received notice of potential criminal or administrative penalties for violation of the terms of the Agreement.

16. On behalf of the User the undersigned individual hereby attests that he or she is authorized to legally bind the User to the terms this Agreement and agrees to all the terms specified herein.

Name and Title of User *(typed or printed)*		
David A. Savitz, PhD, Professor and Chair of Epidemiology, School of Public Health		
Company/Organization<br>Brown University		
Street Address<br>121 S. Main Street, 2nd floor, Box G-S121-2		
City<br>Providence	State<br>RI	ZIP Code<br>02912
Office Telephone *(Include Area Code)*<br>401-863-9060	E-Mail Address *(If applicable)*<br>cgchr@brown.edu	
Signature	Date	

17. The parties mutually agree that the following named individual is designated as Custodian of the file(s) on behalf of the User and will be the person responsible for the observance of all conditions of use and for establishment and maintenance of security arrangements as specified in this Agreement to prevent unauthorized use. The User agrees to notify CMS within fifteen (15) days of any change of custodianship. The parties mutually agree that CMS may disapprove the appointment of a custodian or may require the appointment of a new custodian at any time.

The Custodian hereby acknowledges his/her appointment as Custodian of the aforesaid file(s) on behalf of the User, and agrees to comply with all of the provisions of this Agreement on behalf of the User.

Name of Custodian *(typed or printed)*		
Jeffrey Hiris, MA		
Company/Organization<br>Brown University		
Street Address<br>121 S. Main Street, 6th floor, Box G-S121-6		
City<br>Providence	State<br>RI	ZIP Code<br>02912
Office Telephone *(Include Area Code)*<br>401-863-3317	E-Mail Address *(If applicable)*<br>Jeffrey_Hiris@brown.edu	
Signature	Date	

18. The disclosure provision(s) that allows the discretionary release of CMS data for the purpose(s) stated in section 4 follow(s). (To be completed by CMS staff.) ____

19. On behalf of____________________________________the undersigned individual hereby acknowledges that the aforesaid Federal agency sponsors or otherwise supports the User's request for and use of CMS data, agrees to support CMS in ensuring that the User maintains and uses CMS's data in accordance with the terms of this Agreement, and agrees further to make no statement to the User concerning the interpretation of the terms of this Agreement and to refer all questions of such interpretation or compliance with the terms of this Agreement to the CMS official named in section 20 (or to his or her successor).

Typed or Printed Name	Title of Federal Representative
Signature	Date
Office Telephone *(Include Area Code)*	E-Mail Address *(If applicable)*

20. The parties mutually agree that the following named individual will be designated as point-of-contact for the Agreement on behalf of CMS.

On behalf of CMS the undersigned individual hereby attests that he or she is authorized to enter into this Agreement and agrees to all the terms specified herein.

Name of CMS Representative *(typed or printed)*		
Title/Component		
Street Address		Mail Stop
City	State	ZIP Code
Office Telephone *(Include Area Code)*	E-Mail Address *(If applicable)*	
A. Signature of CMS Representative		Date
B. Concur/Nonconcur — Signature of CMS System Manager or Business Owner		Date
Concur/Nonconcur — Signature of CMS System Manager or Business Owner		Date
Concur/Nonconcur — Signature of CMS System Manager or Business Owner		Date

According to the Paperwork Reduction Act of 1995, no persons are required to respond to a collection of information unless it displays a valid OMB control number. The valid OMB control number for this information collection is 0938-0734. The time required to complete this information collection is estimated to average 30 minutes per response, including the time to review instructions, search existing data resources, gather the data nee ded, and complete and review the information collection. If you have any comments concerning the accuracy of the time estimate(s) or suggestions for improving this form, please write to: CMS, 7500 Security Boulevard, Attn: [illegible] 21244-1850.

[illegible]

wn University IRB Original Approval: 12/10/2020
wn University IRB Amendment Approval 02/26/2021
wn University IRB Amendment Approval 05/11/2021
Brown University IRB Amendment Approval 10/06/2021
Brown University IRB Amendment Approval 08/19/2021

Research Identifiable Files

DUA #: ________________

Attachment B

This attachment supplements the above-referenced Data Use Agreement (DUA) between the Centers for Medicare and Medicaid Services and the User (as set forth in the DUA). Upon execution by both parties, to the extent this Attachment is inconsistent with any terms in the DUA, this Attachment modifies and overrides the DUA.

A-1. Use of Data: Users may disseminate research findings on providers or suppliers (including individual physicians) using original or derived information from the files specified in Section 5 of the DUA provided all findings are limited to patient de-identified data that conform with the HIPAA Privacy Rule's definition of de-identified data at 45 CFR 164.514(b).

A-2. Disclosure of Findings: Nothing in the DUA, including but not limited to Section 9, prohibits Users from discussing or reporting on specific providers or suppliers (including individual physicians) in a manner consistent with A-1.

____

For: Centers for Medicare & Medicaid Services

____

For: DUA Data Requestor

Original Approval: 12/10/2020

Centers for Medicare & Medicaid Services

Executive Summary for Research Identifiable Data

Version 4/2015

For CMS Use Only
Privacy Board Approval Date:<br>Part D Approval Date:

**DUA User name and title** (see Item 16 of DUA)	David A. Savitz, PhD, Professor and Chair of Epidemiology, School of Public Health
**Requesting Organization**[1] (see Item 1 of DUA)	Brown University
**Type of Organization**	Academic
**Study PI** (if different from DUA User)	Kali Thomas, PhD
**Study Title**	Home-delivered meals for persons with dementia: Which model delays nursing home placement?
**Funding Source**	NIA, R61AG070170

Executive Summary | Dissemination and Reporting of Findings | Data Management Plan | Project Staff | Collaborator Checklist

EXECUTIVE SUMMARY

1. **Study Overview**
   Please describe your study objectives and aims.
   Click here to enter text.

2. **How have you ensured that your data request includes the minimum amount of data necessary to achieve your research objectives?**
   2.1. **Please describe how this cohort will meet minimum data necessary.** *(Include estimated cohort size. Refer to your cost invoice.)*
   Click here to enter text.

   2.2. **List the CMS data files and years being requested at this time and provide justification for how each will be used in the analysis.** *If requesting reuse of data, include the DUA # to be reused. The list of files should match Item #5 of DUA.*
   2.2.1. Medicare (claims and enrollment) or Medicaid (claims and enrollment)
   Click here to enter text.
   2.2.2. Part D event data (if using in study)
   Click here to enter text.
   2.2.3. Part D characteristics files (if using in study)
   Click here to enter text.
   2.2.4. Assessment data (if using in study)
   Click here to enter text.

   2.3. **If this study will require further years of CMS data that are not yet available for request, please list those CMS data files and years that will be required for the *entire scope of your study*** *(Note: Approval of data files for years that are not yet available will NOT be granted at this time, the information included here will simply provide CMS with an overview of your study).*
   Click here to enter text.

---

[1] Throughout this document, "organization" can be interpreted as the company, agency, or group or team within a company, depending on which makes more sense in context with the research study for which CMS data files are being used. For example, large companies may defer to a CMS data file inventory for just their team; whereas smaller companies may keep a single CMS data file inventory for the entire company.

2.4. **Please list any non-identifiable or non-CMS files you are planning to use in conjunction with the above files for your analysis.** (e.g. Provider of Services (POS) file, AMA Physician Master file, etc.)
Click here to enter text.

3. **You are requesting Research Identifiable Files (RIF). Why can't Limited Data Set (LDS) files be used for this study?**
Assessment data are not available as LDS.

4. **Is it feasible to obtain individual level authorization from Medicare/Medicaid beneficiaries for your research? Explain.**
No, all of the data were previously collected for non-research purposes. We will not have contact with individuals.

5. **If you intend on requesting the National Death Index segment of the Master Beneficiary Summary File, please complete the NDI Supplement.**
□ YES, I've included the NDI Supplement □ NO, I'm not requesting the NDI

6. **If this research project is funded by a commercial entity, the (primary) lead investigator attests that they will limit data sharing with the funding entity to aggregated analytic results and will retain the right to independently prepare publications of the study results.** □ I attest

**Signature of (Primary) Lead Investigator**	**Date**

DISSEMINATION AND REPORTING OF FINDINGS

From the CMS DUA, "The User agrees that any use of CMS data in the creation of any document (manuscript, table, chart, study, report, etc.) concerning the purpose specified in section 4 (regardless of whether the report or other writing expressly refers to such purpose, to CMS, or to the files specified in section 5 or any data derived from such files) must adhere to CMS' current cell size suppression policy. **This policy stipulates that no cell (e.g. admittances, discharges, patients, services) 10 or less may be displayed.** Also, no use of percentages or other mathematical formulas may be used if they result in the display of a cell 10 or less."
□ I agree.

Please describe your plans for disseminating the findings from your analysis, including specific media through which you will report results.
We will disseminate findings through peer-reviewed venues such as abstracts for presentations at national meetings (i.e., conferences at the Gerontological Society of America and AcademyHealth's annual meetings) and manuscripts for publication in journals such as the Journal of the American Geriatrics Society, and Health Services Research. We intend to use this DUA to support preliminary work for future studies and doctoral theses, in turn leading to peer-reviewed publications. If doctoral work formalizes, the student will request his/her own DUA.

Brown University IRB Original Approval: 12/10/2020
Brown University IRB Amendment Approval 02/25/2021
Brown University IRB Amendment Approval 05/11/2021
Brown University IRB Amendment Approval 08/19/2021
Brown University IRB Amendment Approval 10/06/2021

PROJECT STAFF

This section specifically identifies the project staff, organization, and the role of individuals in this project. The requestor and custodian should be named in this section at a minimum.

**1.Name & Title of Requestor /User**	Bess Marcus, PhD, Dean, School of Public Health
**Organization**	Brown University
**Role in this Study**	Requestor, University Signatory
**Will this individual have access to raw data, analytic files, or output with cell sizes less than 11?**	NO.<br>□ YES, this individual will be directly supervised by DUA signatory, [Name] .<br>□ YES, this individual has signed the DUA.

**2.Name & Title of Custodian**	Jeff Hiris, MA, IT Director
**Organization**	Brown University
**Role in this Study**	Data Custodian
**Will this individual have access to raw data, analytic files, or output with cell sizes less than 11?**	□ NO.<br>□ YES, this individual will be directly supervised by DUA signatory, [Name] .<br>YES, this individual has signed the DUA.

**3.Name & Title**	Julie Lima, PhD, Investigator
**Organization**	Brown University
**Role in this Study**	Data acquisition and compliance
**Will this individual have access to raw data, analytic files, or output with cell sizes less than 11?**	□ NO.<br>□ YES, this individual will be directly supervised by DUA signatory, [Name] .<br>YES, this individual has signed the DUA or signature addendum.

**4.Name & Title**	
**Organization**	
**Role in this Study**	
**Will this individual have access to raw data, analytic files, or output with cell sizes less than 11?**	□ NO.<br>□ YES, this individual will be directly supervised by DUA signatory, [Name] .<br>□ YES, this individual has signed the DUA or signature addendum.

*** If more individuals need to be added to this section, please copy and paste above fields.*

DATA MANAGEMENT PLAN[2]

Please reference the [Data Management Plan Guidelines], [Data Management Plan Evaluation Guide], [Collaborator Checklist], and/or the [FAQ document] for more information on completing this section. These materials are found under the Executive Summary section of the New Study Requesting Data page of the website.

For research studies involving researchers from another organization that will have access to RIF or non-identifiable files, please refer to the Collaborator Checklist for guidance and considerations to include in the Data Management Plan.

For collaborating organizations that will be receiving a physical copy of the CMS data files, a full Data Management Plan should be completed by the collaborating organization.

1. PHYSICAL POSSESSION AND STORAGE OF CMS DATA FILES

1.1. Who will have the main responsibility for organizing, storing, and archiving the data? Please provide name(s) and job title(s).

Jeffrey R Hiris, IT Director

1.2. Describe how your organization maintains a current inventory of CMS datafiles.

An Excel spreadsheet is used to track all incoming and outgoing media. It records the media identity insofar as it is labeled, date and direction of transfer, file system location of the files copied from the media, and briefly describes the data. Decrypted versions of the raw data are stored in the same file system location, or below it. Raw data is stored in directories with a regular naming convention which clear identifies the source of the data. No master inventory is maintained of all derivative datasets, however all CMS data is processed in a similar fashion. 1) Raw data is read using whatever accompanying code or formatting is provided by a program stored in a single standard location by the Custodian; 2) one of a small number of programmers then applies code to standardize this raw data, such code being stored in a small number of protected locations; and 3) all standardized datasets are then stored in a small number of specific file system locations. Analytic datasets built pursuant to a specific DUA are then stored in a DUA-specific analysis location within the file system. Programs which manipulate that data clearly identify the DUA, EDGN, and/or any other identifying information provided when the data us received. Second stage programs which further standardize the data clearing identify the source data upon which they operate.

1.3. Describe how your organization binds all members (i.e., organizations, individual staff) of research teams to specific privacy and security rules in using CMS data files.

All staff and organizations which handle CMS data covered by a DUA are identified on that DUA and if required have signed the appropriate amendment which binds them to CMS requirements. Center staff and any other data users are required to sign internal confidentiality agreements covering all Center work with sensitive data.

This internal confidentiality agreement is found at the back of the Center's Handbook of Computing and Research Manual and is signed by all Brown and non-Brown personnel working on data stored at the Center. It includes the following information:

---

[2] Note that CMS is specifically asking for reference to written policies and procedures related to your organization's administrative, technical and physical safeguards. If policies and procedures have not been developed, please explain any ongoing activities your organization is taking to document and make them available to staff. Organizations selected for DPSP reviews will be asked to provide copies of written policies and procedures. Please note that an explanation of the process is not sufficient.

Brown University IRB Original Approval: 12/10/2020
Brown University IRB Amendment Approval 02/25/2021
Brown University IRB Amendment Approval 05/11/2021
Brown University IRB Amendment Approval 08/19/2021
Brown University IRB Amendment Approval 10/06/2021

Data Protection & Security Agreement

I,____, through my involvement with and work on approved research projects at the Center for Gerontology and Health Care Research (the Center) will have access to sensitive data. While Brown University and the Center have taken every precaution to ensure the security of these data, I understand it is my responsibility to comply with the Center's data security protocol. I understand that access to project data is restricted to individuals who are authorized to work on specific research projects and I agree to fulfill my responsibilities in accordance with the following guidelines:

1) All data to which any user is granted access by the Center remain the property of Brown, insofar as Brown can assert ownership.

2) Data will be used on Brown equipment only, except as displayed by supported remote access mechanisms on equipment operated in accordance with Center policy.

3) Only fully de-identified non-CMS person-level data is permitted to be removed from the Center's protected network, physical perimeter, and local systems. Partially-identified data, which has been appropriately encrypted, can be removed only when authorized in advance in writing by the data owner [as determined by an approved Data Use Agreement (DUA)] and data custodian.

4) Partially-identified data may only be moved to a Brown-owned desktop system for processing purposes. Data are not to be stored on local hard drives.

5) No attempt will be made to correlate any Brown data with "real" individuals except as approved in advance by the Brown IRB.

6) Any security breach which occurs on any hardware or software under the control of any user shall be reported to the Center's Director of Operations, the data owner (as determined by an approved DUA), and the system manager as soon as the user becomes aware of it. The user may request any of the above to contact the others, but it remains the user's responsibility to ensure that each of the above arenotified.

7) All datasets built using data covered by a Data Use Agreement will be erased when such Data Use Agreement terminates, upon notification by the PI for a project or the data custodian that such termination has occurred. Output which displays substantial parts of such data will likewise be erased.

I understand that violation of these guidelines may result in disciplinary and/or legal action.

1.4. Provide details about whom and how your organization will notify CMS of any project staffing changes.
Principal Investigators will notify relevant staff changes to our DUA Manager and Project Contact (presently Julie Lima) as soon as they become aware of staff additions or terminations in the scope of this DUA. Dr. Lima will then notify CMS of the removal or addition of personnel through the procedures currently outlined at https://www.cms.gov/Research-Statistics-Data-and-Systems/Files-for-Order/Data-Disclosures-Data-Agreements/DUA_-_Contact_Changes.html
For example, a contact update request will be filed in a timely manner reflecting the change and for personnel additions (as opposed to removals), will be accompanied by any necessary signatures. Directly supervised staff are not required to be reported to CMS, although the PI is required to approve any access to CMS data for use under a particular DUA Similarly, we are not required to notify CMS of staffing changes not involved with the handling of sensitive data.

1.5. Describe your organization's training programs that are used to educate staff on how to protect CMS data files.
Brown staff are trained under the Brown CITI certification system in the handling of human subjects data. CMS data is not considered different from other sensitive human subjects data. The Center is small enough that staff handling sensitive data are trained internally and informally on the proper handling of CMS data files.
Staff handling sensitive data are required to read our Center's Handbook of Computing and Research and sign a confidentiality agreement which indicates they understand the need to protect sensitive data, and the technical requirements of maintaining such protections. In addition, our DUA Manager and Project Contact (currently Julie Lima) gives a Center-wide talk 2-3 times yearly to outline ongoing policies and procedures for working with confidential data at the Center.

1.6. Explain the infrastructure (facilities, hardware, software, other) that will secure the CMS datafiles.

a. Physical security for the servers housing sensitive data is provided by a combination of Brown card-access controls and physically secured space within a common server room. The network switching infrastructure is maintained by Brown and our network is protected by ACLs which limit external access to services and protocols which are unencrypted or otherwise too sensitive to traverse an external network. Power and cooling integrity is provided by Brown facilities management, including generator power in the event of a loss of external power. Hardware support for the Center's servers is provided by the Center and by Brown central IT(CIS).
b. Data is currently stored on file servers and is protected by ACLs leveraging group membership to provide an effective baseline security model. Access by client Windows systems is controlled by the user's membership in this group structure. While the underlying security model is DAC, users are not granted Control access to any files nor directories. Data is routinely moved between file servers as a part of routine systems support activities, and ACLs controlling access are automatically rewritten to their baseline state as a part of any suchcopying.
c. While the Center does host a variety of modern services in support of its mission, including Web servers, users are strictly limited in how they can interact with such services. The casual dissemination of Web content to the public in particular is not possible.

Access to the Center's internal networks is protected by multiple layers of firewalls and network access control lists. The University as a whole is committed to a multi-year plan to improve network security by moving sensitive data resources into non-routable internal networks. Given the complexity of all the Center's operations and the need to maintain a coherent network infrastructure this is a multi-year process.

Remote access to any data, regardless of sensitivity, is in general limited to remote access via Windows terminal servers. (Trusted staff are permitted to make VPN connections using Center-owned and maintained client systems.) Such servers are housed in the same data center as the Windows servers and operate in the same security context. Some few staff handling less sensitive data do make remote connections to their usual desktop systems, which is permitted but discouraged.

1.7. Describe the policies and procedures regarding the physical possession and storage of CMS datafiles.
The Brown CIS Acceptable Use Policy (defined in http://www.brown.edu/cis/policy/aup.php) requires that all users of Brown computing resources are limited to those resources for which they have appropriate authorization and are to use those resources in a manner consistent with their authorization. CMS identifiable data files fall into the category of PIHI data and their appropriate use is defined by general by the Policies and Procedures Relating to Research Privacy of the Office of the Provost (http://www.brown.edu/Administration/Provost/policies/rpp.html). In a general sense, CMS data files may be broken into three classes, namely identifiable data, Limited Data Sets (as defined in the context of the above Policy), and de-identified data with small cell suppression applied. Functionally, CMS data may be classified as media received from CMS and local computer files built from the data contained on such media. All received CMS data file are stored in the Center's locked section of the machine room. Normally, they are either in that secure location or in the physical possession of the data custodian, for example when received from a courier service. Similarly, working copies of data files containing PIHI or LDS data are only stored on servers within that

secure locked location. LDS datasets may be analyzed locally on workstations within our firewalled network, but are not stored on such local systems. All data acquired under a DUA which is identifiable, protected, embargoed, or otherwise sensitive is never stored on user-controlled removable media of any type. As a standard Disaster Recovery procedure such data is stored in an encrypted form on removable backup media. As outlined in the accompanying justification letter to this DUA request, one complete set of tapes will be held externally for Disaster Recovery (DR) purposes in a secure vault maintained by Iron Mountain at their Rhode Island offsite facility located in Chepachet, RI, 02814. Others are maintained in the same secure environment as our servers and secured in the same fashion (that is, with limited access by systems staff, behind two locked doors, and with card access controls).

Why: in the event of a catastrophic failure of the Center's computing infrastructure it is essential that a reasonably recent backup copy of all data and software systems be maintained. Note that internally we maintain multiple copies of basically all data and systems on non-removable media, replicated between various storage systems. Such replication is secured by the use of local disks, firewalls, and/or encryption. However, in the event of a catastrophic environmental failure (e.g. a water leak leading to a roof collapse over the server room near in time to a similar disaster in the DR replicate server room), or a malicious software attack leading to data loss (e.g., a ransomware attack), an offline backup would be essential to ensure recovery.

Physical safeguards: all backup tapes except for those stored offsite for DR purposes are stored within the same physical environment as the Center's main servers. Any retired media are physically crushed before being removed from the server room before being shipped out as e-waste for shredding and recycling. (Our current media are Linear Tape Open (LTO) tapes which cannot be effectively de-gaussed). The one offsite copy will be picked up by an Iron Mountain courier for storage in their facility, such copy to consist of a box of tapes in a container secured with a locking tag. After the first copy has been transferred a monthly swap will occur via the Iron Mountain courier. Given the media are encrypted there seems minimal operational risk in allowing staff working for our Director of Operations to hold the monthly box of media in an external safe for the brief times between removal from the server room and acceptance by/receipt from the Iron Mountaincourier.

1.8. Explain your organization’s system or process to track the status and roles of the researchteam.
As each PI is individually responsible for their research project, as matter of Center Policy each PI is required to track their staff allocations and responsibilities. Staff are required to sign a certification of their percent effort on each project. From a computer operations perspective, each PI is required to identify any unique security groups or contexts within their research, and to identify team members who should be granted access to specific resources. (For example, any project with PIHI and LDS data would have a more secure location and more restricted group for PIHI access, a separate space and group for LDS access.) Given the small number of PIs within the Center, this has been controlled informally using e-mail as the control system and the local authorization databases as the system of record, but given CMS’s requirement of auditable controls, we are developing a paper records system to document rights granted to staff members. Note that as a matter of cost control much of the CMS data provided to the Center is handled by a small number of programmers who work across all CMS projects on initial data preparation and removal of PHI identifiers. (Thus, the role of these core programmers with respect to the CMS PIHI data for any individual research project is not specifically tracked since they have the same duties with respect to all projects.) Research team members who are not a member of this small group of programmers will be granted limited access to data as needed for their duties, or to project-specific location for project-specific PIHI.

1.9. Describe your organization’s physical and technical safeguards used to protect CMS data files (including physical access and logical access to the files).
As mentioned above, physical access to sensitive systems is controlled by physical locks behind Brown card-access controls. Access is limited to systems management staff, and as a part of University operations Brown

life safety personnel and other necessary University exceptions. Technical safeguards are primarily provided by the use of access controls on data stored on the core servers. Software interfaces which can access the data are limited in number, scope, and connectivity to other systems, and tightly controlled by the Center. In particular, unencrypted transmission of any Center data outside the secure internal networks is blocked insofar as technically possible. Ultimately, the security and integrity of all Center data depends upon the diligence and integrity of the staff handling the data.

Physical security for the servers housing sensitive data is provided by a combination of Brown card-access controls and physically secured space within a common server room. The network switching infrastructure is maintained by Brown and our network is protected by ACLs which limit external access to services and protocols which are unencrypted or otherwise too sensitive to traverse an external network. Power and cooling integrity is provided by Brown facilities management, including generator power in the event of a loss of external power. Hardware support for the Center's servers is provided by the Center in concert with Central IT. The Center is exploring the feasibility of utilizing hosted servers in Brown secure central data center to augment servers in our local machine room in order to better leverage central IT resources, such servers and services to remain under the technical ownership of the Center.

Physical safeguards for disaster recovery copies on removable media: all backup tapes except for those stored offsite for DR purposes are stored within the same physical environment as the Center's main servers. Any retired media are physically crushed before being removed from the server room before being shipped out as e-waste for shredding and recycling. (Our current media are Linear Tape Open (LTO) tapes which cannot be effectively de-gaussed). The one offsite copy will be picked up by an Iron Mountain courier for storage in their facility, such copy to consist of a box of tapes in a container secured with a locking tag. After the first copy has been transferred a monthly swap will occur via the Iron Mountain courier. Given the media are encrypted there seems minimal operational risk in allowing staff working for our Director of Operations to hold the monthly box of media in an external safe for the brief times between removal from the server room and acceptance by/receipt from the Iron Mountain courier.

2. DATA SHARING, ELECTRONIC TRANSMISSION, DISTRIBUTION

2.1. Describe your organization's policies and procedures regarding the sharing, transmission, and distribution of CMS data files.

It is our policy that data is received under a data use agreement with CMS and cooperatively the PI acts as local functional data owner and the Center as technical data owner. Any sharing, transmission, or distribution is strictly controlled by the requirements of the Data use Agreement signed with CMS.

It is a violation of Brown Policy to move PIHI or LDS data out from the secure systems to any less secure system, and as a matter of Center Policy no portable systems nor removable media are authorized for the storage of identifiable data except as part of our Disaster Recovery plan as outlined in sections 1.7 and 1.8 above and in the accompanying justification letter.

In the exceedingly rare case of the transfer of non-identifiable data, the encrypted data file may be transferred using encrypted network protocols, notably https, with password protection for the transfer. Note that separate passwords are used for encryption and transfer, and both are shipped via other media and methods.

General Brown Policies regarding appropriate data use are binding upon all faculty and staff of Brown University. In particular:

Ultimately the “Policy on the Handling of Brown Restricted Information” (https://it.brown.edu/computing-policies/policy-handling-brown-restricted-information) defines in general terms the need to protect human subjects data, which would include any data received from CMS under a DUA.

That in turn references “Policies and Procedures Relating to Research Privacy” (http://www.brown.edu/Administration/Provost/policies/rpp.html) which defines in general terms the requirements for defining and controlling access to PHI data.

Somewhat tangentially, data received from CMS under a DUA may be viewed as the property of CMS, even if not personal health information. The use of such data is covered in general terms by the “Acceptable Use Policy” (http://www.brown.edu/cis/policy/aup.php) which requires compliance with the requirements of the data’s owner.

Within the Center’s context, we have a Handbook of Computing and Research that articulates procedures for the handling of CMS data. However, note that ultimately a DUA is an agreement between the PI and CMS; the Center is a component of Brown and cannot change Brown Policy nor make new Policy. It can augment Brown Policy regarding internal operations, such as the mechanics of data transfers.

At present, all external data transfers of sensitive data are handled by the Data Custodian for the Center.

2.2. If your organization employs a data tracking system, pleasedescribe.
There is no industry definition for this term, but insofar as it is commonly used, we do not employ a data tracking system. It is a violation of Brown Policy to move PIHI or LDS data out from the secure systems to any less secure system and as a matter of Center Policy no portable systems nor removable media are authorized for the storage of identifiable data except as part of our Disaster Recovery plan as outlined in sections 1.7 and 1.8 above and in the accompanying justification letter. Note that following best practices, Center computer security is implemented in a concentric fashion with all access to secured locations where PIHI data should be stored limited to a small number of staff persons. Most research team members have no access right to PIHI data, and limited access to LDS data as appropriate to their role.
The Center has undertaken internal discussion in this regard, and is in the process of determining how best to track CMS (and other sensitive data) within our systems. Given current limits to technical controls that can be applied when Investigators and staff are working on numerous projects with different DUAs, ultimately any such process is dependent upon staff adhering to Policy requiring such tracking. It is therefore unclear whether tracking individual data items is useful; we currently employ an implicit classification scheme that aligns security descriptors with storage locations. Aligning that scheme with DUA requirements and in turn requiring that files covered by a DUA be stored in the place that classifies them as belonging to that DUA may thus be a more scalable approach than inventorying and tracking datasets individually.

The Center is working on a documented version of these procedures. This is therefore explanatory, not required procedure.

In general, note that the Center’s computer operations are generally based within Windows, based on access to individual files. No database technology is used to store CMS data for general use. Files are organized into trees and read access to a tree from a specific root downwards is granted to specific groups of users. Write access is granted to a subset of those users at and below certain nodes in the file tree. Given the needs to users to create their own files and the need for consistent security, it is considered neither feasible nor supportable to manage access to individual files within the file tree except asabove.

Informally, all incoming data is stored by the Custodian within a set of locations to which only a subset of the professional programming staff. Specific code is responsible for removing primary identifiers from incoming data, and otherwise cleaning it and standardizing it, before it is placed into different “base file” locations. Read access to this partially de-identified data is then granted to additional analysts, PIs, and staff, upon receipt of an appropriate confidentiality form.

Finally, each DUA (or other type of project, e.g. contract) has one or more file trees dedicated to its work. Access to these is granted by systems management staff upon receipt of a request from the PI. Note that such requests also inform the grant of access to base files, e.g. claims data.

Therefore, ultimately the classification system is based upon location of a file within the file trees; essentially, the top-level directory identifies the classification of the files within. DUA-specific analytic files are clearly identified as being part of that project. Incoming and base files of various types, e.g. claims and MDS assessments, are identified as such, but their creation and destruction is the responsibility of the Custodian and, rarely, core programming staff under the direction of a PI.

Since data is stored within files, access could be tracked via various audit and alarm mechanisms. To date we have no requirement to provide such tracking, and to be honest, it would be hard to effectively track access in the sense of reduce and analyze access patterns given funding limits.

Note that it is a matter of Center policy that all data be stored in accordance with the above guidelines, at least for current research. We do know, from the inception of a DUA or other project, which spaces users are required to store files for that project in. We do not centrally track the name, size, or other specifics of the files that are developed in the course of a project, any more than we centrally track the names of documents and spreadsheets produced. The PI has complete visibility over all files within the space for a given DUA and is responsible for certifying the destruction of all datasets within said space as part of DUA termination.

2.3. Describe the policies and procedures your organization has developed for the physical removal, transport and transmission of CMS data files.
The Policy Relating to Research Privacy from the Office of the Provost, referenced above, covers all handling of PIHI, and requires that it be kept confidential. Within our Center, in all cases, whether data is inbound or outbound, no sensitive data in transmitted nor received unless encrypted. Encryption is generally applied which includes the AES-256 standard, although other strong encryption may be applied under specific circumstances. (For example, while not relevant to the handling of CMS data, SSL encryption is commonly employed for websites which must display PIHI or other PII.) Encryption keys are transmitted separately, if possible using alternative media and/or channel.

2.4. Explain how your organization will tailor and restrict data access privileges based on an individual’s role on the research team.
Depending upon the needs of the research team, one or more security project groupings are used. Each project grouping allows for staff to be assigned read-only or read-write access to project files, although read-write access is further limited to specific directories in a regular and well-defined fashion. Projects handling PHI or PII will in general need access to at least two project spaces, one for datasets including sensitive identifiers, one for datasets stripped of such identifiers. Given the level of CMS data re-use and staffing constraints it is not uncommon to have three to five levels of project groups associated with a given research team, some spanning research teams to support the development of common data resources.

2.5. Explain the use of technical safeguards for data access (which may include password protocols, log-on/log-off protocols, session time out protocols, and encryption for data in motion and data atrest).

Passwords are required to be reasonably long and complex, striking a balance between recall limits and technical requirements. Passwords must be changed frequently, and a common password is used across all interactive systems. Some systems used by junior staff do enforce session timeout limits, but in general staff are permitted to work after hours and thus systems are operational 24 x 7. Encryption within the protected systems would provide no additional security and is not used.

The standard interface for most users is expected, but not required, to be a Windows system, either a local desktop or a remote desktop session on a central server. All Center owned machines are joined into a Windows Domain, specific to the Center and locally managed. Idle session screen locks are provided by GPO, but users are able to change limits if they wish. Idle sessions are never terminated since some software becomes “idle” without saving intermediate results, and thus any form of automatic session termination would cause lost work product. Similarly, which the terminal servers will lock the remote desktop session when idle, they do not disconnect the session.

By policy, any confidential data, of which all CMS acquired under a DUA is a subset, shall not be stored on a local system. This includes laptops, desktops, mobile devices of other sorts, external storage devices, etc. The central servers are secured by multiple layers of physical security. Similarly the Center’s network is isolated behind multiple layers of switches and firewalls. Data at rest on server disks is not encrypted except insofar as it may be prepared for a data transfer. Recognizing our poor disaster recovery posture since unencrypted tapes cannot be securely exported from the server room, we’re engaged in a process of adding encryption to our backup system.

Data transfers within the Center’s local network are not encrypted, partly due to speed concerns, partly as a matter of platform compatibility. The Center’s local network is blocked from allowing Windows or Unix native file sharing method using standard ports. Data transfers for confidential data outside of the network are prohibited, but terminal server traffic is always natively encrypted. All network traffic beyond the Brown campus network boundary requires further encryption, generally involving an SSL channel.

2.6. Are additional organizations involved in analyzing the data files provided by CMS? No

If so, please review the Collaborator Checklist for guidance and considerations to include in the Data Management Plan, and indicate below how these organizations’ analysts will access the data files:

- ☐ VPN connection
- ☐ Will travel to physical location of data files at requesting organization
- ☐ Request that a copy of the data files be housed at second location
- ☐ Other: Click here to enter text.

2.7. If an additional copy of the data will be housed in a separate location, please describe how the data will be transferred to this location. (Also, please ensure you have included information on this organization’s database management under the appropriate subsections of the database management plan.)

One complete set of tapes will be held externally for Disaster Recovery purposes in a secure vault maintained by Iron Mountain at their Rhode Island offsite facility located in Chepachet, RI, 02814. The one offsite copy will be picked up by an Iron Mountain courier for storage in their facility, such copy to consist of a box of tapes in a container secured with a locking tag. After the first copy has been transferred a monthly swap will occur via the Iron Mountain courier. Given the media are encrypted there seems minimal operational risk in allowing staff working for our Director of Operations to hold the monthly box of media in an external safe for the brief times between removal from the server room and acceptance by/receipt from the Iron Mountain courier.

3. DATA REPORTING AND PUBLICATION

3.1. Who will have the main responsibility for notifying CMS of any suspected incidents wherein the security and privacy of the CMS data may have been compromised? Please describe and identify your organization's policies and procedures for responding to potential breaches in the security and privacy of the CMS data.

Brown's Computing Information Services: Policy on the Handling of Brown Restricted Information /computing-policies/policy-handling-brown-restricted-information and the Human Research Protection Program Policy and Procedure Manual (https://www.brown.edu/research/conducting-research-brown/preparing-proposal/research-integrity/hrppirb-home-page/brown-univ-pol-proc) require the User to notify any or all of the CIS ISIRT team, the Brown IRB, and CMS of known or likely exposure of CMS PII data, and conversely CIS may notify the User of a suspected security breach (https://it.brown.edu/information-security/protect-university-data/brown-university-cybersecurity-program). Specific notification to CMS per the requirements of this DUA falls to Center systems management as technical owner of the data. It is assumed that this would occur via the CMS incident reporting hotline. Notification will be made within 1 hour of the discovery of any security breach. Note, however, that the term "potential" above is disturbing; we will report any and all breaches where there is a reasonable likelihood that any specific CMS data, covered by this or any other DUA, has been exposed to unauthorized users, and in particular to unknown or known but non-Center users. Brown maintains extensive network and systems monitoring systems and we routinely update and improve our security posture.

3.2. Explain how your organization's data management plans are reviewed andapproved.
Approval is granted by Center senior management to the systems manager to select, develop, and maintain the security infrastructure for the core systems, as well as other technical and functional supporting systems. PIs, acting as functional data owners, work with systems and programming staff, as technical data owners, to manage project data. No Center formal review and approval process has been implemented, nor would one be appropriate. The Brown IRB does review and approve research proposal involving PHI or PII with respect to data security and confidentiality safeguards in the context of each specific research project, as does each data owner with which a PI holds a DUA for a specific project.

3.3. Explain whether and how your organization's data management plans are subjected to periodic updates during the DUA period.
Data management plans are updated by PIs in response to changing conditions and competing priorities. The Center does not assert ownership of this process although we have instituted a limited annual review for each project with the PI, aligned with yearly CMS expiration dates to confirm the personnel and data approved under a particular DUA. In addition, certain types of DUA amendment requests require a re-review of the data management plan by CMS.

3.4. Please attest to the CMS cell suppression policy of not publishing or presenting tables with cell sizes lessthan 11. (see Item 9 of the DUA) I agree.

4. COMPLETION OF RESEARCH TASKS AND DATA DESTRUCTION

4.1. Describe your organization's process to complete the Certificate of Disposition form and policies and procedures to dispose of data files upon completion of its research.

The Certificate of Disposition is filled out in accordance with CMS procedures. Data transfer media are mechanically destroyed locally (e.g., CD-ROM media are shredded), shredded by a bonded data destruction contractor managed by Brown, or sanitized by writing random data multiple times (in the case of removable and reusable disk drives). All copies of primary data containing PHI or PII is erased from all active systems. PIs

Brown University IRB Original Approval: 12/10/2020
Brown University IRB Amendment Approval 02/25/2021
Brown University IRB Amendment Approval 05/11/2021
Brown University IRB Amendment Approval 08/19/2021
Brown University IRB Amendment Approval 10/06/2021

are responsible for the destruction of any derivative data files, which should have been stored in specific project spaces associated with their DUA or other project scope, in compliance with CMS policies.

Ultimately, the Custodian is responsible for certifying the disposition of CMS data, in accordance with our standard business practices. Once signed, the form is handed over to our DUA Manager, Julie Lima, who submits the formal request for DUA closure to CMS or the appropriate federal project officer or DUA Requester with instructions to submit to CMS following current CMS policy found at https://www.cms.gov/Research-Statistics-Data-and-Systems/Files-for-Order/Data-Disclosures-Data-Agreements/DUA_-_Extensions_Closures.htmlNote that all Center staff are covered by Brown Policy on the handling of sensitive data, and such Policies are written and maintained under the auspices of the office of the Provost. In particular, such policies imply and require that staff not undertake to circumvent data destruction mechanisms. Such Policy is freely available for review by all staff at any time. The Center is undertaking to amplify that Policy with details internal to our business practices, in order to clarify the procedure requirements for adhering to those policies. To a first approximation, that means diligently erasing any all data covered by a DUA or IRB agreement at the termination of such agreement, except for such retentions as allowed by suchagreements.

4.2. Describe your organization's policies and procedures used to protect CMS data files when individual staff members of research teams (as well as collaborating organizations) terminate their participation in research projects (which may include staff exit interviews and immediate accesstermination).

The Policy Relating to Research Privacy of the Office of the Provost, referenced above, in concert with the Brown Acceptable Use Policy for computing resources, requires that no use be made of any CMS data files after a staff person's association with a research project is terminated. As a matter of Center Policy, as being developed to meets CMS's requirements for auditable staff allocations all research staff will have signed a confidentiality agreement covering their use of CMS data. Our Center is currently considering putting a time limit on such agreements and requiring regular renewal. Staff access to sensitive data is terminated as soon as systems management staff is notified by the PI that a staff member's participation in a project has terminated. Note that no work product destruction is ever undertaken by system staff, and thus access termination is a relatively light-weight process, should a staff member's participation in a project resume. Language has been added to the Center User's Guide reinforcing these points.
Note that we do not currently have a policy which requires an exit interview relevant to data security. Staff have no physical devices with any security significance and it would be a violation of policy to have a local copy of any confidential dataset, including a CMS dataset. Conveniently, the Custodian is also the senior system administrator, so as soon as he is notified by the PI or other staff (eg. HR admin), access is terminated as soon as possible. Practically, this means within minutes of notification. PIs and other Investigators may of course require exit interviews as a part of other business processes; the security plan does not encompass workflow management. Note that revoking staff access to a protected project space can be accomplished without removing them from all access to Center systems. Such removals are made in the same manner as terminations, that is, upon receipt of a request for such removal by a PI to the Custodian.

4.3. Describe policies and procedures your organization uses to inform CMS of project staffing changes, including when individual staff member's participation in research projects is terminated,voluntarily or involuntarily.

The Policy Relating to Research Privacy of the Office of the Provost requires, through its reference of the IRB approval process, that changes to the Principal Investigator (PI) be reported to the IRB. Separately, each DUA between CMS and a researcher requires that CMS be informed of staffing changes relating to those staff members with direct and unsupervised access to CMS data. Procedurally, our Center PIs normally directly contact our DUA coordinator, currently Julie Lima, about staff changes and appropriate signature addenda are

filed with CMS either directly or via a federal project officer as applicable. She will then contact CMS in a timely manner via e-mail to notify them of the staffing change.

4.4. Describe your organization's policies and procedures to ensure original data files are not used following the completion of the project.

General Brown Policies regarding appropriate data use are binding upon all faculty and staff of Brown University. In particular:

Ultimately the "Policy on the Handling of Brown Restricted Information" (https://it.brown.edu/computing-policies/policy-handling-brown-restricted-information) defines in general terms the need to protect human subjects data, which would include any data received from CMS under a DUA.

That in turn references "Policies and Procedures Relating to Research Privacy" (http://www.brown.edu/Administration/Provost/policies/rpp.html) which defines in general terms the requirements for defining and controlling access to PHI data.

Somewhat tangentially, data received from CMS under a DUA may be viewed as the property of CMS, even if not personal health information. The use of such data is covered in general terms by the "Acceptable Use Policy" (http://www.brown.edu/cis/policy/aup.php) which requires compliance with the requirements of the data's owner.

The Policy Relating to Research Privacy of the Office of the Provost, referenced above, in concert with the Brown Acceptable Use Policy for computing resources, requires that no use be made of any data acquired under control of this or any other CMS DUA beyond the termination of that DUA. Therefore, by Brown Policy, there is no authorized access to data files beyond completion of their project. The Center's User's Guide emphasizes these points. (Note that in all cases data is authorized for use in a specific fashion in the context of a specific project. If CMS authorized re-use of data in an additional project within the Center, access to that data is tracked separately in the context of each project.) Procedurally, data is erased from all systems as part of the destruction process. Since the same staff receive, process, and destroy the data, no technical controls are applied beyond employing safety features such as disk high water marking. Similarly, PIs and staff are responsible for storing derivative datasets in project-specific locations secured to limit access to only project staff, and to erase any sensitive datasets as part of study termination unless the entire project space is being erased. (This latter is not normally done, but rather the project space is "dehydrated" so that it only contains residual work product such as program code. Such derivative files often need to be referenced for years after the termination of all Data Use Agreements.)

PRA Disclosure Statement

According to the Paperwork Reduction Act of 1995, no persons are required to respond to a collection of information unless it displays a valid OMB control number. The valid OMB control number for this information collection is 0938-XXXX . The time required to complete this information collection is estimated to average two hours per response, including the time to review instructions, search existing data resources, gather the data needed, and complete and review the information collection. If you have comments concerning the accuracy of the time estimate(s) or suggestions for improving this form, please write to: CMS, 7500 Security Boulevard, Attn: PRA Reports Clearance Officer, Mail Stop C4-26-05, Baltimore, Maryland 21244-1850.

COLLABORATOR CHECKLIST

Please note –This checklist is for guidance purposes only and for organizations that are involving an additional organization as part of their research study. The checklist identifies data safeguard practices and considerations of the collaborating organization that should be indicated in the data requestor's Data Management Plan. All questions may not apply but are dependent upon the data sharing arrangement between the organizations involved in the research study.

(Information that should be indicated for each collaborating organization that will have access to RIF or non-identifiable files.)*

A. Access to Identifiable and De-identifiable Files

1. What is the name of the collaborating organization?*
   Click here to enter text.
2. How will the collaborating organization access the CMS data (secure VPN, a physical copy on site at the collaborating organization, traveling to the DUA holder's site, etc.)?*
   Click here to enter text.
3. Who are the researchers from the collaborating organization? Indicate if each researcher will have access to raw data, analytic files, or output with cell sizes less than 11. (*Please ensure that these individuals and data access rights are listed in the Project Staff list.*)*
   Click here to enter text.
4. What binding agreements are required of the researchers from the collaborating organization?*
   Click here to enter text.
5. What training is required of researchers from the collaboratingorganization?*
   Click here to enter text.
6. How will the collaborating organization notify the DUA holder of changes in staff who are participating on the research team?*
   Click here to enter text.
7. Will the researchers from the collaborating organization abide by the DUA holder's project rules or the policies of their employing organization?*
   Click here to enter text.

B. Access to RIF

1. Will the collaborating organization have access to RIF?*
   Click here to enter text.

   If yes, please provide the following required details:
   a. Will the collaborating organization have the ability to download and store a copy of the CMS data?
      Click here to enter text.
   b. Does the collaborating organization intend to backup the data? If so, has the collaborating organization developed a backup arrangement and are the back-up copies maintained at a secondlocation?
      Click here to enter text.
   c. Who is responsible for maintaining the security and distribution of the CMS data at thecollaborating organization?
      Click here to enter text.

d. Does the collaborating organization maintain an inventory of the CMS data files that are maintained by the collaborating organization?
Click here to enter text.

e. How will the collaborating organization tailor and restrict dataaccess?
Click here to enter text.

f. Please describe the collaborating organization's physical and technical safeguards used to protect CMS data files (including physical access and logical access to the files).
Click here to enter text.

g. Please describe the collaborating organization's infrastructure, operating systems, and hardware that will be used to secure the CMS data.
Click here to enter text.

h. How will the collaborating organization dispose of electronic copies of thedata?
Click here to enter text.

C. Physical Copies of RIF

Please note - if the collaborating organization will maintain a separate copy of the CMS data, the collaborating organization is required to complete a full Data Management Plan.

1. Will a separate copy of the CMS data be housed at the collaborating organization's location?
Click here to enter text.

2. How will the collaborating organization receive the CMS data (shipment from the DUA holder, collaborating organization will request an additional copy directly from CMS, POC from the collaborating organization will transport the data, etc.)?
Click here to enter text.

COLLABORATOR CHECKLIST

Please note –This checklist is for guidance purposes only and for organizations that are involving an additional organization as part of their research study. The checklist identifies data safeguard practices and considerations of the collaborating organization that should be indicated in the data requestor's Data Management Plan. All questions may not apply but are dependent upon the data sharing arrangement between the organizations involved in the research study.

(Information that should be indicated for each collaborating organization that will have access to RIF or non-identifiable files.)*

A. Access to Identifiable and De-identifiable Files

8. What is the name of the collaborating organization?*

9. How will the collaborating organization access the CMS data (secure VPN, a physical copy on site at the collaborating organization, traveling to the DUA holder's site, etc.)?*
   Secure VPN

10. Who are the researchers from the collaborating organization? Indicate if each researcher will have access to raw data, analytic files, or output with cell sizes less than 11. (*Please ensure that these individuals and data access rights are listed in the Project Staff list.*)*
    Dr.

11. What binding agreements are required of the researchers from the collaboratingorganization?*
    has signed the Data Protection and Security Agreement as detailed in section 1.3 of the data management plan.

12. What training is required of researchers from the collaboratingorganization?*
    Dr. is up to date on their CITI training.

13. How will the collaborating organization notify the DUA holder of changes in staff who are participating on the research team?*
    Dr. will inform the PI by email or phone of any changes in staff as soon as they becomes aware of such changes.

14. Will the researchers from the collaborating organization abide by the DUA holder's project rules or the policies of their employing organization?*
    Yes, Dr. will abide by the DUA holder's project rules and policies.

B. Access to RIF

2. Will the collaborating organization have access to RIF?*
   No – they will not have access to the RIF. Dr. will access to RIF by VPN-ing into Brown's secure network. They will NOT copy or otherwise transfer any data from that network to the University of

   If yes, please provide the following required details:

   a. Will the collaborating organization have the ability to download and store a copy of the CMS data?
      Click here to enter text.
   b. Does the collaborating organization intend to backup the data? If so, has the collaborating organization developed a backup arrangement and are the back-up copies maintained at a secondlocation?
      Click here to enter text.
   c. Who is responsible for maintaining the security and distribution of the CMS data at thecollaborating organization?
      Click here to enter text.

d. Does the collaborating organization maintain an inventory of the CMS data files that are maintained by the collaborating organization?
Click here to enter text.

e. How will the collaborating organization tailor and restrict dataaccess?
Click here to enter text.

f. Please describe the collaborating organization's physical and technical safeguards used to protect CMS data files (including physical access and logical access to the files).
Click here to enter text.

g. Please describe the collaborating organization's infrastructure, operating systems, and hardware that will be used to secure the CMS data.
Click here to enter text.

h. How will the collaborating organization dispose of electronic copies of thedata?
Click here to enter text.

C. Physical Copies of RIF

Please note - if the collaborating organization will maintain a separate copy of the CMS data, the collaborating organization is required to complete a full Data Management Plan.

3. Will a separate copy of the CMS data be housed at the collaborating organization's location?
No, a separate copy of the CMS data will not be housed at the collaborating organization's location.

4. How will the collaborating organization receive the CMS data (shipment from the DUA holder, collaborating organization will request an additional copy directly from CMS, POC from the collaborating organization will transport the data, etc.)?
Click here to enter text.

Office of Research Integrity
Box 1986
Providence, RI 02912
Tel: (401) 863-3050
Fax: (401) 863-7292

Email: IRB@Brown.edu
FWA# 00004460
IRB# 00000556

IRB Amendment Approval

**To:**	Kali Thomas, Box G-S121-6
**From:**	Human Research Protection Program
**Date:**	October 06, 2021
**Protocol:**	Home-delivered meals for persons with dementia: Which model delays nursing home placement?
**Protocol #:**	2008002788
**Amendment #:**	5

Approval Date: October 06, 2021

This amendment was approved by expedited procedures in accordance with 45 CFR 46.111.

Approval of this amendment (memo dated:10/01/2021) includes:
1. modify the language in the protocol to reflect that participants are receiving meals delivered multiple times a week by the MOW program, rather than daily-delivered meals.
2. Revise one error in the protocol, where it should read biweekly frozen meal delivery rather than weekly frozen meal delivery. Participants are still receiving the same amount of meals in both arms.
3. Modify the recruitment targets for two sites - Neighborly and MOW-San Antonio. We would like to modify the target goals from 60 to Neighborly to 45-60, and MOW-SA from 100 to 100-115.

Any changes to this research protocol must be submitted to the IRB for review and approval prior to implementation. While this study is active, compliance with any post-approval monitoring activities and all Brown University HRPP policies and procedures, including the *Reportable Events Policy,* are required.

All study staff must successfully complete CITI training in human subjects research prior to beginning work on this project.
