# Supplementary material for: Home-Delivered Meals and Nursing Home Placement Among People With Self-Reported Dementia: A Pilot Pragmatic Clinical Trial
Source: JAMA Netw Open. 2023 Dec 20;6(12):e2347195. doi: 10.1001/jamanetworkopen.2023.47195 (PMC10733798; doi:10.1001/jamanetworkopen.2023.47195)
Supplement: Supplement 2. — eTable. Baseline Characteristics of Analyzed Participants, by Study Arm [file jamanetwopen-e2347195-s002.pdf]

Supplemental Online Content

Thomas KS, Bunker J, Gadbois E, et al. Home-delivered meals and nursing home placement among people with self-reported dementia: a pilot pragmatic clinical trial. *JAMA Netw Open*. 2023;6(12):e2347195. doi:10.1001/jamanetworkopen.2023.47195

eTable. Baseline Characteristics of Analyzed Participants, by Study Arm

This supplemental material has been provided by the authors to give readers additional information about their work.

© 2023 Thomas KS et al. *JAMA Network Open*.

eTable. Baseline Characteristics of Analyzed Participants, by Study Arm

	Drop-Shipped, Frozen Meals (n=112 95% CI 110,114)*	Daily-Delivered Meals (n=126 95% CI 124,127)*
Age Group**		
66-74	23.5% (15.6%,31.3%)	28.0% (20.0%,35.9%)
75-84	40.0% (30.1%,49.1%)	39.4% (30.1%,47.9%)
85+	36.5% (27.6%,45.5%)	32.6% (24.4%,40.8%)
Female**	67.9% (59.2%,76.6%)	57.4% (48.8%,66.1%)
Male**	32.1% (23.4%,40.8%)	42.6% (33.9%,51.2%)
Race / Ethnicity**		
Black	18.3% (11.1%,25.4%)	16.4% (9.9%,22.9%)
Latino or Hispanic Ethnicity	22.1% (14.4%,29.8%)	29.4% (21.4%,37.4%)
White	56.1% (46.9%,65.3%)	49.4% (40.8%,58.2%)
Lived Alone**	57.8% (48.6%,66.9%)	41.2% (32.5%,49.8%)

Data originates from sites' intake assessments completed for all new people added to the waiting list.

*Totals represents the average number of participants across multiply imputed linkage structures and the number in parentheses represents the 95% credible interval across linkage structures for the 16 probabilistically linked individuals.

**Values in parentheses represent 95% confidence intervals that account for errors in the linking of 16 participants and sampling errors.

© 2023 Thomas KS et al. *JAMA Network Open*.
