# Supplementary material for: Home-Delivered Meals and Nursing Home Placement Among People With Self-Reported Dementia: A Pilot Pragmatic Clinical Trial
Source: JAMA Netw Open. 2023 Dec 20;6(12):e2347195. doi: 10.1001/jamanetworkopen.2023.47195 (PMC10733798; doi:10.1001/jamanetworkopen.2023.47195)
Supplement: Supplement 3. — Data Sharing Statement [file jamanetwopen-e2347195-s003.pdf]

Data Sharing Statement

Thomas. Home-Delivered Meals and Nursing Home Placement Among People With Self-Reported Dementia. *JAMA Netw Open*. Published December 20, 2023. doi:10.1001/jamanetworkopen.2023.47195

Data
Data available: No

Additional Information
Explanation for why data not available: The outcomes data for this trial is provided to investigators under a strict data use agreement with the Centers for Medicare and Medicaid Services
